# Design and synthesis of new quinazolinone derivatives: investigation of antimicrobial and biofilm inhibition effects

**DOI:** 10.1007/s11030-024-10830-y

**Published:** 2024-04-24

**Authors:** Rasha Mohamed Hassan, Heba Yehia, Mohammed F. El-Behairy, Aida Abdel-Sattar El-Azzouny, Mohamed Nabil Aboul-Enein

**Affiliations:** 1 Medicinal and Pharmaceutical Chemistry Department, Pharmaceutical and Drug Industries Research Institute, National Research Centre (ID: 60014618), P.O. 12622, Dokki, Giza, Egypt; 2 Chemistry of Natural and Microbial Products Department, Pharmaceutical and Drug Industries Research Institute, National Research Centre (ID: 60014618), P.O. 12622, Dokki, Giza, Egypt; 3 Department of Organic and Medicinal Chemistry, Faculty of Pharmacy, University of Sadat City, 32897, Sadat City, Egypt

**Keywords:** Quinazolinones, Antimicrobial, Biofilm inhibition, *Pseudomonas aeruginosa*

## Abstract

New quinazolin-4-ones **9–32** were synthesized in an attempt to overcome the life-threatening antibiotic resistance phenomenon. The antimicrobial screening revealed that compounds **9**, **15**, **16**, **18**, **19**, **20** and **29** are the most broad spectrum antimicrobial agents in this study with safe profile on human cell lines. Additionally, compounds **19** and **20** inhibited biofilm formation in *Pseudomonas aeruginosa*, which is regulated by quorum sensing system, at sub-minimum inhibitory concentrations (sub-MICs) with IC_50_ values 3.55 and 6.86 µM, respectively. By assessing other pseudomonal virulence factors suppression, it was found that compound **20** decreased cell surface hydrophobicity compromising bacterial cells adhesion, while both compounds **19** and **20** curtailed the exopolysaccharide production which constitutes the major component of the matrix binding biofilm components together. Also, at sub-MICs *Pseudomonas* cells twitching motility was impeded by compounds **19** and **20**, a trait which augments the cells pathogenicity and invasion potential. Molecular docking study was performed to further evaluate the binding mode of candidates **19** and **20** as inhibitors of *P. aeruginosa* quorum sensing transcriptional regulator PqsR*.* The achieved results demonstrate that both compounds bear promising potential for discovering new anti-biofilm and quorum quenching agents against *Pseudomonas aeruginosa* without triggering resistance mechanisms as the normal bacterial life cycle is not disturbed.

**Graphical abstract:**

New 4-quinazolinones were synthesized and screened for their antimicrobial activity. Compounds **19** and **20** inhibited biofilm formation in *Pseudomonas aeruginosa* at sub- minimum inhibitory concentrations. Also, they decreased other virulence factors at low concentrations without affecting bacterial growth bacteria indicating their promising profile as anti-virulence agents that cause less bacterial resistance than the conventional antibiotics.

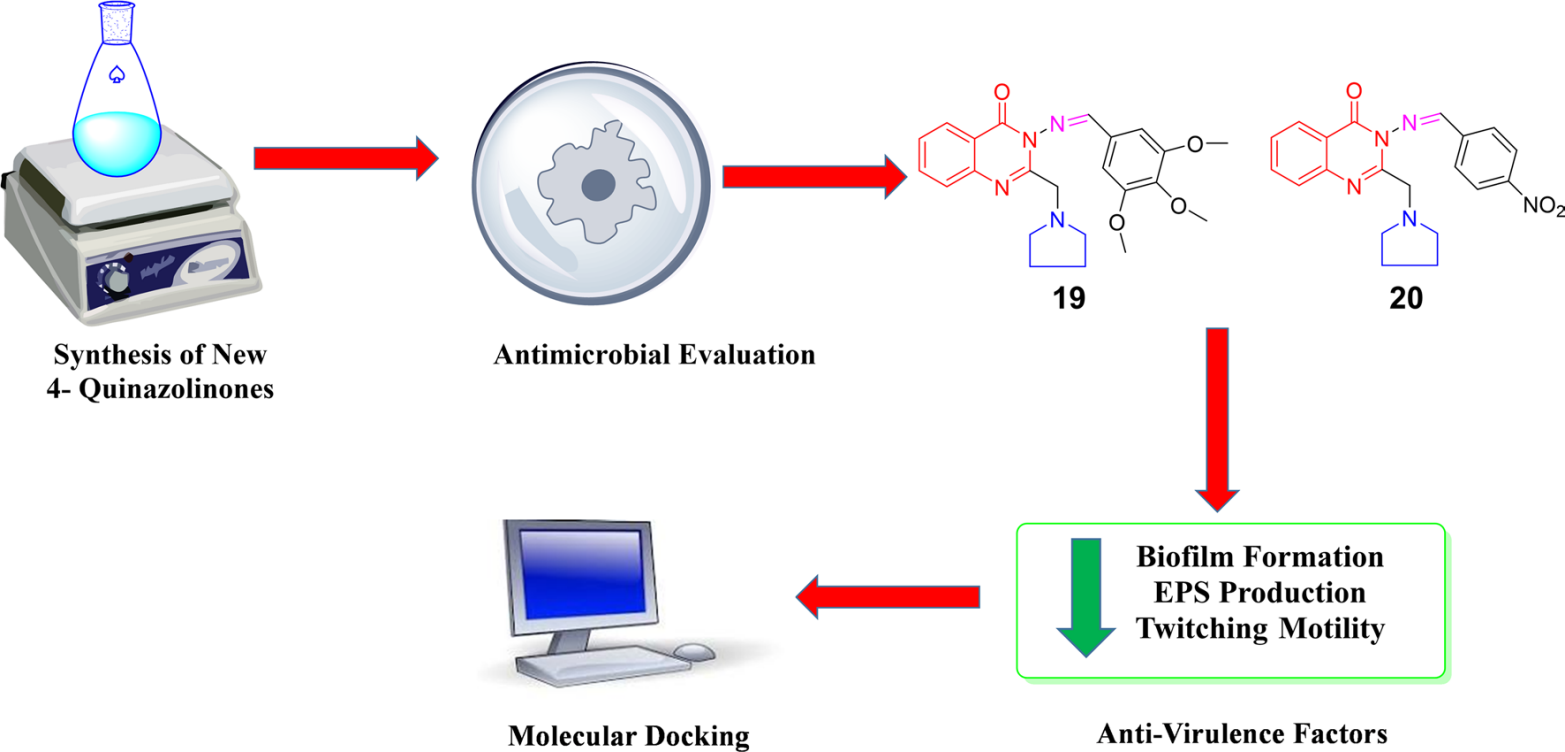

**Supplementary Information:**

The online version contains supplementary material available at 10.1007/s11030-024-10830-y.

## Introduction

The tremendous increase in the microbial resistance to the marketed therapeutic agents is the driving motivation for the development of new drugs for the management of infectious diseases [1, 2] specially that they are often reported with cancer and cardiovascular diseases as the major causes of mortality and morbidity worldwide, while they bear the additional gravity of being communicable [3]. Hence, their risk is not only restricted to the subjects in contact with contaminated environments but also keep spreading to further circles via direct or indirect transfer. In the war against bacterial resistance, it is not enough anymore to develop new antibiotics with a distinct mechanism of action, e.g.: disrupting synthesis of proteins, nucleic acid or cell wall, metabolic pathways interruption, cell membrane structure or performance disintegration [3, 4]. Instead, one of the promising approaches is to target bacterial cells communication mechanism, commonly known as quorum sensing (QS) [1, 5]. QS controls bacterial cells coordination through signal molecules called autoinducers which stimulate specific receptors and consequently, regulate several biochemical processes in bacteria via transcriptional signals. These processes are the ones that mainly govern pathogenesis and involve, but are not limited to, bacterial motility, expression of virulence factors, biofilm formation, and antibiotics resistance [6–8]. Accordingly, annihilation of quorum sensing network, with or without influencing bacterial growth, is per se an efficient strategy to disrupt cumulative production of virulence factors and debilitate the pathogenicity of bacteria [9]. Interestingly, molecules which inhibit virulence factors and biofilm formation in bacteria, without affecting bacterial growth, are less likely to trigger resistance mechanisms in comparison to conventional antibiotics due to the absence of the selective pressure. This occurs as cells do not acquire induced resistance and do not pass down the relevant genetic material [3]. Ideally, these agents should not interfere with bacterial and host metabolism or lead to any harmful side effects to be eligible for clinical application [10].

*Pseudomonas aeruginosa* is classified as a Gram-negative infectious bacterium. It is one of the most opportunistic bacteria and is responsible for several human infections, especially in immunocompromised and elderly patients, e.g.: urinary tract, burn and implant infections as well as chronic otitis. *P. aeruginosa* infection is mostly accompanied with respiratory failure, decreased pulmonary function and high mortality rate within cystic fibrosis patients [11, 12]. The severity of *P. aeruginosa* infections is due to the metabolic adaptation of the bacteria which are supported via many virulence factors and enzymes including transcriptional regulators, proteases as well as lipopolysaccharides [8]. In addition, *P. aeruginosa* possesses the capability of colonizing surfaces in the form of biofilm communities; a trait which decreases the effectiveness of antibiotics, shields bacterial cells against hosts’ immune systems in vivo, and leads to antibacterial resistance by means of potentiated horizontal transfer of acquired resistance genes, e.g.: antibiotic-modifying enzymes, specific efflux pumps, decreased cell penetration, etc. [3, 4].

**Fig. 1:**
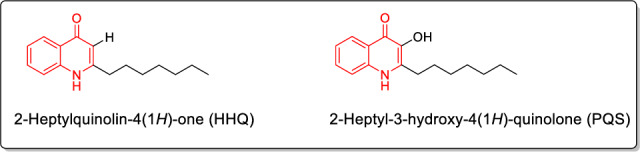
2-Alkyl-4-quinolone (AQ) molecules acting as autoinducers of pqs system in *Pseudomonas aeruginosa*

The QS system in *P. aeruginosa* consists of three interlinked systems namely, *las*, *rhl* and *pqs*, which play an important role in bacterial pathogenicity. Each cascade comprises a transcriptional regulator protein namely; LasR, RhlR and PqsR, respectively [13]. Both LasR and RhlR systems operate through diffusion of *N*-acyl homoserine lactone as signaling or inducing molecules, whereas the PqsR (also named MvfR) system utilizes two alkylquinolones as autoinducers (Fig. 1) [14].

**Fig. 2:**
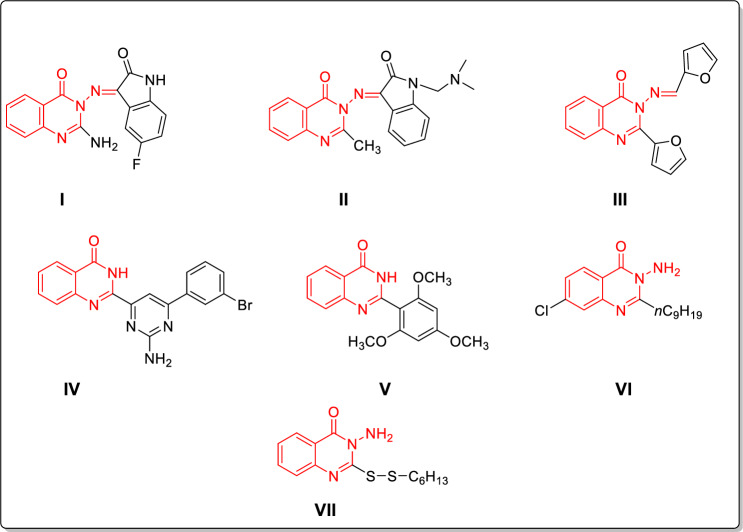
Examples of 4(3*H*)-quinazolinones hybrids having antimicrobial and anti-biofilm effect

Quinazolinones have enriched the medicinal chemistry library with a huge number of bioactive molecules which possess different biological activities such as analgesic [15], anti-inflammatory [15, 16], anti-hyperlipidemic [17, 18] and anticancer agents [19–21]. Aiming to overcome the hazards of microbes that are resistant to the available antimicrobial agents, extensive studies have disclosed the promising antibacterial and antifungal properties of 4(3*H*)-quinazolinone derivatives [22–25]. Following this track, various 4(3*H*)-quinazolinone Schiff’s base hybrids, derived from hydrazine, as compounds **I**-**III** [26–28] have been reported to display significant antimicrobial activities (Fig. 2).

Additionally, the 4(3*H*)-quinazolinones are considered as one of the major classes that suppress biofilm formation. In this vein, the pyrimidin-4-yl quinazolin-4(3H)-one conjugate I**V** could efficiently inhibit biofilm formation in methicillin-resistant *Staphylococcus aureus* (MRSA) with IC_50_ = 20.7 μM [29]. Furthermore, the quinazolinone derivatives as compound **V** have anti-biofilm activity against *Candida albicans* with IC_50_ less than 30 μM [30]. Moreover, the quinazolinone-based derivatives as compounds **VI** and **VII** are inhibitors of *P. aeruginosa* quorum sensing transcriptional regulator PqsR and accordingly, attenuate biofilm formation [31, 32] (Fig. 2).

**Fig. 3:**
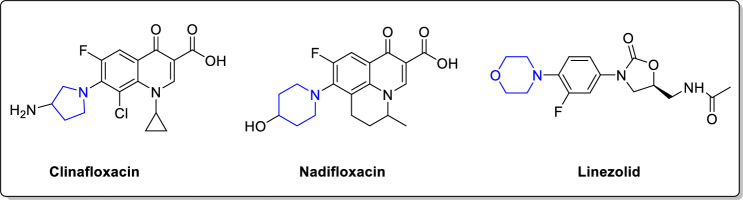
Antibacterial drugs containing alicyclic amines

On the other hand, pyrrolidine, piperidine and morpholine are valuable saturated secondary amines that found in many antibacterial drugs as clinafloxacin, nadifloxacin and linezolid (Fig. 3). Moreover, alicyclic amines have fostered the antimicrobial potency of chemically synthesized compounds [33, 34]. Interestingly, one of the most common metabolic pathways of alicyclic amines is α-carbonyl formation leading to lactam structures [35] which are well known pharmacophores in antibacterial agents.

**Fig. 4:**
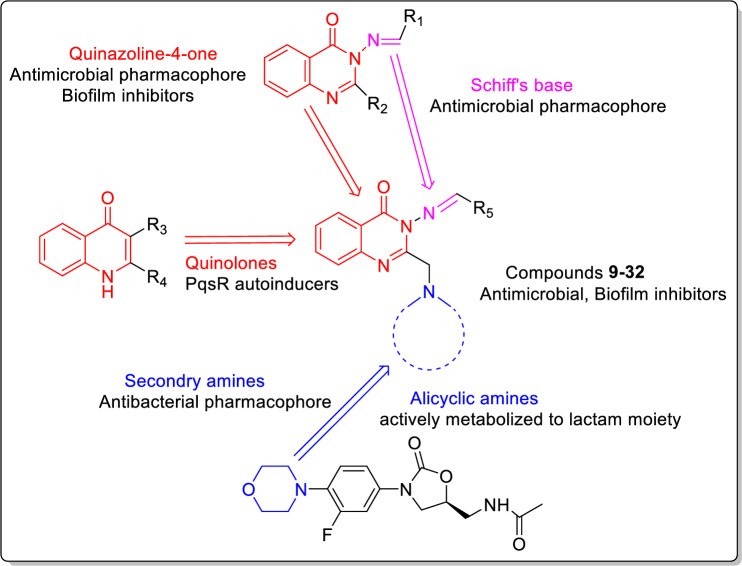
Rational design of our candidates **9**–**32**

Diverse substituents at 2-position of quinazoline-4-ones have displayed interesting antibacterial activity as per compounds **I-VII** (Fig. 2). Also, secondary/alicyclic amines have contributed positively to the potency of many antibacterial candidates (Fig. 3). In the same vein, imine bond (Schiff’s base) has been utilized as a linker in many candidates that showed antibacterial potential (compounds **I-III**, Fig. 1). Therefore, in the current work, it has been decided to design hybrid structures (**9**–**32**) containing the aforementioned moieties that were endowed with antibacterial activity. Hence, quinazolin-4-one has been hybridized at position 2 with secondary/alicyclic amines while diverse hydrophobic groups at position 3 were linked via Schiff’s base to afford compounds **9**–**32** (Fig. 4). Assessing the effect of substituting different open chain **9**–**14** or alicyclic secondary amines **15**–**32** on their antimicrobial activity was also studied (Fig. 4). It is worth mentioning that the designed compounds also share structural similarity with the quinolones autoinducers of the pqs system of *P. aeruginosa.* The design was thus realized with the objective of evaluating the most active compounds in inhibiting the *P. aeruginosa* biofilm formation and other virulence factors at sub-MICs and thus, fulfilling the hypothesis that the pathogenicity decline should not be followed by prompting antibiotic resistance mechanisms. Molecular docking study was carried out to determine the binding mode of candidates **19** and **20** as inhibitors of *P. aeruginosa* quorum sensing transcriptional regulator PqsR*.*

## Results and discussion

### Chemistry

**Scheme 1:**
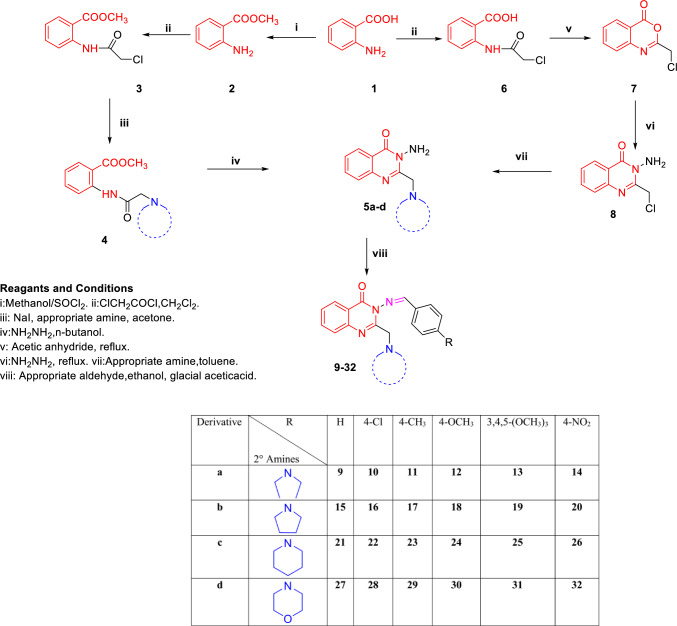
Synthesis of target compounds **9–32** and key intermediates

The synthesis of compounds **9–32** and their intermediates is depicted in Scheme 1. The key intermediates **5a-d** were prepared by two routes. The first one involves the amidation of the anthranilic acid ester **2** using 2-chloroacetylchloride following the classical triethylamine catalyzed amidation [36] to obtain the amide derivative **3**. The latter was allowed to react with NaI to give the iodo derivative then two molar equivalent of the secondary aliphatic or alicyclic amines have been added to afford compounds **4a, c** and **d** [37]. Unfortunately, compound **4b** was obtained in very low yield via this route. Cyclization of methyl anthranilate-*N*-amide to quinazolin-4-one derivatives is reported in presence of hydrazine hydrate under diverse catalytic conditions for example, 1-butyl-3-methylimidazolium tetrafluoroborate in water [38, 39], phosphorus trichloride in tetrahydrofuran [40], triethylammonium acetate; in microwave irradiation [41], barium hydroxide octahydrate, phosphorus trichloride in tetrahydrofuran [42], ethanol under microwave irradiation [40] and the most simple method was using butan-1-ol and heating [37, 43]. Thus, compounds **4a, c** and **d** were cyclized to the key quinazolin-4-one derivatives **5a, c**, and **d** via reaction with hydrazine hydrate in n-butanol.

Due to the unsatisfactory yield of preparation of intermediate **4b**, another route has been accomplished where the cyclization step was performed prior to the nucleophilic replacement of chloride with secondary amines. Hence, the anthranilic acid *N*-amide **6** was prepared from anthranilic acid (**1**) following the same procedure of preparing **3** from **2**. Cyclization of **6** to benzoxazine-4-one **7** was carried out using acetic anhydride as a dehydrating agent [44–46]. The quinazolin-4-one **8** was obtained via reaction of benzoxazine-4-one **7** with hydrazine hydrate, however the yield was unsatisfactory for this reaction (30%). Compound **8** was then converted to compound **5a-d** via nucleophilic substitution of chloride at position 2 with excess secondary aliphatic or alicyclic amines to avoid effect of strong base like potassium carbonate on the starting materials. Target compounds **9–32** were then achieved using the well-established Schiff’s base synthesis from reaction of primary amine and aldehyde under acidic conditions.

^1^H-NMR of compounds **9–32** demonstrated the disappearance of the NH_2_ singlet of compounds **5a-d** at δ_H_ 3.5–4.00 ppm and the presence of the characteristic imine proton at δ_H_ 8.50–9.00 ppm. Also, the number of aromatic protons has been increased by a number equivalent to the aldehyde protons while the singlet for 2H of the methylene bridge still noticeable but shifted downfield from δ_H_ 3.00–3.5 in 5a-d to δ_H_ 3.50–4.00 ppm in **9–32**. In ^13^C-NMR, three characteristic signals have been observed δ_c_ 150–170 ppm corresponding to the carbonyl and C=N of the imine and quinazoline ring.

## In vitro studies

### Antimicrobial evaluation

The antimicrobial activity of the new quinazolinone derivatives **9**–**32** was determined against three Gram-positive bacteria: *Bacillus cereus* (*B. cereus* ATCC 6629), *Bacillus subtilis* (*B. subtilis* ATCC 6633) and *Staphylococcus aureus* (*S. aureus* ATCC 6538) as well as two Gram-negative bacteria: *Klebsiella pneumoniae* (*K. pneumoniae* ATCC 13883) and *Pseudomonas aeruginosa* (*P. aeruginosa* ATCC 27953) in addition to the opportunistic fungus *Candida albicans* (*C. albicans* ATCC 10231) using agar well diffusion method. The antibacterial ciprofloxacin and antifungal fluconazole were used as reference drugs and the diameters of the inhibition zones (in mm) are illustrated in Table 1. The tested compounds showed variable antimicrobial activity according to the secondary amines substitution on the C-2 of the quinazolinone ring. Nearly all of the pyrrolidine derivatives **15**–**20** exhibited broad spectrum antimicrobial potential against both Gram ( +) and Gram (-) bacteria except the *para-*tolyl derivative **17** that only showed antibacterial activity against the Gram (-) pathogen *P. aeruginosa.* Also, within the same group, compounds **15**, **16** and **18** displayed promising antifungal effect against the opportunistic yeast *C. albicans*. In the diethylamino series **9**–**14,** only the unsubstituted phenyl derivative **9** exhibited antibacterial effect against both Gram ( +) and Gram (-) bacteria but it lacked antifungal activity. Meanwhile, the trimethoxy phenyl derivative **13** had antifungal effect but it was selective towards Gram (-) bacteria rather than Gram ( +) ones. Interestingly, the Gram ( +)*S. aureus* was resistant to all the members of the diethylamino series **9**–**14**. Regarding the piperidine analogues **21**–**26**, most of the derivatives displayed antibacterial effect towards the Gram (-) *K. pneumoniae* except the *para*-chloro phenyl and *para-*tolyl derivatives **22** and **23**. In this group, both the unsubstituted phenyl derivative **21** and the *para*-nitro phenyl congener **26** affected the growth of Gram ( +) and Gram (-) bacteria. Meanwhile, the mono methoxy phenyl and trimethoxy phenyl derivatives **24** and **25** were active towards the two examined Gram (-) bacteria *K. pneumoniae* and *P. aeruginosa* rather than Gram ( +) bacteria. In this series, only the *para-*tolyl derivative **23** showed antifungal activity against *C. albicans.* Similar to the diethylamino series, the piperidine derivatives did not show antibacterial effect against *S. aureus.* Concerning the morpholine analogues **27**–**32**, the *para-*tolyl derivative **29** revealed the broadest spectrum of activity within this series. However, complete loss of activity was observed upon introducing the trimethoxy phenyl moiety in compound **31**.

**Table 1:** Antimicrobial activity of compounds **9**–**32** expressed as diameters of inhibition zone (mm)

Compound	Gram positive bacteria			Gram negative bacteria		Fungi
Number	*S. aureus* ATCC 6538	*B. subtilis* ATCC 6633	*B. cereus* ATCC 6629	*K. pneumoniae* ATCC 13883	*P. aeruginosa* ATCC 27953	*C. albicans* ATCC 10231
**9**	–	15 ± 0.25	14 ± 0.09	12 ± 0.17	21 ± 0.36	–
**10**	–	–	–	15 ± 0.15	–	–
**11**	–	14 ± 0.16	–	–	–	–
**12**	–	–	–	–	18 ± 0.27	–
**13**	–	–	–	12 ± 0.07	20 ± 0.18	12 ± 0.28
**14**	–	–	–	–	–	12 ± 0.37
**15**	12 ± 0.26	10 ± 0.33	–	13 ± 0.41	–	11 ± 0.11
**16**	12 ± 0.08	–	13 ± 0.14	–	17 ± 0.26	13 ± 0.29
**17**	–	–	–	–	16 ± 0.22	–
**18**	14 ± 0.21	16 ± 0.31	17 ± 0.30	18 ± 0.20	–	13 ± 0.14
**19**	–	14 ± 0.31	17 ± 0.22	18 ± 0.24	16 ± 0.18	–
**20**	13 ± 0.11	10 ± 0.12	16 ± 0.07	16 ± 0.09	21 ± 0.17	–
**21**	–	17 ± 0.14	12 ± 0.16	12 ± 0.15	–	–
**22**	–	10 ± 0.07	–	–	–	–
**23**	–	–	–	–	–	11 ± 0.13
**24**	–	–	–	12 ± 0.14	14 ± 0.21	–
**25**	–	–	–	14 ± 0.17	21 ± 0.17	–
**26**	–	12 ± 0.11	–	11 ± 0.07	–	–
**27**	–	14 ± 0.15	–	14 ± 0.16	–	13 ± 0.09
**28**	–	12 ± 0.20	–	16 ± 0.18	–	–
**29**	–	14 ± 0.06	–	20 ± 0.15	14 ± 0.16	13 ± 0.07
**30**	12 ± 0.12	–	–	14 ± 0.15	–	–
**31**	–	–	–	–	–	–
**32**	–	14 ± 0.27	–	–	18 ± 0.09	–
Ciprofloxacin	23 ± 0.15	33 ± 0.20	29 ± 0.18	26 ± 0.17	35 ± 0.28	–
Fluconazole	–	–	–	–	–	20 ± 0.18

Concurrently, all the tested compounds displayed poor antifungal activity against *C. albicans* where the pyrrolidine and morpholine derivatives **15** and **29** inhibited its growth recording MIC=5 mg/ml.

### Structure activity relationship

Based on the preliminary antibacterial assay, compounds that displayed wide spectrum antimicrobial activity were subjected to minimum inhibitory concentration (MIC) determination (Table 2). It was found that the pyrrolidine derivative **20,** substituted with 4-nitro phenyl group, was the most active compound on most of the tested bacteria.

**Table 2:** MIC values (mg/ml) of broad spectrum compounds

Compound number	*S. aureus* ATCC 6538	*B. subtilis* ATCC 6633	*B. cereus* ATCC 6629	*K. pneumoniae* ATCC 13883	*P. aeruginosa* ATCC 27953	*C. albicans* ATCC 10231
**9**	–	2 ± 0	1 ± 0	1.5 ± 0.06	2 ± 0.29	–
**15**	2 ± 0.29	2 ± 0.29	–	2 ± 0	–	5 ± 0.29
**16**	0.5 ± 0	–	2 ± 0.14	–	1.5 ± 0	10 ± 0.5
**18**	2.5 ± 0	1.5 ± 0.29	2.5 ± 0.29	2 ± 0	–	10 ± 0.29
**19**	–	1 ± 0.29	10 ± 0.5	1 ± 0.29	0.15 ± 0.06	–
**20**	15 ± 0.5	0.5 ± 0	0.25 ± 0	0.5 ± 0.29	0.25 ± 0.14	–
**29**	–	0.5 ± 0.06	–	2.5 ± 0.5	1.5 ± 0	5 ± 0.29

Analysis of the obtained results revealed that the pyrrolidine derivative **16** bearing *para*-chloro phenyl moiety was the most active compound against *S. aureus* with MIC=0.5 mg/ml. The antibacterial effect against the same organism decreased 4 and fivefold in the unsubstituted and *para*-methoxy phenyl congeners **15** and **18**, respectively. Also, the activity dropped severely (up to 30 fold) in presence of the strong electron withdrawing group (-NO_2_) in compound **20**. On the contrary, compound **20** as well as the morpholine analogue, that bears *para-*tolyl moiety, **29** are the most active against *B. subtilis* with equal MIC=0.5 mg/ml. Meanwhile, omitting the substitution on the phenyl ring in compound **15** or introducing electron donating group (-OCH_3_), in compounds **18** and **19**, led to a decline in the activity towards *B. subtilis* by 4, 3 and twofold, respectively. Regarding the other *Bacillus* species, *B. cereus*, compound **20** also possessed the highest activity with MIC=0.25 mg/ml. Within the pyrrolidine series, the inhibitory activity towards the previous pathogen declined by replacing the nitro group with moderate electron withdrawing group (-Cl) as in compound **16** or electron donating group (-OCH_3_) as in compound **18** by 8 and tenfold, respectively, as compared to compound **20**. It was observed that the trimethoxy phenyl derivative **19** was less potent against *B. cereus* than its mono methoxy phenyl analogue by fourfold. In addition, compound **9,** which bears diethylamino moiety at C-2 of the quinazolinone ring, antagonized the growth of this bacterium with MIC=1 mg/ml.

With respect to Gram (-) bacteria, the pyrrolidine derivative bearing nitro group (compound **20)** was also the most powerful one against *K. pneumoniae* with MIC=0.5 mg/ml. The activity thereof decreased fourfold by removing the substitution on phenyl moiety, as in compound **15**, and in presence of electron donating group (-OCH_3_), as in compound **18**. Interestingly, the presence of trimethoxy substitution on the phenyl ring (compound **19**) enhanced the activity against *K. pneumoniae* by two fold compared to the mono methoxy derivative compound **18**. Compound **29** which is substituted at C-2 of quinazolinone with morpholine ring and possesses *para-*tolyl moiety as a component of Schiff’s base was the least potent member towards *K. pneumoniae* (MIC=2.5 mg/ml). Concerning *P. aeruginosa*, it was found that the pyrrolidine derivative possessing trimethoxy phenyl group **19** displayed the best MIC=0.15 mg/ml among all the tested compounds. The *p*- nitro phenyl congener **20** was slightly less active than its trimethoxy counterpart*.* Meanwhile, the antibacterial activity of compound **19** dropped tenfold upon replacing the trimethoxy electron donating group by electron withdrawing group (-Cl) in compound **16**. The least active compound against *P. aeruginosa* was the diethylamino derivative **9** with MIC=2 mg/ml.

### Cell viability assay

Cell viability testing at 5 mg/ml was carried out by colorimetric MTT assay on human normal cell line BJ1 (skin fibroblasts) to investigate the toxicity of the most broad spectrum derivatives **9**, **15**, **16**, **18**, **19**, **20** and **29** (Table 3). The viability of the cells treated with the aforementioned derivatives was more than 87% at 5 mg/ml concentration. This gives indication that the compounds are tolerated and nontoxic for human cells, as the cytotoxic dose was higher than the therapeutic dose on most of microorganisms. It is worth mentioning that the safe limit of the percent of dead cells is not an absolute value and could be more than 10% as reported in previous studies [47, 48].

**Table 3:** Cell viability assay of the potential antimicrobial candidates

Compound	Cell viability (%)
**9**	91.51 ± 2.21
**15**	88.42 ± 0.91
**16**	93.51 ± 1.54
**18**	87.18 ± 2.34
**19**	92.34 ± 3.37
**20**	88.12 ± 1.81
**29**	87.58 ± 3.61

### Biofilm inhibition assay

**Fig. 5:**
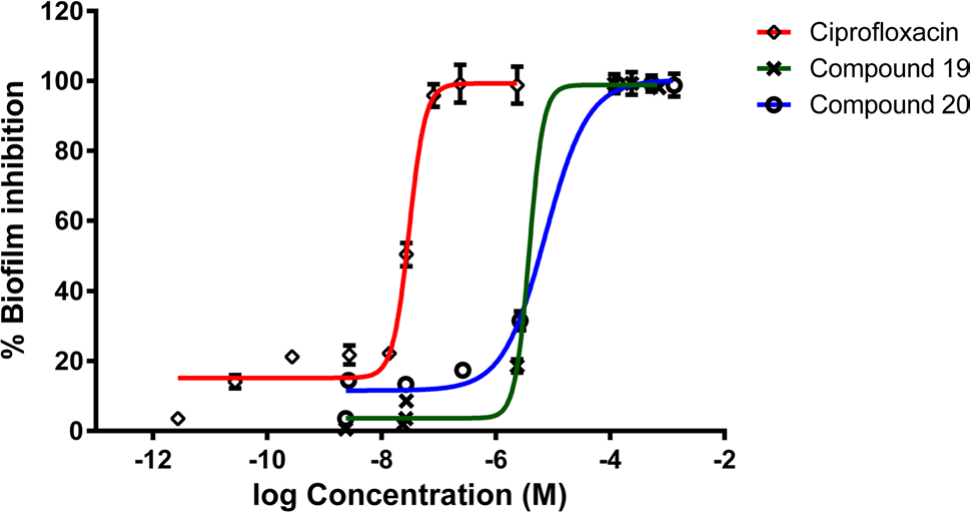
Biofilm inhibition assay results depicting the effect of compounds **19** and **20** on the aggregation of Gram (-) *P. aeruginosa* ATCC 27953, in comparison to ciprofloxacin as positive control

Structured bacterial communities known as biofilms are assemblages of single type or assortment of microorganisms that are linked to one another or to solid surfaces and are submerged in a self-produced, hydrated polymeric matrix that contains polysaccharides, proteins and DNA [49]. Compared to free-floating or planktonic cells, biofilm-dwelling bacteria have higher adaptive resistance to antibiotics and host immune defenses, which is largely responsible for their success in causing chronic infections in humans in community-acquired and nosocomial infections especially when they occupy medical implants e.g.: stents, heart valves and catheters. This resistance manifests as a 100–1000 fold rise in the minimum inhibitory and/or bactericidal concentrations which can be attributed to a number of factors, including: i) the components of the biofilm as lipids, proteins, polysaccharides and e-DNA act as a physical barrier that prevent antibiotic penetration to biofilm cells [50], ii) the fluctuation of oxygen concentrations according to the composition of biofilm, where anaerobic conditions could arise because the oxygen content is lower in the center and relatively high near the top, iii) the metabolic activity of the cells in the biofilm is a function of depth where the more deep areas inside the biofilm are deprived of nutrients and bacteria enter a latent state and become less sensitive to antibiotics because most antibiotics target either DNA replication, cell walls, or protein synthesis, which are either entirely absent from the biofilm-grown bacterial population [51] iv) antibiotic-degrading enzymes, low-affinity antibiotic targets and overexpression of efflux pumps for a range of substrates can all be produced concurrently by bacterial cells in biofilms. Due to presence of more conjugation in the biofilm state, mutation and horizontal gene transfer rates are much higher than in the free living state [52, 53]. Therefore, it is crucial to investigate novel compounds for their capacity to target and eradicate microbial biofilms to boost the antibiotics database, enable more rational use of antibiotics and/or biocides, overcome the high rate of microbial evolution and emergence of resistant strains, and evade the added cost and morbidity ensued by resistant strains infection [54, 55]. The example of *P. aeruginosa*’s biofilm has been chosen as it has been abovementioned to be fatal especially in the case of nosocomial infection of immunocompromised patients (e.g.: surgical wounds, pneumonia, blood sepsis, etc.) [54, 56]. Accordingly, compounds **19** and **20,** which are the most active against *P. aeruginosa*, were selected to evaluate their anti-biofilm activity. Results revealed that compounds **19** and **20** have biofilm inhibition effect with IC_50_ = 3.55 and 6.86 µM (1.5 and 2.59 µg/ml) (Fig. 5), respectively. It is noteworthy to point out that the value of the minimal biofilm inhibitory concentration (MBIC) is less than that of the minimum inhibitory concentration (MIC) 0.15 mg/ml for compound **19** and 0.25 mg/ml for compound **20** confirming that the inhibition of the biofilm formation is not ascribed to bacteriostatic or bactericidal effects and hence, have potential of being valuable as anti-virulence agents that do not contribute to antibiotic resistance development. These findings accord with the theories that describe how sub-MIC compromises the bacterial growth and biochemical properties including virulence factors e.g.: biofilm production, cell adhesion, cell surface hydrophobicity (CSH), production of quorum sensing signals, etc. However, it has also been documented that this behavior is strictly strain-dependent. Other studies reported higher MBIC, for the antimicrobial agents under investigation, than their MIC while the sub-MIC induced sessile bacterial aggregation in robust biofilms [56, 57]. Hence, it is crucial to determine the effect of sub-MIC doses on bacterial behavior as it can lead to development of superbugs via any of the known mechanisms including, but not limited to, antibiotic degradation, neutralization, expulsion or modification of the target site [4, 56].

### Bacterial cell surface hydrophobicity (CSH) assay

As previously described, dense bacterial biofilm aggregates are a primary means for bacterial antibiotic resistance and increased pathogenicity and virulence. Biofilms also serve to protect bacteria, in their natural habitats, against harsh conditions of nutrients depletion or competitors’ attack by acting as physical barrier. They are an organized network of microbial cells glued together within self-secreted slimy polysaccharide, nucleic acid and protein matrices. CSH stabilizes cellular adhesion in addition to other virulence or metabolic factors that act in favor of intact biofilm clumping and aggregated community protection and propagation. In case of *P. aeruginosa*, these elements include: (i) pyocyanin (N-methyl-1-hydroxyphenazine) secretion that consequently leads to extracellular DNA (eDNA) liberation, an essential component in biofilm establishment, (ii) extracellular proteases that also result in cell lysis and eDNA, (iii) intercellular quorum sensing signals, homoserine lactones or quinolones, that facilitate cell-to-cell communication, (iv) *Pseudomonas* cells undergo a phenotypic decrease in size giving rise to small colony variants (SCV) which are metabolically dormant except for up-regulated biofilm formation genes that allow for increased surface hydrophobicity, (v) hemolysin toxin, as evident by knockout mutants lacking the biofilm formation propensity, and (vi) selective attachment between surface appendages like capsules, pili and S-layer [54, 58–60].

**Fig. 6:**
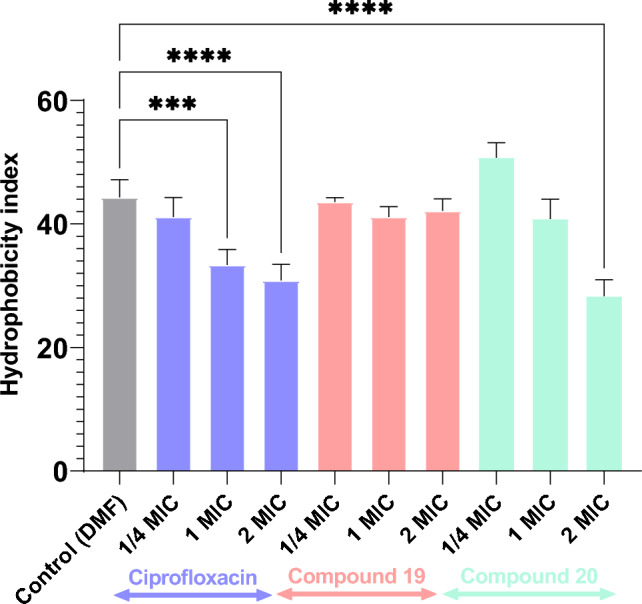
Hydrophobicity index of *Pseudomonas aeruginosa* ATCC 27953 as affected by different concentrations of compounds **19**, **20**. One way ANOVA test (with Tukey’s correction for multiple comparisons) shows significant difference between control and each test compound. Mean of 3 independent experiments ± SD triplicates, the asterisk indicates the significance of P-value < 0.0001

As CSH has been validated as a key factor in biofilm integrity and robustness, *P. aeruginosa* was cultured in presence and absence of different concentrations of the quinazolinone derivatives **19**, **20**. As depicted in Fig. 6, it is clear that the two compounds did not behave alike in this regard. While increasing concentrations of compound **20** resulted in reduced hydrophobicity index (ie: decreased CSH as indicated by less *Pseudomonas* cells in the toluene fraction) in a very similar pattern to that of ciprofloxacin, compound **19**, on the other side, had no evident effect on CSH. Despite lacking statistical significance, the slight increase in surface hydrophobicity conferred by subinhibitory concentration of compound **20** might be a demonstration of the cells’ resilience and/or persistence under stress conditions, and how they adapt for maintenance through the action of RpoS sigma factor. This usually takes place via secreting hydrophobins and membrane vesicles to promote tighter cell attachment, a phenomenon that has been reported before by other biofilm-forming *Pseudomonas*, *Staphylococcus*, *Streptococcus* and *Listeria* strains [59–62]. On the other side, higher concentrations of compound **20**, like the fluoroquinolone ciprofloxacin, increased the charge over the outer cell membrane to induce cell repulsion and reduce cell-to-cell and cell-to-surface accumulation.

### Inhibition of exopolysaccharide (EPS) production

Exopolysaccharide is one of the viscous matrix components that binds the organized biofilm structure together, making it impervious to host defenses, disinfectants and antimicrobial agents. It is essential in the initial phase of cellular attachment, together with other topographic and secretory predispositions. Since this stage is reversible, preventing or even loosening the cells’ population assembly can reduce the cost and burden of eradicating the mature and stable biofilm [60, 63]. Like other virulence determinants, the matrixome production, including EPS, is governed by QS autoinducers among the bacterial community. Relevantly, both mucoid and non-mucoid *P. aeruginosa* secrete at least one of the polysaccharides alginate, Pel and Psl helping the opportunist producers to thrive under versatile environmental conditions as they serve as a structural scaffold and a protective shield [6–8].

Both compounds **19**, **20** show the same pattern of dose-dependent EPS secretion inhibition, similar to ciprofloxacin’s, regardless of the different intensities (Table 4). A reduction in EPS production up to ~ 60% was recorded for both of the investigated compounds, for double the MIC dose, surpassing that of ciprofloxacin which was ~ 16%. Furthermore, it is pivotal to highlight the fact that compound **19** marked > 50% EPS reduction at only 1/4 of the MIC ie: it interferes with the biofilm community without being lethal to the bacteria. Hence, it can be considered as a potential anti-virulence agent to be used in combination with other antibiotics to decrease the possible tendency for induced resistance.

**Table 4:** Reduction of exopolysaccharide (EPS) production from *Pseudomonas aeruginosa* ATCC 27953 via different test compounds concentrations

Compound	Concentration	% EPS inhibition
Ciprofloxacin	1/4 MIC	1.46 ± 0.28
	1 MIC	8.98 ± 0.52
	2 MIC	16.54 ± 0.61
**19**	1/4 MIC	54.15 ± 4.00
	1 MIC	55.93 ± 3.31
	2 MIC	63.79 ± 2.70
**20**	1/4 MIC	21.51 ± 3.26
	1 MIC	47.84 ± 3.00
	2 MIC	61.50 ± 2.83

The data shown are the means ± standard errors

### Twitching motility assay

**Fig. 7:**
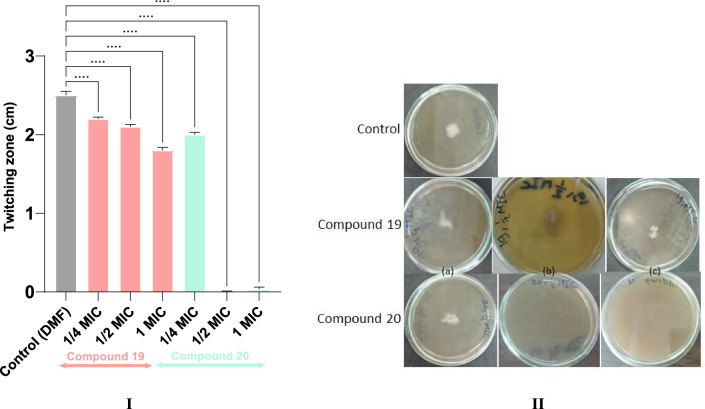
Twitching motility reduction of *Pseudomonas aeruginosa* ATCC 27953 via different concentrations of compounds **19**, **20**. (I) Comparison of twitching motility zones in comparison to the negative control (DMF). One way ANOVA test (with Tukey’s correction for multiple comparisons) shows significant difference between control and test compounds. Mean of 3 independent experiments ± SD triplicates, the asterisk indicates the significance of P-value < 0.0001. (II) Illustration of motility under the influence of the addition of compounds **19, 20** at (a) 1/4 concentration of MIC, (b) 1/2 concentration of MIC, and (c) MIC

Bacterial motility is an additional fundamental feature facilitating accumulation and adherence tosurfaces. Four different types of motilities were described for *P. aeruginosa* namely; sliding, swimming, swarming and twitching, which are governed through quorum sensing systems while the blocking thereof compromised the biofilms’ integrity and architecture [58, 64]. Twitching motility, in particular, is deemed responsible for the initial stages of free flowing cells settling and colonization of different substrata, and is mediated by polar monotrichous flagella and/or type-IV pili [57]. Both compounds **19**, **20** reduced the magnitude *P. aeruginosa* twitching motility (Fig. 7). Compound **20** remarkably blocked this type of motility starting from sub-MIC equal to half the MIC dose, similar to previous data from cephalosporins, clarithromycin and piperacillin/tazobactam [65]. Compound **19** could only abolish 30% of the twitching at the highest tested concentration (= MIC). Thus, motility inhibition, together with the other aforementioned virulence factors, can be a valid assessment for rhl and las QS quenching in the early stages of biofilm formation [58], whereas the significance of pqs is mainly evident in the late steps of dense biofilm maturity and maintenance due to the limited inducer permeability within the matrixome and short range of bioreporting. Its impact is witnessed through managing autolysis, rhamnolipid EPS secretion, and Fe^3+^ scavenging blocking pyoverdine and pyochelin synthesis pathways. As indicated before, bacterial growth is not necessarily influenced in the process since quorum quenching takes place entirely extracellularly [6, 31]. Intriguingly, the investigated quinazolinone derivatives share structural resemblance with the quinolone inducers of the pqs system. Thus, it could be easily assumed that the synthetic mimicking analogues perturb the QS by (i) downregulating the pqs signals biosynthesis or activation or both, or (ii) antagonizing the endogenous canonical autoinducer-receptor binding [7, 31].

### Molecular docking study

**Fig. 8:**
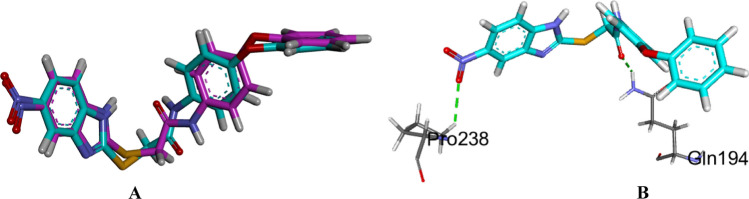
**A** Superimposition of the co-crystalized (cyan) and the docking pose (pink) of M64 in the LBD PqsR of *P. aeruginosa* with RMSD of 0.5036 Å. **B** Binding of the co-crystalized inhibitor M64 in the LBD of PqsR of *P. aeruginosa*

Biofilm formation in bacteria is a quorum sensing-controlled mechanism. Consequently, molecular docking study was performed to explain the anti-biofilm activity of compounds **19** and **20**. The binding mode of the co-crystallized inhibitor (M64) in the PqsR ligand binding domain (LBD) was initially examined. The protocol used for docking was validated by re-docking of the co-crystallized ligand (M64) in the ligand binding site with RMSD of 0.5036 Å (C-Docker interaction energy = -52.21 kcal/mol) (Fig. 8A). Also, the carbonyl group of the amide functionality of docking pose was able to reproduce the essential hydrogen bond performed by the co-crystallized ligand with the key amino acids Gln194 and Pro238 (Fig. 8B). The validation step outputs indicated the appropriateness of the used protocol for the molecular docking study of the test compounds in the LBD of PqsR.

**Fig. 9:**
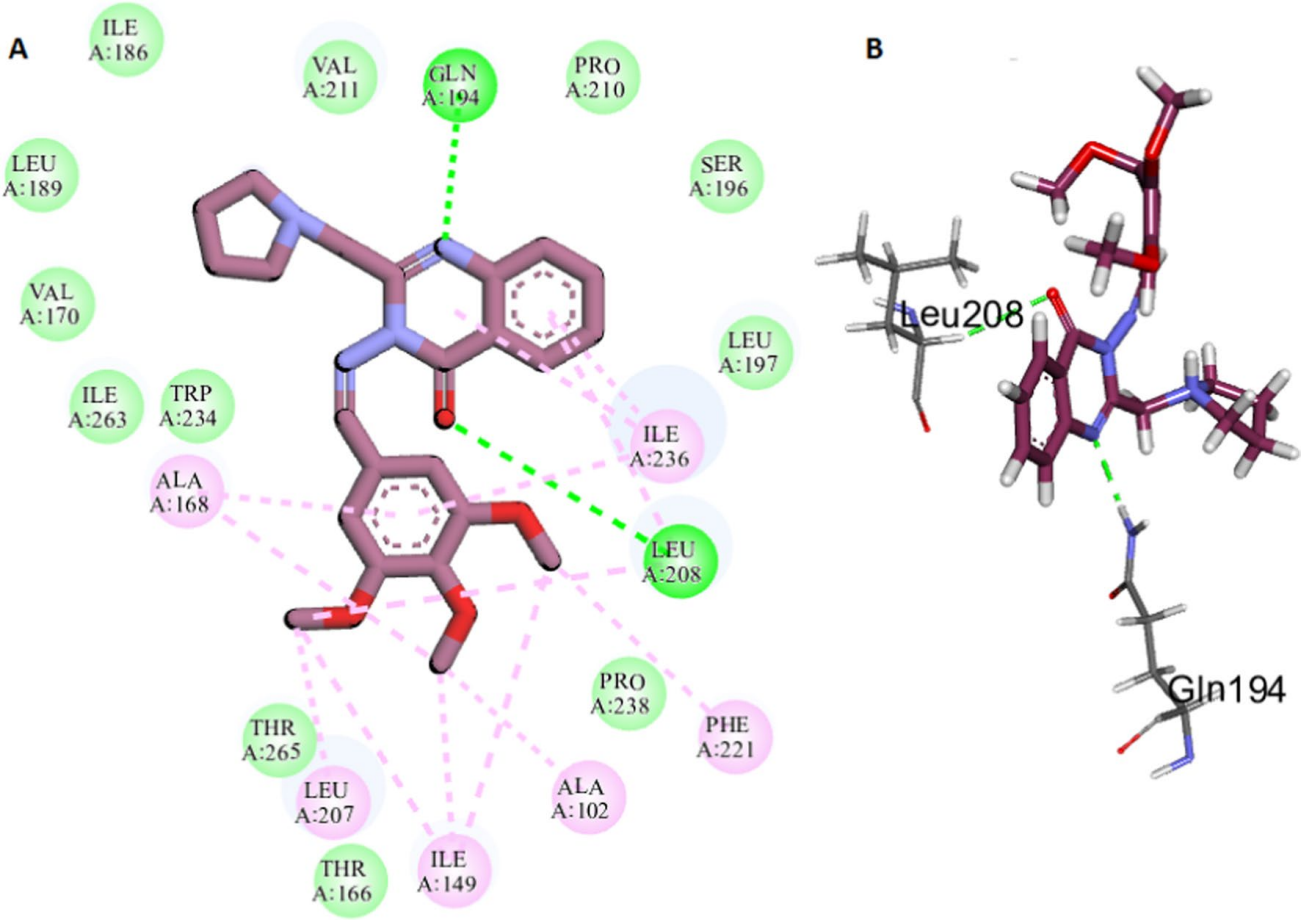
2D diagram (**A**) and 3D representation (**B**) illustrating the interactions of compound **19** within the active site of the LBD of PqsR of *P. aeruginosa*

**Fig. 10:**
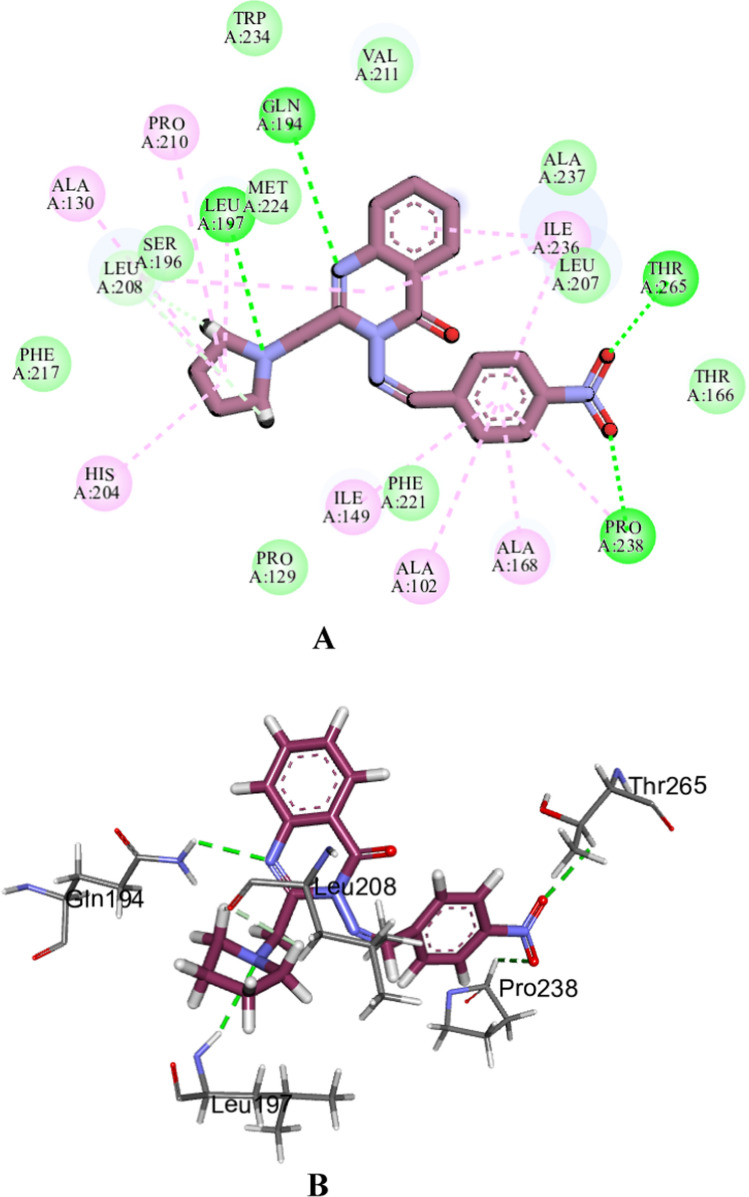
2D diagram (**A**) and 3D representation (**B**) illustrating the interactions of compound **20** within the active site of the LBD of PqsR of *P. aeruginosa*

The docking results showed that compounds **19** and **20** had C-Docker interaction energy =—49.62 kcal/mol and – 47.61 kcal/mol, respectively that are near to M64. In compound **19** the N-1 of the quinazolinone ring acts as a hydrogen bond acceptor where it forms a hydrogen bond with key amino acid Gln194. In addition the carbonyl group makes a hydrogen bond with Leu208. Moreover, it is further stabilized through hydrophobic interactions with the hydrophobic side chains of the amino acids Ala102, lle149, Ala168, Phe221, Leu207 and Leu208 and lle236 (Fig. 9). These hydrophobic residues are also important in the formation of the hydrophobic pocket which mediates PqsR interaction with other quinazolinone inhibitors [31]. Compound **20** successfully forms four hydrogen bonds with the LBD of PqsR. The nitrogen at position 1 of quinazolinone makes the essential hydrogen bond with Gln194 side chain, while he nitrogen of the pyrrolidine ring forms a hydrogen bond with amino acid Leu197 backbone. In addition, the *p*-nitro phenyl ring exhibits two hydrogen bonds with Pro238 and Thr265 side chains. Worth mentioning that performing a hydrogen bond with Thr265 residue was also reported for other PqsR inhibitors [31, 66]. The *p*-nitro phenyl moiety is directed towards a hydrophobic pocket bordered by Ala102, lle149, Ala168 and lle236 residues. Also, the complex is further stabilized through hydrophobic interactions between the prrolidinyl moiety and the side chain of Ala130, His204 and Pro210 amino acids (Fig. 10). The results of good interactions of compounds **19** and **20** with the LBD of PqsR indicate that these compounds reduce biofilm formation through their antagonistic activity on of PqsR.

## Conclusion

Bacterial resistance to antimicrobial agents has been potentiated over the last years due to their overuse, wrong prescribing, misuse in agriculture and livestock breeding, and refraining of pharmaceutical companies from investing in new antibiotic entities on account of financial and regulatory reasons. On this basis, this study aimed to design and synthesize novel quinazolin-4-ones because of the history of this class members as efficient antimicrobial hits. Compounds with broad spectrum were validated for their safety against human cell line, which promotes their appropriateness for clinical use. Further driven by the struggle against microbial resistance phenomenon, the most promising candidates **19** and **20** were selected for biofilm fighting evaluation which indicates the quorum sensing interruption. Both compounds were able to inhibit biofilm formation at concentrations below their MIC and hence, decreasing the probability of resistance development. The activities against other virulence factors (twitching motility, cell surface hydrophobicity reduction, exopolysaccharide production) were also correlated in an attempt to envisage the biofilm digestion mechanism. This was furthermore corroborated by molecular docking studies that illustrated the binding mode of the investigated compounds on the binding site of *Pseudomonas aeruginosa* quorum sensing transcriptional regulator PqsR*.* Finally, we anticipate to highlight that there is still room for developing new antibiotic molecules that can act without triggering resistance reaction. It is also critical to raise the scientific community and public awareness, and urge research efforts and regulatory decrees to be dedicated to coordinate efforts against this worldwide crisis.

## Experimental

### Chemical synthesis

#### General

Melting points were determined by the Electrothermal Capillary apparatus and were uncorrected. Purity of the final compounds detected by HPLC. HPLC analysis was carried out using Agilent 1100 series apparatus equipped with a quaternary pump, a vacuum de-gasser and detection method was using diode array (DAD) ultraviolet (UV) detector at 254 nm and HP Chemstation software. Column used was C18 column Zorbax ODS (4.6 × 150 mm i.d., 5 mm). IR spectra were recorded as KBr pellets with [JASCO FT/IR-6100 spectrometer] and the values were characterized in cm^−1^. ^1^H- and ^13^C NMR spectra were recorded at 400 (100) MHz Bruker Spectrometer and chemical shift values were recorded in ppm. Mass spectral data were measured with Thermo Scientific ISQ single quadrupole mass spectrometer at 70 eV ionization energy. Column chromatography was performed by using silica gel as a stationary phase.

##### Synthesis of methyl 2-(2-chloroacetamido)benzoate (3)

A mixture of 3.64 mmol of methyl anthranilate (**2**) and 7.28 mmol of triethylamine in dichloromethane was stirred at 0 °C for 15 min. Chloroacetyl chloride (4.37 mmol) in dichloromethane was added dropwise while maintaining the temperature at 0 °C. The reaction was stirred at 25 °C for 3 h, quenched with water, the organic layer was dried over anhydrous sodium sulphate and evaporated to get compound **3** as buff solid in 70% yield m.p. 98 °C (Lit. [67] m.p. 95–96 °C).

##### General procedure for synthesis of methyl 2-[2-(amino)acetamido]benzoates 4a-d

A mixture of 0.1 mmol of compound **3** and 0.1 mmol of NaI in acetone was refluxed for one hour followed by addition of 0.2 mmol of the appropriate secondary amine. The mixture was further refluxed for 3 h. After cooling to room temperature, the mixture was filtered and the filtrate was evaporated under reduced pressure. The residue was washed with water and extracted with ethyl acetate. The organic layer was dried and evaporated to get **4** in pure form.

- Methyl 2-[2-(diethylamino)acetamido]benzoate (**4a**) Yellow oil, yield 88% [37].
- Methyl 2-[2-(pyrrolidin-1-yl)acetamido]benzoate (**4b**) Could not be prepared in good yield.
- Methyl 2-[2-(piperidin-1-yl)acetamido]benzoate (**4c**) White solid, 85% yield, m.p. 94 °C (Lit. [37] m.p. 96–97 °C).
- Methyl 2-(2-morpholinoacetamido)benzoate (**4d**) White solid, 80% yield, m.p. 92 °C (Lit. [37] m.p. 90.5–93 °C).

##### Synthesis of 2-(2-chloroacetamido)benzoic acid (6)

A mixture of 3.64 mmol of anthranilic acid (**1**) and 7.28 mmol of triethylamine in dichloromethane was stirred at 0 °C for 15 min. Chloroacetyl chloride (4.37 mmol) in dichloromethane was added dropwise while maintaining the temperature at 0 °C. The reaction was stirred at 25 °C for 3 h. The formed suspension was filtrated and the residue was washed several times with distilled water followed by crystallization from ethanol to obtain compound **6** as white crystals in 75% yield m.p. 184 °C (Lit. [67] m.p. 180–182 °C). ^1^H NMR (400 MHz DMSO) δ 11.81 (s, 1H), 8.52 (d, *J* = 8.36 Hz, 1H), 8.02 (d, *J* = 7.84 Hz, 1H), 7.63 (t, *J* = 15.6, 7.68 Hz, 1H), 7.22 (t, *J* = 15.16, 7.56 Hz, 1H), 4.44 (s, 2H). ^13^C NMR (100 MHz, DMSO) δ 169.76, 165.71, 140.32, 134.62, 131.65, 123.95, 120.30, 117.33, 43.89.

##### Synthesis of 2-(chloromethyl)-4H-benzo[d][1,3]oxazin-4-one (7)

A solution of 10 mmol of **6** in 8 ml acetic anhydride was refluxed for one hour. The mixture was evaporated under vacuum till dryness. The obtained residue was purified by column chromatography using DCM as mobile phase to achieve the benzoxzaine **7** as buff solid in 70% yield m.p. 92 °C (Lit. [44] m.p. 93–94 °C). ^1^H NMR (400 MHz DMSO) δ 8.12–8.14 (m, 1H), 7.94–7.98 (m, 1H), 7.65–7.69 (m, 2H), 4.69 (s, 2H). ^13^C NMR (100 MHz, DMSO) δ 158.92, 157.52, 145.73, 137.55, 129.92, 128.60, 127.28, 117.24, 42.47.

##### Synthesis of 3-amino-2-(chloromethyl)quinazolin-4(3*H*)-one (8)

To an ethanolic solution of 0.1 mmol benzoxazine **7** one equivalent hydrazine hydrate (98%) was added and the mixture was refluxed for 2 h. The solvent was evaporated and the residue was purified by column chromatography using a mixture of DCM:EtOAc 9:1 to obtain compound **8** as white solid in 30% yield m.p. 152 °C (Lit. [68] m.p. 156 °C). IR (KBr, cm^−1^): 3301, 3204 (NH_2_), 2983 (C=C), 1670 (C=O), 1599 (C=N). ^1^H NMR (400 MHz DMSO) δ 8.15–8.17 (m, 1H), 7.84–7.87 (m, 1H), 7.70 (d,* J* = 8.00 Hz, 1H), 7.56–7.60 (m, 1H), 5.75 (s, 2H), 4.89 (s, 2H).^13^C NMR (100 MHz, DMSO) δ 161.15, 154.10, 146.62, 134.90, 127.76, 127.70, 126.02, 122.02, 43.49. MS (EI) m/z (%): 209.03 (M^+^, 54.5).

##### General procedure for synthesis of quinazolin-4(3*H*)-ones 5a-d

**Method A**: A mixture of 0.05 mol of **4** derivatives and 30 ml of hydrazine hydrate (98%) in 120 ml n-butanol was refluxed overnight. After cooling to room temperature the solvent was evaporated and the obtained solid was crystallized from ethanol to get the cyclized 4-quinazolinones **5** in pure form.

**Method B**: Two equivalents of the appropriate secondary amine was added to a solution of 0.1 mmol of compound **8** in benzene. The mixture was refluxed overnight, cooled, quenched with distilled water, the organic layer was dried and evaporated to get **5** in pure form.

##### 3-Amino-2-[(diethylamino)methyl]quinazolin-4(3*H*)-one (5a) [37] Prepared by methods A and B

Yellow oil. ^1^H NMR (400 MHz CDCl_3_) δ 8.19 (d, *J* = 7.6 Hz 1H), 7.61–7.68 (m, 2H), 7.39–7.43 (m, 1H), 6.51 (br s, 2H), 3.83 (s, 2H), 2.58 (q, *J* = 14.4 Hz, 4H), 0.98 (t, *J* = 14.4 Hz, 6H). ^13^C NMR (101 MHz, CDCl_3_) δ 158.83, 151.14, 146.23, 133.65, 127.36, 126.60, 126.32, 119.94, 57.71, 47.09, 11.41. MS (EI) m/z (%): 247.11 (M^+^, 9.2).

##### 3-Amino-2-(pyrrolidin-1-ylmethyl)quinazolin-4(3*H*)-one (5b) Prepared by method B only

White solid, m.p. 100 °C. ^1^H NMR (400 MHz, CDCl_3_) δ 8.26–8.28 (m, 1H), 7.69 – 7.75 (m, 2H), 7.45 – 7.49 (m, 1H), 6.41 (br s, 2H), 3.95 (s, 2H), 2.65 (s, 4H), 1.79 (dd, *J* = 10.2, 3.2 Hz, 4H). ^13^C NMR (101 MHz, CDCl_3_) δ 158.58, 151.28, 146.16, 133.65, 127.41, 126.61, 126.29, 119.97, 58.77, 53.64, 23.46. MS (EI) m/z (%): 245.09 (M^+^ + 1, 100).

##### 3-Amino-2-(piperidin-1-ylmethyl)quinazolin-4(3*H*)-one (5c) Prepared by methods A and B

White solid, m.p. 98 °C (Lit. [37] m.p. 101–103 °C). ^1^H NMR (400 MHz, CDCl_3_) δ 8.26 – 8.28 (m, 1H), 7.68 – 7.74 (m, 2H), 7.45 (ddd, *J* = 8.2, 6.2, 2.1 Hz, 1H), 6.54 (br s, 2H), 3.76 (s, 2H), 2.54 (br s, 4H), 1.53–1.58 (m, 4H), 1.45 (d, *J* = 5.0 Hz, 2H). ^13^C NMR (101 MHz, CDCl_3_) δ 158.76, 150.46, 146.16, 133.68, 127.27, 126.57, 126.17, 119.97, 62.40, 54.34, 25.65, 23.87.

##### 3-Amino-2-(morpholinomethyl)quinazolin-4(3*H*)-one (5d) Prepared by methods A and B

White solid, m.p. 120–122 °C (Lit. [37] m.p. 126–127 °C). ^1^H NMR (400 MHz, CDCl_3_) δ 8.24 (d, *J* = 7.88 Hz, 1H), 7.69–7.74 (m, 2H), 7.47 (d, *J* = 8 Hz, 1H), 6.21 (br s, 2H), 3.84 (s, 2H), 3.71 (s, 4H), 2.66 (s, 4H). ^13^C NMR (100 MHz, CDCl_3_) δ 158.34, 149.15, 145.09, 132.89, 126.46, 125.94, 125.37, 119.07, 65.71, 60.69, 52.36.

##### General procedure for synthesis of 3-(benzylideneamino)-2-yl-quinazolin-4(3*H*)-ones 9–32

An ethanolic equimolar solution of the primary amine derivative **5a**-**d** and the appropriate aldehyde in presence of few drops of glacial acetic acid was refluxed overnight. Ethanol was evaporated, the residue was washed with distilled water and extracted with EtOAc. The organic layer was dried and evaporated to get the final Schiff bases **9**–**32** in crude form which were purified by gradient elution column chromatography using a mixture of DCM:EtOAc:Methanol as mobile phase.

##### 3-(Benzylideneamino)-2-[(diethylamino)methyl]quinazolin-4(3*H*)-one (9)

White solid, m.p. 78 °C, yield 75%, HPLC: purity 98.34%, *R*_t_ = 3.42 min. IR (KBr, cm^−1^): 1676 (C=O), 1601 (C=N). ^1^H NMR (400 MHz, CDCl_3_) δ 8.82 (s, 1H), 8.32 (d, *J* = 8.3 Hz, 1H), 7.92 (d, *J* = 1.6 Hz, 2H), 7.82 – 7.72 (m, 2H), 7.61 – 7.46 (m, 4H), 3.86 (s, 2H), 2.68 (q, *J* = 7.1 Hz, 4H), 0.97 (t, *J* = 7.1 Hz, 6H).^13^C NMR (101 MHz, CDCl_3_) δ 168.97, 158.63, 153.47, 146.36, 134.04, 132.68, 132.46, 128.97, 128.87, 127.63, 127.12, 126.66, 121.75, 56.32, 46.95, 11.51. MS (EI) m/z (%): 335.24 (M^+^ + 1, 41.12). Anal. calcd. for C_20_H_22_N_4_O: C, 71.83; H, 6.63; N, 16.75. Found: C, 71.97; H, 6.82; N, 16.88.

##### 3-[(4-Chlorobenzylidene)amino]-2-[(diethylamino)methyl]quinazolin-4(3*H*)-one (10)

White solid, m.p. 124 °C, yield 81%, HPLC: purity 98.69%, *R*_t_ = 6.74 min. IR (KBr, cm^−1^): 1681 (C=O), 1597 (C=N). ^1^H NMR (400 MHz, CDCl_3_) δ 8.84 (s, 1H), 8.32 (d, *J* = 7.8 Hz, 1H), 7.87 (d, *J* = 8.5 Hz, 2H), 7.81 – 7.74 (m, 2H), 7.55 – 7.47 (m, 3H), 3.87 (s, 2H), 2.69 (d, *J* = 6.5 Hz, 4H), 0.97 (t, *J* = 7.1 Hz, 6H).^13^C NMR (101 MHz, CDCl_3_) δ 167.27, 158.62, 146.26, 138.62, 134.17, 131.21, 130.07, 129.29, 127.66, 127.16, 126.79, 121.69, 56.46, 46.98, 11.50. MS (EI) m/z (%): 369.23 (M^+^ + 1, 38.81). Anal. calcd. for C_20_H_21_ClN_4_O: C, 65.12; H, 5.74; N, 15.19. Found: C, 65.37; H, 5.96; N, 15.27.

##### 2-[(Diethylamino)methyl]-3-[(4-methylbenzylidene)amino]quinazolin-4(3*H*)-one (11)

White solid, m.p. 74 °C, yield 70%, HPLC: purity 98.81%, *R*_t_ = 4.72 min. IR (KBr, cm^−1^): 1681 (C=O), 1599 (C=N). ^1^H NMR (400 MHz, CDCl_3_) δ 8.74 (s, 1H), 8.32 (dd, *J* = 8.0, 0.7 Hz, 1H), 7.82 (d, *J* = 8.1 Hz, 2H), 7.75 (dd, *J* = 16.8, 1.9 Hz, 2H), 7.49 (ddd, *J* = 8.2, 6.6, 1.8 Hz, 1H), 7.32 (d, *J* = 8.0 Hz, 2H), 3.85 (s, 2H), 2.68 (q, *J* = 7.1 Hz, 4H), 2.45 (s, 3H), 0.97 (t, *J* = 7.1 Hz, 6H). ^13^C NMR (101 MHz, CDCl_3_) δ 169.18, 158.66, 153.50, 146.39, 143.18, 133.96, 129.97, 129.62, 129.00, 127.61, 127.09, 126.59, 121.77, 56.28, 46.93, 21.76, 11.51. MS (EI) m/z (%): 348.39 (M^+^, 43). Anal. calcd. for C_21_H_24_N_4_O: C, 72.39; H, 6.94; N, 16.08. Found: C, 72.55; H, 7.11; N, 16.24.

##### 2-[(Diethylamino)methyl]-3-[(4-methoxybenzylidene)amino]quinazolin-4(3*H*)-one (12)

White solid, m.p. 102–104 °C, yield 83%, HPLC: purity 98.67%, *R*_t_ = 5.06 min. IR (KBr, cm^−1^): 1675 (C=O), 1598 (C=N). ^1^H NMR (400 MHz, CDCl_3_) δ 8.66 (s, 1H), 8.32 (d, *J* = 8.6 Hz, 1H), 7.87 (d, *J* = 8.8 Hz, 2H), 7.80 – 7.72 (m, 2H), 7.52 – 7.45 (m, 1H), 7.02 (d, *J* = 8.8 Hz, 2H), 3.90 (s, 3H), 3.84 (s, 2H), 2.68 (d, *J* = 7.0 Hz, 4H), 0.97 (t, *J* = 7.1 Hz, 6H). ^13^C NMR (101 MHz, CDCl_3_) δ 168.87, 163.14, 158.73, 153.21, 146.40, 133.92, 130.87, 127.59, 127.06, 126.56, 125.26, 121.76, 114.34, 56.23, 55.46, 46.95, 11.50. MS (EI) m/z (%): 365.26 (M^+^ + 1, 61.48). Anal. calcd. for C_21_H_24_N_4_O_2_: C, 69.21; H, 6.64; N, 15.37. Found: C, 69.42; H, 6.87; N, 15.45.

##### 2-[(Diethylamino)methyl]-3-[(3,4,5-trimethoxybenzylidene)amino]quinazolin-4(3*H*)-one (13)

White solid, m.p. 116 °C, yield 70%, HPLC: purity 98.78%, *R*_t_ = 6.08 min. IR (KBr, cm^−1^): 1672 (C=O), 1601 (C=N). ^1^H NMR (400 MHz, CDCl_3_) δ 8.69 (s, 1H), 8.31 (d, *J* = 7.7 Hz, 1H), 7.80 – 7.71 (m, 2H), 7.52 – 7.45 (m, 1H), 7.17 (s, 2H), 3.96 (s, 3H), 3.93 (s, 6H), 3.86 (s, 2H), 2.70 (d, *J* = 6.9 Hz, 4H), 0.99 (t, *J* = 7.1 Hz, 6H). ^13^C NMR (101 MHz, CDCl_3_) δ 168.73, 158.66, 153.50, 146.31, 141.92, 134.06, 127.78, 127.62, 127.11, 126.70, 121.68, 106.14, 61.03, 56.25, 47.00, 11.57. MS (EI) *m/z* (%): 425.2 (M^+^  + 1, 2.74). Anal. calcd. for C_23_H_28_N_4_O_4_: C, 65.08; H, 6.65; N, 13.20. Found: C, 65.31; H, 6.73; N, 13.38.

##### 2-[(Diethylamino)methyl]-3-[(4-nitrobenzylidene)amino]quinazolin-4(3*H*)-one (14)

Yellow solid, m.p. 155 °C, yield 75%, HPLC: purity 99.25%, *R*_t_ = 6.73 min. IR (KBr, cm^−1^): 1676 (C=O), 1603 (C=N). ^1^H NMR (400 MHz, CDCl_3_) δ 9.15 (s, 1H), 8.37 (d, *J* = 8.8 Hz, 2H), 8.32 (d, *J* = 7.9 Hz, 1H), 8.10 (d, *J* = 8.8 Hz, 2H), 7.80 (d, *J* = 4.3 Hz, 2H), 7.59 – 7.47 (m, 1H), 3.92 (s, 2H), 2.71 (d, *J* = 6.7 Hz, 4H), 0.98 (t, *J* = 7.1 Hz, 6H).^13^C NMR (101 MHz, CDCl_3_) δ 164.51, 158.68, 149.93, 146.06, 138.63, 134.49, 129.48, 127.77, 127.27, 127.06, 124.09, 121.60, 47.02, 11.51. MS (EI) *m/z* (%): 380.2 (M^+^  + 1, 1.4). Anal. calcd. for C_20_H_21_N_5_O_3_: C, 63.31; H, 5.58; N, 18.46. Found: C, 63.49; H, 5.82; N, 18.83.

##### 3-(Benzylideneamino)-2-(pyrrolidin-1-ylmethyl)quinazolin-4(3*H*)-one (15)

White solid, m.p. 108–110 °C, yield 72%, HPLC: purity 97.74%, *R*_t_ = 5.28 min. IR (KBr, cm^−1^): 1677 (C=O), 1589 (C=N). ^1^H NMR (400 MHz, CDCl_3_) δ 8.98 (s, 1H), 8.32 (m, 1H), 7.92 (m, 2H), 7.81 (m, 1H), 7.76 (m, 1H), 7.52 (m, 4H), 3.97 (s, 2H), 2.77 (m, 4H), 1.82 (m, 4H).^13^C NMR (101 MHz, CDCl_3_) δ 167.76, 158.81, 152.98, 146.49, 134.21, 132.89, 132.61, 129.07, 129.00, 127.93, 127.19, 126.79, 121.80, 57.85, 54,47, 23.79. MS (EI) *m/z* (%): 332.52 (M^+^, 44.6). Anal. calcd. for C_20_H_20_N_4_O: C, 72.27; H, 6.06; N, 16.86. Found: C, 72.47; H, 6.22; N, 17.11.

##### 3-[(4-Chlorobenzylidene)amino]-2-(pyrrolidin-1-ylmethyl).quinazolin-4(3*H*)-one (16)

White solid, m.p. 138 °C, yield 79%, HPLC: purity 98.11%, *R*_t_ = 6.68 min. IR (KBr, cm^−1^): 1677 (C=O), 1589 (C=N). ^1^H NMR (400 MHz, CDCl_3_) δ 8.99 (s, 1H), 8.30 (dd, *J* = 8.0, 1.0 Hz, 1H), 7.84 (d, *J* = 8.5 Hz, 2H), 7.82 – 7.72 (m, 2H), 7.52 – 7.44 (m, 3H), 3.94 (s, 2H), 2.74 (br s, 4H), 1.80 (dt, *J* = 6.5, 3.1 Hz, 4H). ^13^C NMR (101 MHz, CDCl_3_) δ 165.86, 158.71, 152.88, 146.30, 138.58, 134.16, 131.34, 129.95, 129.33, 127.82, 127.06, 126.73, 121.62, 57.83, 54.34, 23.67. MS (EI) *m/z* (%): 366.7 (M^+^, 1.82). Anal. calcd. for C_20_H_19_ClN_4_O: C, 65.48; H, 5.22; N, 15.27. Found: C, 65.87; H, 5.52; N, 15.41.

##### 3-[(4-Methylbenzylidene)amino]-2-(pyrrolidin-1-ylmethyl)quinazolin-4(3*H*)-one (17)

White solid, m.p. 156 °C, yield 69%, HPLC: purity 98.07%, *R*_t_ = 8.21 min. IR (KBr, cm^−1^): 1681 (C=O), 1606 (C=N). ^1^H NMR (500 MHz, CDCl_3_) δ 8.84 (s, 1H), 8.29 (d, *J* = 8.0 Hz, 1H), 7.78 (d, *J* = 8.1 Hz, 3H), 7.77 – 7.70 (m, 1H), 7.46 (t, *J* = 8.0 Hz, 1H), 7.30 (d, *J* = 7.9 Hz, 2H), 3.91 (s, 2H), 2.72 (s, 3H), 2.43 (s, 4H), 1.78 (s, 4H). ^13^C NMR (126 MHz, CDCl_3_) δ 168.28, 158.43, 153.19, 146.64, 142.80, 133.74, 130.15, 129.95, 129.13, 127.93, 127.11, 126.71, 121.40, 57.86, 54.58, 23.60, 21.67. MS (EI) *m/z* (%): 347.22 (M^+^  + 1, 12.92). Anal. calcd. for C_21_H_22_N_4_O: C, 72.81; H, 6.40; N, 16.17. Found: C, 73.14; H, 6.72; N, 16.32.

##### 3-[(4-Methoxybenzylidene)amino]-2-(pyrrolidin-1-ylmethyl)quinazolin-4(3*H*)-one (18)

White solid, m.p. 118–120 °C, yield 75%, HPLC: purity 96.91%, *R*_t_ = 7.14 min. IR (KBr, cm^−1^): 1678 (C=O), 1601 (C=N). ^1^H NMR (400 MHz, CDCl_3_) δ 8.81 (s, 1H), 8.32 (dd, *J* = 8.0, 1.0 Hz, 1H), 7.88 (d, *J* = 8.8 Hz, 2H), 7.77 (ddd, *J* = 12.8, 9.7, 4.8 Hz, 2H), 7.53 – 7.46 (m, 1H), 7.03 (d, *J* = 8.8 Hz, 2H), 3.96 (s, 2H), 3.92 (s, 3H), 2.80 (s, 4H), 1.83 (s, 4H). ^13^C NMR (101 MHz, CDCl_3_) δ 167.66, 163.20, 158.73, 152.74, 146.42, 133.96, 130.78, 127.77, 127.01, 126.58, 125.31, 121.71, 114.42, 57.55, 55.49, 54.31, 23.70. MS (EI) *m/z* (%): 363.28 (M^+^  + 1, 19.49). Anal. calcd. for C_21_H_22_N_4_O_2_: C, 69.59; H, 6.12; N, 15.46. Found: C, 69.78; H, 6.36; N, 15.78.

##### 2-(Pyrrolidin-1-ylmethyl)-3-[(3,4,5-trimethoxybenzylidene)amino]quinazolin-4(3*H*)-one (19)

White solid, m.p. 166 °C, yield 80%, HPLC: purity 99.36%, *R*_t_ = 4.04 min. IR (KBr, cm^−1^): 1676 (C=O), 1600 (C=N). ^1^H NMR (400 MHz, CDCl_3_) δ 8.82 (s, 1H), 8.31 (d, *J* = 7.9 Hz, 1H), 7.77 (dt, *J* = 16.4, 4.8 Hz, 2H), 7.53 – 7.45 (m, 1H), 7.16 (s, 2H), 3.96 (s, 5H), 3.95 (s, 6H), 2.80 (br s, 4H), 1.83 (br s, 4H). ^13^C NMR (101 MHz, CDCl_3_) δ 167.81, 158.68, 153.57, 152.59, 146.31, 142.01, 134.14, 127.82, 127.75, 127.06, 126.75, 121.61, 106.07, 61.04, 57.61, 56.28, 54.31, 23.70. MS (EI) *m/z* (%): 423.33 (M^+^  + 1, 3.12). Anal. calcd. for C_23_H_26_N_4_O_4_: C, 65.39; H, 6.20; N, 13.26. Found: C, 65.52; H, 6.46; N, 13.49.

##### 3-[(4-Nitrobenzylidene)amino]-2-(pyrrolidin-1-ylmethyl)quinazolin-4(3*H*)-one (20)

Yellow solid, m.p. 156 °C, yield 70%, HPLC: purity 98.96%, *R*_t_ = 6.29 min. IR (KBr, cm^−1^): 1676 (C=O), 1616 (C=N). ^1^H NMR (400 MHz, CDCl_3_) δ 9.27 (s, 1H), 8.29 (d, *J* = 8.8 Hz, 2H), 8.23 (d, *J* = 7.7 Hz, 1H), 7.99 (d, *J* = 8.8 Hz, 2H), 7.71 (q, *J* = 8.1 Hz, 2H), 7.46 – 7.38 (m, 1H), 3.91 (s, 2H), 2.68 (s, 4H), 1.73 (s, 4H). ^13^C NMR (100 MHz, CDCl_3_) δ 162.74, 158.98, 153.03, 149.85, 146.10, 138.90, 134.53, 129.36, 127.93, 127.20, 127.03, 124.16, 121.58, 58.09, 54.42, 23.68. MS (EI) *m/z* (%): 377.34 (M^+^, 7.5). Anal. calcd. for C_20_H_19_N_5_O_3_: C, 63.65; H, 5.07; N, 18.56. Found: C, 63.91; H, 5.32; N, 18.89.

##### 3-(Benzylideneamino)-2-(piperidin-1-ylmethyl)quinazolin-4(3*H*)-one (21)

White solid, m.p. 98 °C, yield 75%, HPLC: purity 97.36%, R_t_ = 6.47 min. IR (KBr, cm^−1^): 1678 (C=O), 1601 (C=N). ^1^H NMR (400 MHz, CDCl_3_) δ 8.50 (s, 1H), 8.30 (s, 1H), 7.91 (d, *J* = 8.5 Hz, 2H), 7.41 – 7.56 (m, 6H), 3.08 (s, 2H), 2.51 (br s, 4H), 1.32 (d, *J* = 12 Hz, 6H). ^13^C NMR (101 MHz, CDCl_3_) δ 164.68, 158.65, 149.03, 137.67, 134.22, 132.65, 131.84, 130.45, 128.66, 127.65, 123.22, 122.52, 122.02, 63.03, 55.03, 25.60, 23.72. MS (EI) m/z (%): 346.9 (M^+^, 9.9). Anal. calcd. for C_21_H_22_N_4_O: C, 72.81; H, 6.40; N, 16.17. Found: C, 73.14; H, 6.72; N, 16.38.

##### 3-[(4-Chlorobenzylidene)amino]-2-(piperidin-1-ylmethyl)quinazolin-4(3*H*)-one (22)

White solid, m.p. 130 °C, yield 79%, HPLC: purity 95.27%, *R*_t_ = 4.43 min. IR (KBr, cm^−1^): 1675 (C=O), 1602 (C=N). ^1^H NMR (400 MHz, CDCl_3_) δ 8.83 (s, 1H), 8.32 (d, *J* = 8.4 Hz, 1H), 7.88 (d, *J* = 8.9 Hz, 2H), 7.78 (d, *J* = 8.5 Hz, 2H), 7.50 (d, *J* = 8.8 Hz, 3H), 3.71 (s, 2H), 2.56 (br s, 4H), 1.42 (d, *J* = 14.7 Hz, 6H). ^13^C NMR (101 MHz, CDCl_3_) δ 167.81, 158.56, 151.99, 146.21, 138.66, 134.14, 131.21, 130.00, 129.32, 127.67, 127.09, 126.83, 121.76, 61.13, 54.51, 25.79, 23.93. MS (EI) *m/z* (%): 380.83 (M^+^, 1.48). Anal. calcd. for C_21_H_21_ClN_4_O: C, 66.22; H, 5.56; N, 14.71. Found: C, 66.51; H, 5.77; N, 14.97.

##### 3-[(4-Methylbenzylidene)amino]-2-(piperidin-1-ylmethyl)quinazolin-4(3*H*)-one (23)

White solid, m.p. 124 °C, yield 75%, HPLC: purity 98.79%, *R*_t_ = 6.73 min. IR (KBr, cm^−1^): 1676 (C=O), 1604 (C=N). ^1^H NMR (400 MHz, CDCl_3_) δ 8.73 (s, 1H), 8.33 (d, *J* = 7.8 Hz, 1H), 7.82 (d, *J* = 8.0 Hz, 2H), 7.80 – 7.71 (m, 2H), 7.54 –7.45 (m, 1H), 7.32 (d, *J* = 7.9 Hz, 2H), 3.70 (s, 2H), 2.55 (br s, 4H), 2.45 (s, 3H), 1.41 (d, *J* = 20.9 Hz, 6H). ^13^C NMR (101 MHz, CDCl_3_) δ 169.78, 158.61, 152.03, 146.32, 143.24, 133.95, 129.94, 129.66, 128.94, 127.62, 127.04, 126.66, 121.84, 60.79, 54.51, 25.77, 23.97, 21.77. MS (EI) *m/z* (%): 361.16 (M^+^  + 1, 3). Anal. calcd. for C_22_H_24_N_4_O: C, 73.31; H, 6.71; N, 15.54. Found: C, 73.65; H, 6.89; N, 15.81.

##### 3-[(4-Methoxylbenzylidene)amino]-2-(piperidin-1-ylmethyl)quinazolin-4(3*H*)-one (24)

White solid, m.p. 138 °C, yield 84%, HPLC: purity 100%, *R*_t_ = 5.16 min. IR (KBr, cm^−1^): 1674 (C=O), 1598 (C=N). ^1^H NMR (400 MHz, CDCl_3_) δ 8.65 (s, 1H), 8.32 (d, *J* = 7.7 Hz, 1H), 7.88 (d, *J* = 8.8 Hz, 2H), 7.80 – 7.70 (m, 2H), 7.53 – 7.44 (m, 1H), 7.01 (d, *J* = 8.8 Hz, 2H), 3.90 (s, 3H), 3.68 (s, 2H), 2.54 (br s, 4H), 1.41 (d, *J* = 19.4 Hz, 6H). ^13^C NMR (101 MHz, CDCl_3_) δ 169.49, 163.18, 158.67, 152.42, 146.35, 133.90, 130.81, 127.59, 127.00, 126.67, 125.24, 121.84, 114.37, 60.95, 55.46, 54.50, 25.78, 23.97. MS (EI) *m/z* (%): 376.55 (M^+^, 6.15). Anal. calcd. for C_22_H_24_N_4_O_2_: C, 70.19; H, 6.43; N, 14.88. Found: C, 70.35; H, 6.81; N, 14.96.

##### 2-(Piperidin-1-ylmethyl)-3-[(3,4,5-trimethoxybenzylidene)amino]quinazolin-4(3*H*)-one (25)

White solid, m.p. 112–114 °C, yield 75%, HPLC: purity 97.90%, R_t_ = 3.98 min. IR (KBr, cm^−1^): 16,714 (C=O), 1602 (C=N). ^1^H NMR (400 MHz, CDCl_3_) δ 8.69 (s, 1H), 8.31 (d, *J* = 7.8 Hz, 1H), 7.76 (s, 2H), 7.54 –7.44 (m, 1H), 7.17 (s, 2H), 3.95 (s, 3H), 3.93 (s, 6H), 3.70 (s, 2H), 2.57 (s, 4H), 1.43 (d, *J* = 27.0 Hz, 6H). ^13^C NMR (101 MHz, CDCl_3_) δ 169.18, 158.64, 153.53, 146.24, 142.00, 134.06, 127.79, 127.63, 127.04, 126.77, 121.75, 61.03, 56.28, 54.56, 25.82, 23.95. MS (EI) m/z (%): 436.79 (M^+^, 10.49). Anal. calcd. for C_24_H_28_N_4_O_4_: C, 66.04; H, 6.47; N, 12.84. Found: C, 66.33; H, 6.62; N, 13.13.

##### 3-[4-Nitrobenzylidene)amino]-2-(piperidin-1-ylmethyl)quinazolin-4(3*H*)-one (26)

Yellow solid, m.p. 180–182 °C, yield 75%, HPLC: purity 100%, *R*_t_ = 4.06 min. IR (KBr, cm^−1^): 1679 (C=O), 1612 (C=N). ^1^H NMR (400 MHz, CDCl_3_) δ 9.13 (s, 1H), 8.37 (d, *J* = 8.7 Hz, 2H), 8.31 (d, *J* = 7.9 Hz, 1H), 8.10 (d, *J* = 8.7 Hz, 2H), 7.78 (d, *J* = 3.2 Hz, 2H), 7.52 (dd, *J* = 9.8, 6.5 Hz, 1H), 3.73 (s, 2H), 2.54 (s, 4H), 1.54 – 1.30 (m, 6H). ^13^C NMR (101 MHz, CDCl_3_) δ 164.78, 158.66, 152.36, 149.89, 146.06, 138.69, 134.44, 129.39, 127.77, 127.19, 127.05, 124.12, 121.67, 61.46, 54.60, 25.90, 23.95. MS (EI) m/z (%): 392.3 (M^+^  + 1, 1.3). Anal. calcd. for C_21_H_21_N_5_O_3_: C, 64.44; H, 5.41; N, 17.89. Found: C, 64.67; H, 5.58; N, 18.22.

##### 3-(Benzylideneamino)-2-(morpholinomethyl)quinazolin-4(3*H*)-one (27)

White solid, m.p. 132 °C, yield 71%, HPLC: purity 98.23%, R_t_ = 6.02 min. IR (KBr, cm^−1^): 1671 (C=O), 1594 (C=N). MS: for C_20_H_20_N_4_O_2_, calcd. 348.16, found 348.68 (M^+^). ^1^H NMR (400 MHz, CDCl_3_) δ 8.82 (s, 1H), 8.23 (d, *J* = 7.9 Hz, 1H), 7.83 (d, *J* = 7.1 Hz, 2H), 7.68 (d, *J* = 3.7 Hz, 2H), 7.53 – 7.35 (m, 4H), 3.74 (s, 2H), 3.56 (br s, 4H), 2.63 (br s, 4H). ^13^C NMR (101 MHz, CDCl_3_) δ 168.66, 158.52, 151.64, 146.05, 134.23, 132.62, 128.95, 127.68, 127.09, 121.82, 66.65, 60.30, 53.54. MS (EI) m/z (%): 348.68 (M^+^, 45). Anal. calcd. for C_20_H_20_N_4_O_2_: C, 68.95; H, 5.79; N, 16.08. Found: C, 69.28; H, 6.12; N, 16.35.

##### 3-[(4-Chlorobenzylidene)amino]-2-(morpholinomethyl)quinazolin-4(3*H*)-one (28)

White solid, m.p. 140–142 °C, yield 79%, HPLC: purity 99.35%, R_t_ = 3.73 min. IR (KBr, cm^−1^): 1677 (C=O), 1592 (C=N). ^1^H NMR (400 MHz, CDCl_3_) δ 8.93 (s, 1H), 8.32 (m, 1H), 7.86 (m, 2H), 7.78 (m, 2H), 7.51 (m, 3H), 3.81 (s, 2H) 3.64 (t, 4H), 2.69 (s, 4H). ^13^C NMR (101 MHz, CDCl_3_) δ 167.03, 158.72, 151.53, 146.14, 138.97, 134.46, 131.23, 130.06, 129.57, 127.84, 127.31, 127.21, 121.86, 66.88, 60.61, 53.72. MS (EI) m/z (%): 383.10 (M^+^  + 1, 0.96). Anal. calcd. for C_20_H_19_ ClN_4_O_2_: C, 62.75; H, 5.00; N, 14.63. Found: C, 63.12; H, 5.31; N, 14.88.

##### 3-[(4-Methylbenzylidene)amino]-2-(morpholinomethyl)quinazolin-4(3*H*)-one (29)

White solid, m.p. 128 °C, yield 71%, HPLC: purity 100%, R_t_ = 7.74 min. IR (KBr, cm^−1^): 1671 (C=O), 1604 (C=N). ^1^H NMR (400 MHz, CDCl_3_) δ 8.80 (s, 1H), 8.33 (m, 1H), 7.81 (m, 2H), 7.77 (m, 2H), 7.51 (m, 1H), 7.33 (d, *J* = 8.0 Hz, 2H), 3.79 (s, 2H) 3.63 (t, 4H), 2.68 (s, 4H), 2.46 (s, 3H). ^13^C NMR (101 MHz, CDCl_3_) δ 169.13, 158.61, 146.15, 143.51, 134.16, 129.79, 129.51, 128.91, 127.66, 127.13, 126.93, 121.82, 66.78, 60.43, 53.57. MS (EI) m/z (%): 363.27 (M^+^  + 1, 2.49). Anal. calcd. for C_21_H_22_N_4_O_2_: C, 69.59; H, 6.12; N, 15.46. Found: C, 69.81; H, 6.40; N, 15.65.

##### 3-[(4-Methoxylbenzylidene)amino]-2-(morpholinomethyl)quinazolin-4(3*H*)-one (30)

White solid, m.p. 138 °C, yield 80%, HPLC: purity 96.59%, R_t_ = 3.39 min. IR (KBr, cm^−1^): 1672 (C=O), 1604 (C=N).^1^H NMR (400 MHz, CDCl_3_) δ 8.70 (s, 1H), 8.30 (m, 1H), 7.85 (m, 2H), 7.74 (m, 2H), 7.49 (m, 1H), 7.0 (m, 2H), 3.98 (s, 3H) 3.75 (s, 2H), 3.60 (t, 4H), 2.65 (s, 4H). ^13^C NMR (101 MHz, CDCl_3_) δ 168.95, 163.42, 158.76, 146.29, 134.19, 130.91, 127.75, 127.21, 126.98, 125.20, 121.94, 114.61, 66.90, 60.55, 55.63, 53.68. MS (EI) m/z (%): 378.91 (M^+^, 3.9). Anal. calcd. for C_21_H_22_N_4_O_3_: C, 66.65; H, 5.86; N, 14.81. Found: C, 66.91; H, 6.15; N, 15.15.

##### 2-(Morpholinomethyl)-3-[(3,4,5-trimethoxybenzylidene)amino]quinazolin-4(3*H*)-one (31)

White solid, m.p. 155 °C, yield 78%, HPLC: purity 98.99%, R_t_ = 4.81 min. IR (KBr, cm^−1^): 1662 (C=O), 1575 (C=N). ^1^H NMR (400 MHz, CDCl_3_) δ 8.73 (s, 1H), 8.30 (d, *J* = 7.9 Hz, 1H), 7.75 (d, *J* = 3.5 Hz, 2H), 7.53 – 7.45 (m, 1H), 7.16 (s, 2H), 3.95 (s, 3H), 3.93 (s, 6H), 3.76 (s, 2H), 3.66 – 3.59 (m, 4H), 2.66 (br s, 4H). ^13^C NMR (101 MHz, CDCl_3_) δ 168.82, 158.54, 153.60, 151.51, 146.12, 142.09, 134.17, 127.66, 127.10, 126.93, 121.72, 106.01, 66.85, 61.04, 60.57, 56.29, 53.63. MS (EI) m/z (%): 439.29 (M^+^  + 1, 15.9). Anal. calcd. for C_23_H_26_N_4_O_5_: C, 63.00; H, 5.98; N, 12.78. Found: C, 63.31; H, 6.21; N, 13.04.

##### 2-(Morpholinomethyl)-3-[(4-nitrobenzylidene)amino]quinazolin-4(3*H*)-one (32)

Yellow solid, m.p. 200 °C, yield 68%, HPLC: purity 99.84%, R_t_ = 4.02 min. IR (KBr, cm^−1^): 1672 (C=O), 1619 (C=N). ^1^H NMR (400 MHz, CDCl_3_) δ 9.18 (s, 1H), 8.29 (d, *J* = 8.7 Hz, 3H), 7.99 (d, *J* = 8.7 Hz, 2H), 7.71 (d, *J* = 6.1 Hz, 2H), 7.45 (t, *J* = 7.0 Hz, 1H), 3.75 (s, 2H), 3.56 (s, 4H), 2.59 (s, 4H). ^13^C NMR (101 MHz, CDCl_3_) δ 163.61, 158.77, 151.76, 149.93, 145.86, 138.65, 134.61, 129.32, 127.84, 127.28, 124.22, 121.66, 66.86, 60.83, 53.72. MS (EI) m/z (%): 394.20 (M^+^  + 1, 1.93) Anal. calcd. for C_20_H_19_N_5_O_4_: C, 61.06; H, 4.87; N, 17.80. Found: C, 61.34; H, 5.14; N, 18.05.

## In vitro studies

This work has been granted the final approval of the Medical Research Ethics Committee (MREC) of the National Research Centre in Egypt after satisfying the guidelines and recommendations, under number R113010141.

### Microorganisms

The antimicrobial activity was tested against Gram positive bacteria: *Bacillus cereus* (*B. cereus* ATCC 6629), *Bacillus subtilis* (*B. subtilis* ATCC 6633), *Staphylococcus aureus* (*S. aureus* ATCC 6538), Gram negative bacteria: *Klebsiella pneumoniae* (*K. pneumoniae* ATCC 13883), *Pseudomonas aeruginosa* (*P. aeruginosa* ATCC 27953) and *Candida albicans* (*C. albicans* ATCC 10231). Glycerol stocks were thawed upon need and propagated into nutrient broth at 37 °C.

### Antimicrobial assay

The antibacterial activity of the compounds** 9**–**32** was carried out in vitro using the agar well diffusion method, originally reported by Heatley and Bauer and coworkers and described by the National Committee for Clinical Laboratory Standards (NCCLS) [69, 70]. Bacterial strains, adjusted at 10^6^ cfu/ml (equivalent to 0.5 McFarland), were used to inoculate nutrient agar plates. 9-mm diameter wells were punched into the solidified nutrient agar plates using sterile cork borer and 100 μl samples of the test compounds, dissolved in dimethyl formamide (DMF) at 20 mg/ml concentrations, were added and allowed to diffuse at 4 °C for 2 h. Subsequently, the plates were incubated overnight in an upright position at 37 °C. DMF was used as a negative control while ciprofloxacin and fluconazole (30 μg/ml) served as positive controls. After 24 h incubation, the diameters of the growth inhibition zones were recorded in mm. Data was expressed as mean ± standard deviation of three replicates.

### Determination of minimum inhibitory concentration (MIC)

Serial dilutions of the test compounds were tested to determine their MIC against the most susceptible organisms. The MIC was expressed as the lowest concentration which inhibited the visible growth of the corresponding microorganism. All assays were performed in triplicate.

## In vitro cytotoxicity

The most broad spectrum compounds were evaluated for their cytotoxic activity against BJ1 cell line using colorimetric MTT [3-(4,5-dimethylthiazol-2-yl)-2,5-diphenyltetrazolium bromide] assay [71].

### Biofilm assay

Compounds with potential activity against *P. aeruginosa* ATCC 27953 were tested for the respective biofilm inhibition. The assay was carried out in microtiter plate setup using crystal violet (CV) staining of adherent cells, as modified from [55, 64].

Overnight cultures of *P. aeruginosa* were adjusted to optical density (OD_600_) of 0.2 and sub-cultured at 37 °C in 96-well microtiter plates with serial dilutions of compounds **19** and **20**, which span three orders of magnitude above and below the corresponding MIC. After 24 h cultivation, the supernatant and planktonic cells were discarded, wells were washed with phosphate buffered saline (PBS) and biofilms were stained with 0.1% CV. CV was subsequently washed then, solubilized with 33% (v/v) glacial acetic acid. The absorbance was measured at 540 nm using SPECTROstar® Nano microplate reader (BMG LABTECH GmbH, Germany). The experiments were performed in triplicate and independently repeated three times. GraphPad Prism 6.0 software was used to estimate the IC_50_ values, which correspond to half the concentration required to obtain the maximum biofilm inhibition percentage.

### Bacterial cell surface hydrophobicity (CSH) assay

The effect of the test compounds **19** and **20** on cell surface hydrophobicity was investigated via the microbial or bacterial adherence to hydrocarbons (MATH / BATH) assay [55, 62]. Exponentially growing culture of *P. aeruginosa* ATCC 27953 incubated with or without test compounds were centrifuged, the pellets were re-suspended and OD_600_ was measured using UV/Vis spectrophotometer (Jasco V-630, Tokyo, Japan) to give *A*_*o*_. The re-suspended cells were vigorously mixed with toluene followed by measuring the OD_600_ of the aqueous phase to give *A*_*t*_. The hydrophobicity index was calculated as follows:

$$\mathrm{Hydrophobicity\, index}=\frac{{A}_{o}-{A}_{t}}{{A}_{o}}\times 100$$

### Inhibition of exopolysaccharide (EPS) production

The inhibition of exopolysaccharide production by *P. aeruginosa* ATCC 27953 using quinazolinone derivatives was assessed according to total carbohydrate measurement [63, 72].

*P. aeruginosa*’s biofilm was grown in the presence or absence of compounds (**19, 20**). Cells were incubated for 1 h with an equal volume of 5% (v/v) phenol and 5 × volume of concentrated sulfuric acid containing 0.2% (w/v) hydrazine sulphate then centrifuged at 10,000 g for 10 min (Z 326 K, HERMLE Labortechnik GmbH, Wehingen, Germany). The optical density of the supernatant was measured at 490 nm and the reduction in EPS was calculated as follows:

$$\mathrm{EPS\, quantification\, }\left(\mathrm{\%}\right)= \frac{{{\text{OD}}}_{490\, }\mathrm{of\, control}-{{\text{OD}}}_{490\, }\mathrm{of\, test}}{{{\text{OD}}}_{490}\mathrm{ of\, control}} \times 100$$

### Twitching motility assay

5 µL of overnight-grown bacteria were stab-inoculated into Luria Bertani (LB) soft or quasi-solid plates containing 0.3% agar mixed with different concentrations of compounds **19**, **20** and further incubated overnight in an upright position. The visible detectable distance of corrugated trails of twitching zones, away from the inoculation center at the petri dish-agar interface, was measured afterwards. The results were recorded as the average of several diameters’ measurements [64, 73]. DMF was used as a negative control. The experiments were performed in triplicate and represented as the mean of three measurements.

### Molecular docking

Molecular docking study was performed using discovery studio software 4.1. The 3D crystal structure of PqsR (PDB code: 6B8a), was downloaded from PDB followed by protein preparation and addition of hydrogen atoms. The binding site of the selected co-crystallized ligand (M64) was created as volumes. Before docking water molecules were omitted from the protein and the tested compounds were minimized energetically using CHARMm Force Field through ligand minimization tool. Docking was carried out using CDOCKER-CHARMm-based protocol. A maximum of 10 conformers was considered for each molecule in the docking analysis. After that the docking scores (CDOCKER interaction energy) of the best fitted conformation of each of the docked molecules with the amino acids at the LBD of PqsR were recorded.

## Supplementary Information

Below is the link to the electronic supplementary material.

Supplementary file1 (DOCX 18947 KB)
