# Supplementary material for: Design and synthesis of new quinazolinone derivatives: investigation of antimicrobial and biofilm inhibition effects
Source: Mol Divers. 2024 Apr 24;29(1):21–42. doi: 10.1007/s11030-024-10830-y (PMC11785708; doi:10.1007/s11030-024-10830-y)
Supplement: Supplementary file 1 — Supplementary file1 (DOCX 18947 KB) [file 11030_2024_10830_MOESM1_ESM.docx]

**Design and Synthesis of New Quinazolinone Derivatives: Investigation of Antimicrobial and Biofilm Inhibition Effects**

Rasha Mohamed Hassan ^1*, a^, Heba Yehia ^2, a^ , Mohammed F. El-Behairy ^3^, Aida Abdel- Sattar El-Azzouny ^1^, Mohamed Nabil Aboul-Enein ^1*^

^1^Medicinal and Pharmaceutical Chemistry Department, Pharmaceutical and Drug Industries Research Institute, National Research Centre (ID: 60014618), P.O. 12622, Dokki, Giza, Egypt.

^2^ Chemistry of Natural and Microbial Products Department, Pharmaceutical and Drug Industries Research Institute, National Research Centre (ID: 60014618), P.O. 12622, Dokki, Giza, Egypt.

^3^ Department of Organic and Medicinal Chemistry, Faculty of Pharmacy, University of Sadat City, Sadat City 32897, Egypt.

^*^ Correspondence:

Rasha Mohamed Hassan, Medicinal and Pharmaceutical Chemistry Department, Pharmaceutical and Drug Industries Research Institute, National Research Centre, P.O. 12622 Dokki, Giza, Egypt. e-mail: [rashahassan_pharma@yahoo.ca](mailto:rashahassan_pharma@yahoo.ca)

Mohamed Nabil Aboul-Enein, Medicinal and Pharmaceutical Chemistry Department, Pharmaceutical and Drug Industries Research Institute, National Research Centre, P.O. 12622 Dokki, Giza, Egypt. e-mail: [mnaboulenein@yahoo.com](mailto:mnaboulenein@yahoo.com)

^a^ Co-first authors.

**Spectral data of intermediate and target compounds**

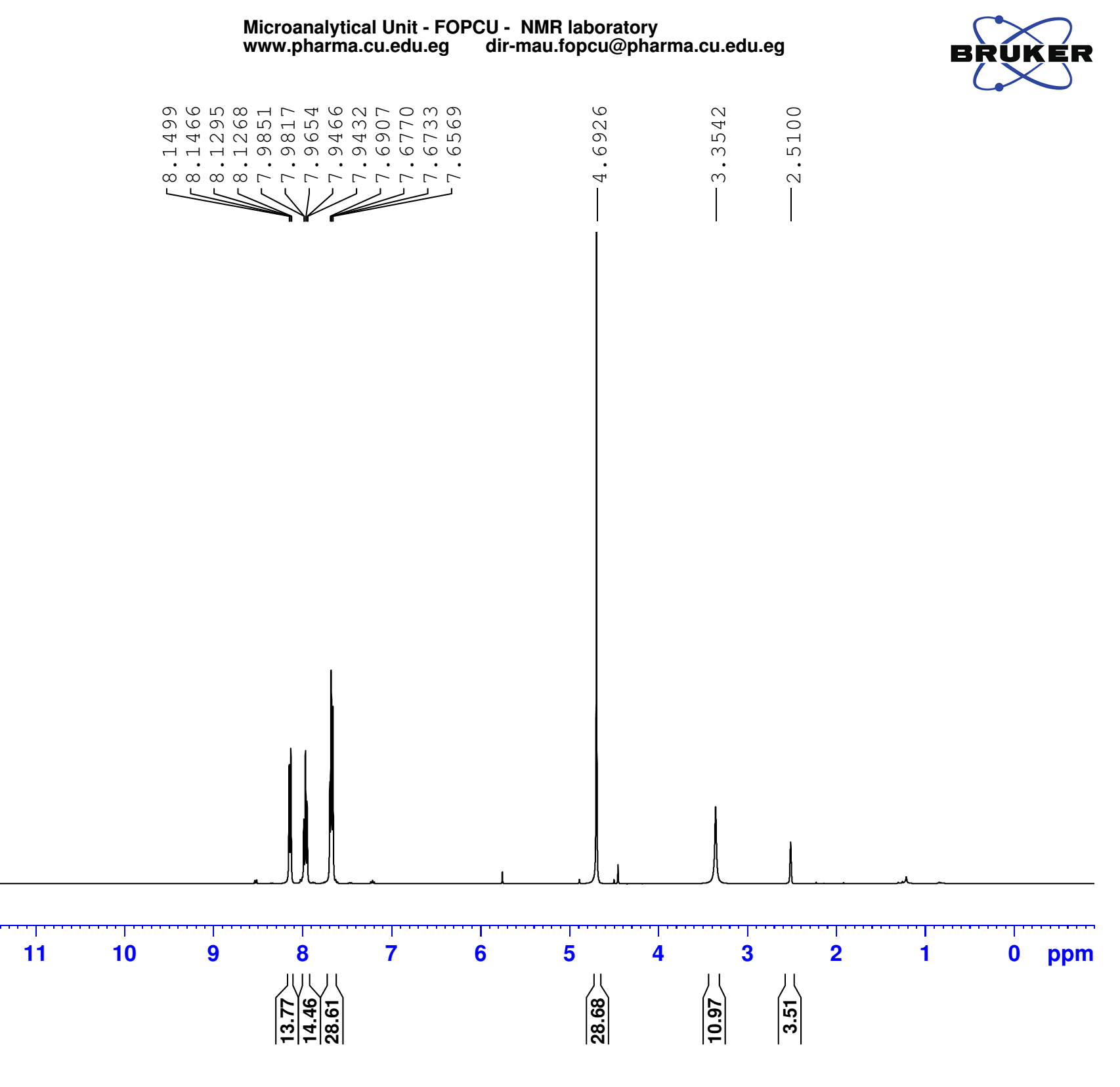


^1^H-NMR spectrum of compound **7**

**
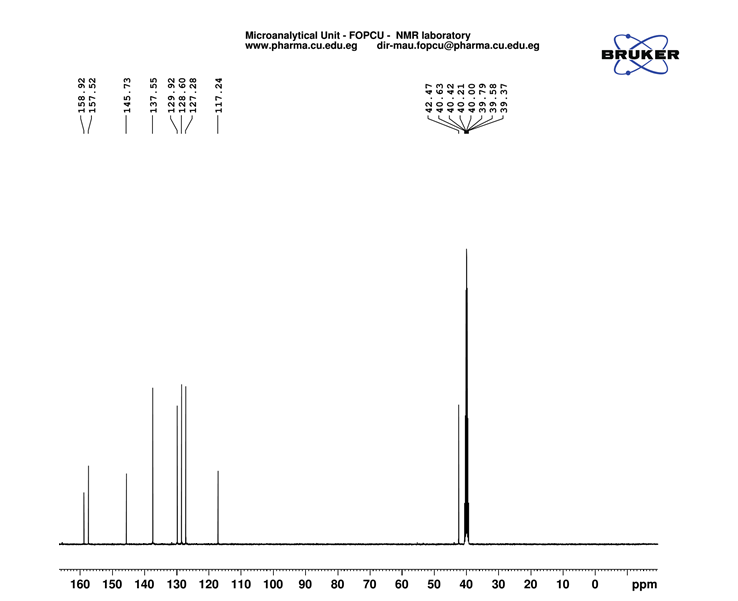
**

^13^C-NMR spectrum of compound **7**

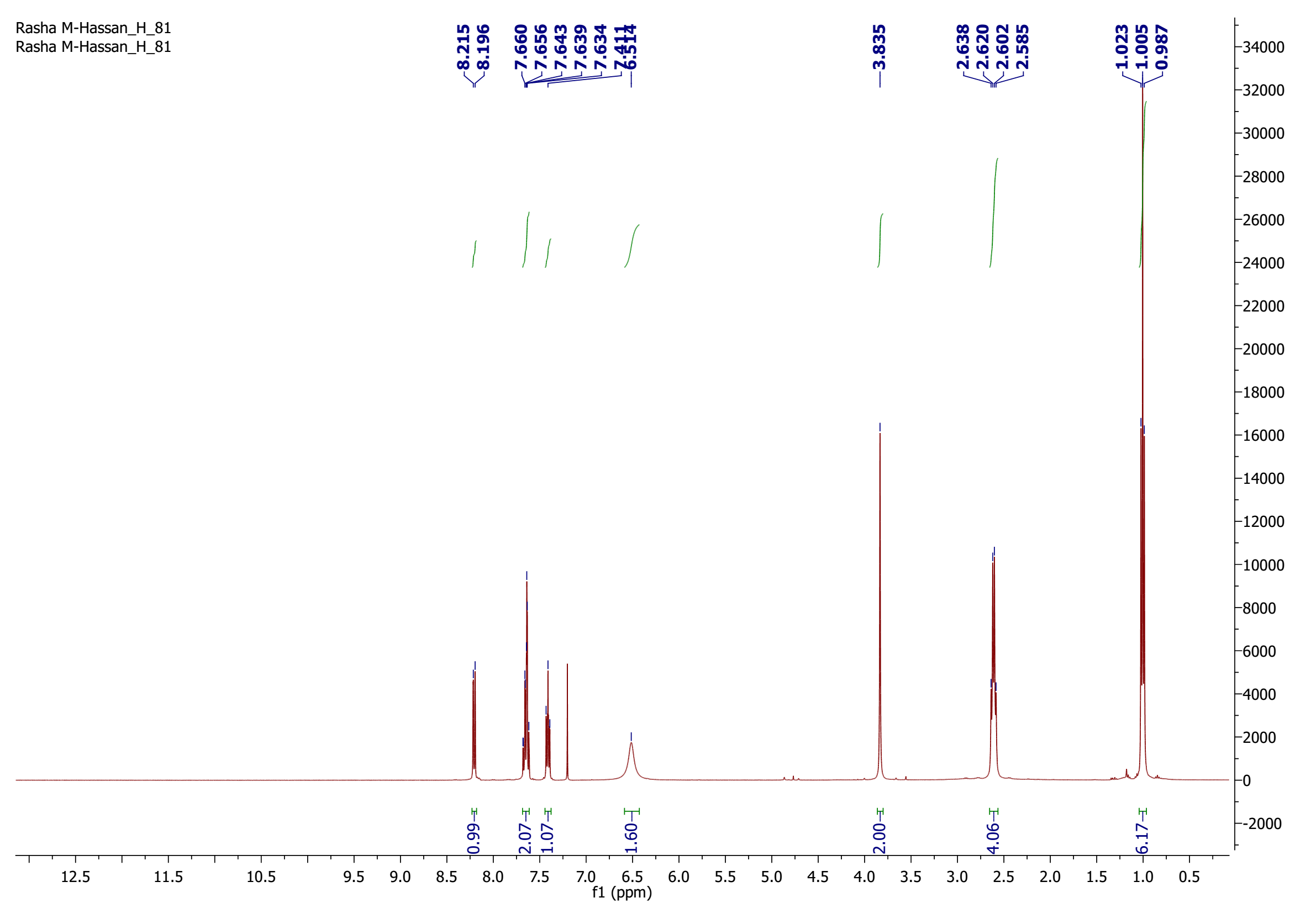


^1^H-NMR spectrum of compound **5a**


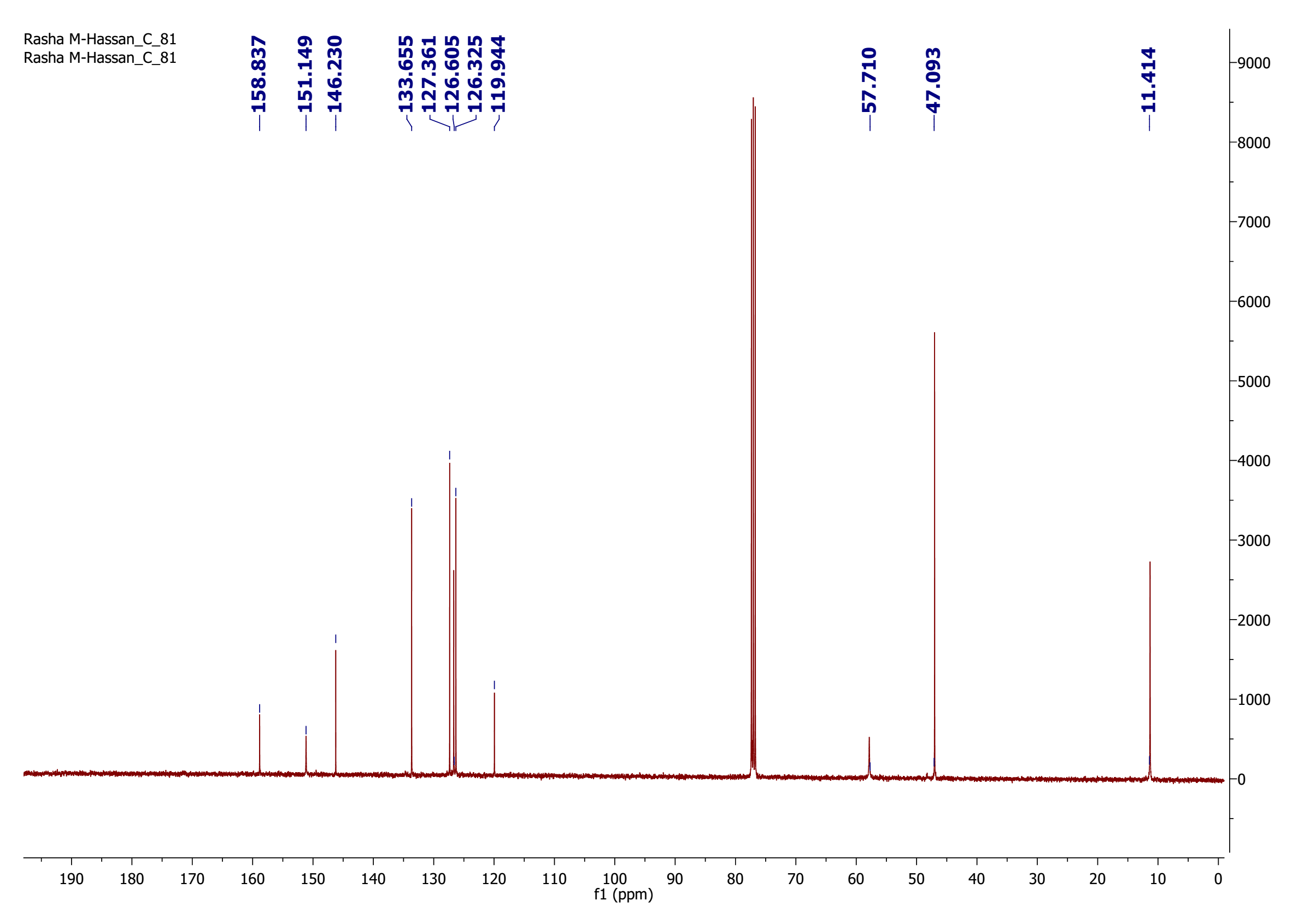


^13^C-NMR spectrum of compound **5a**

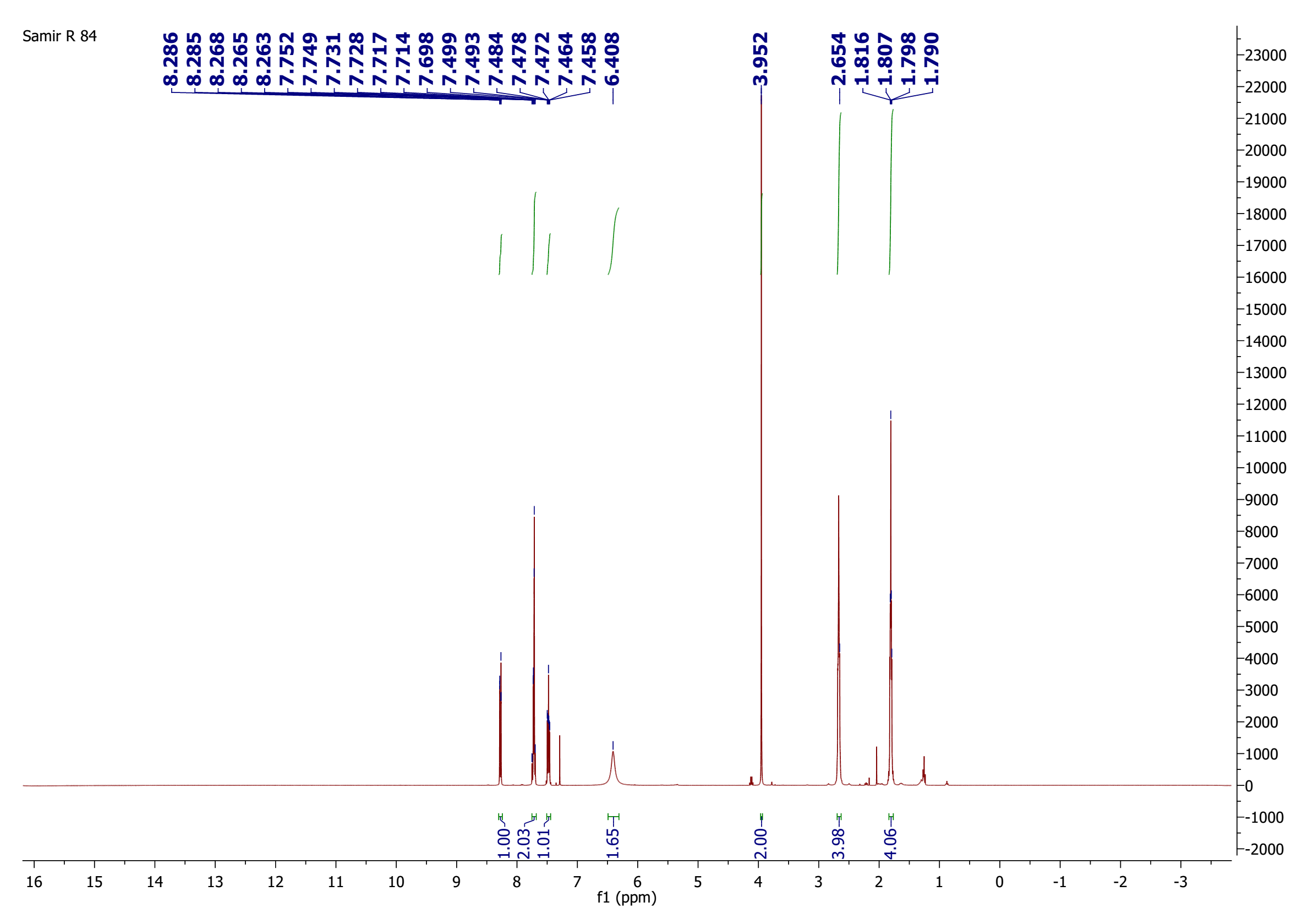


^1^H-NMR spectrum of compound **5b**


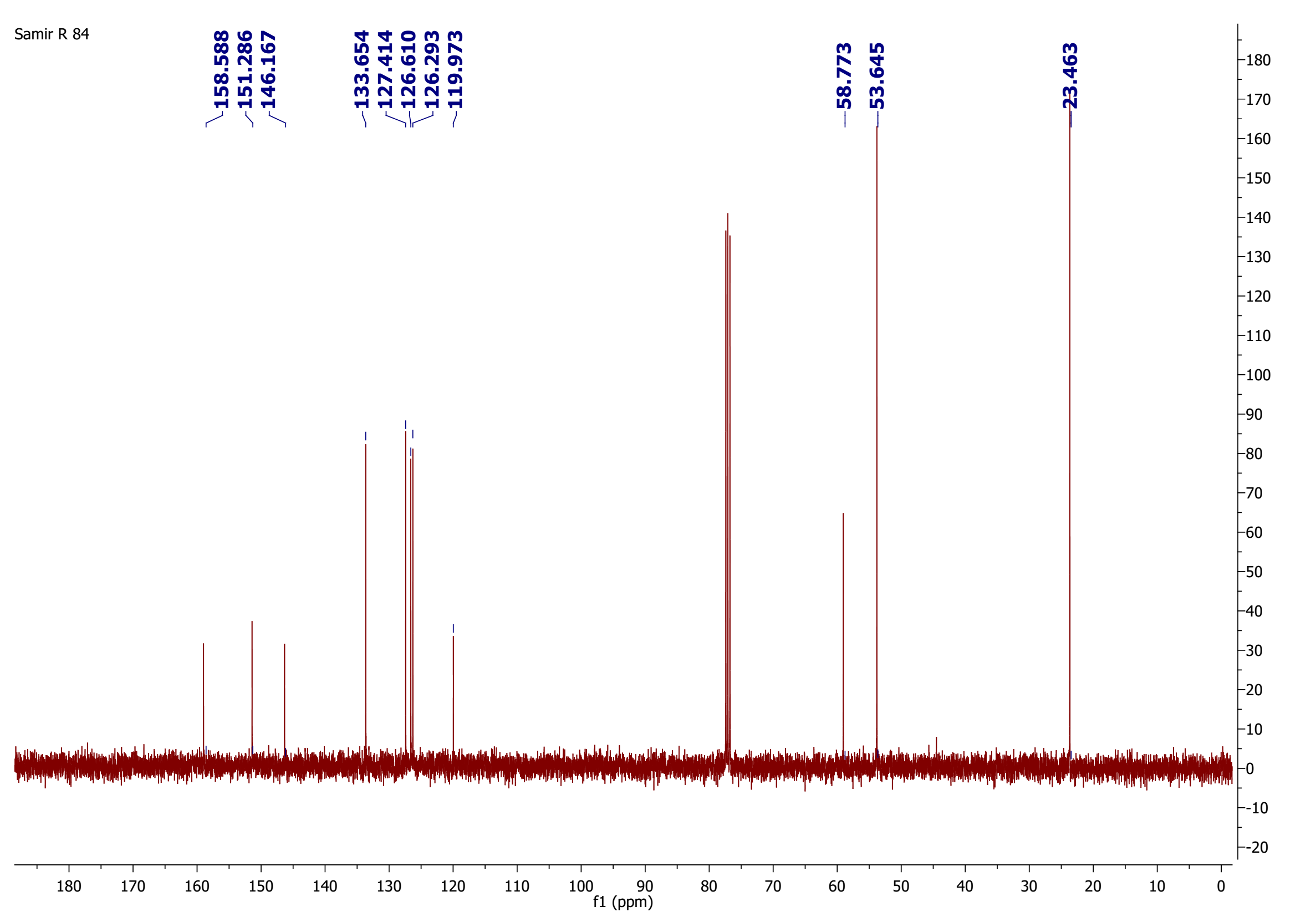


^13^C-NMR spectrum of compound **5b**

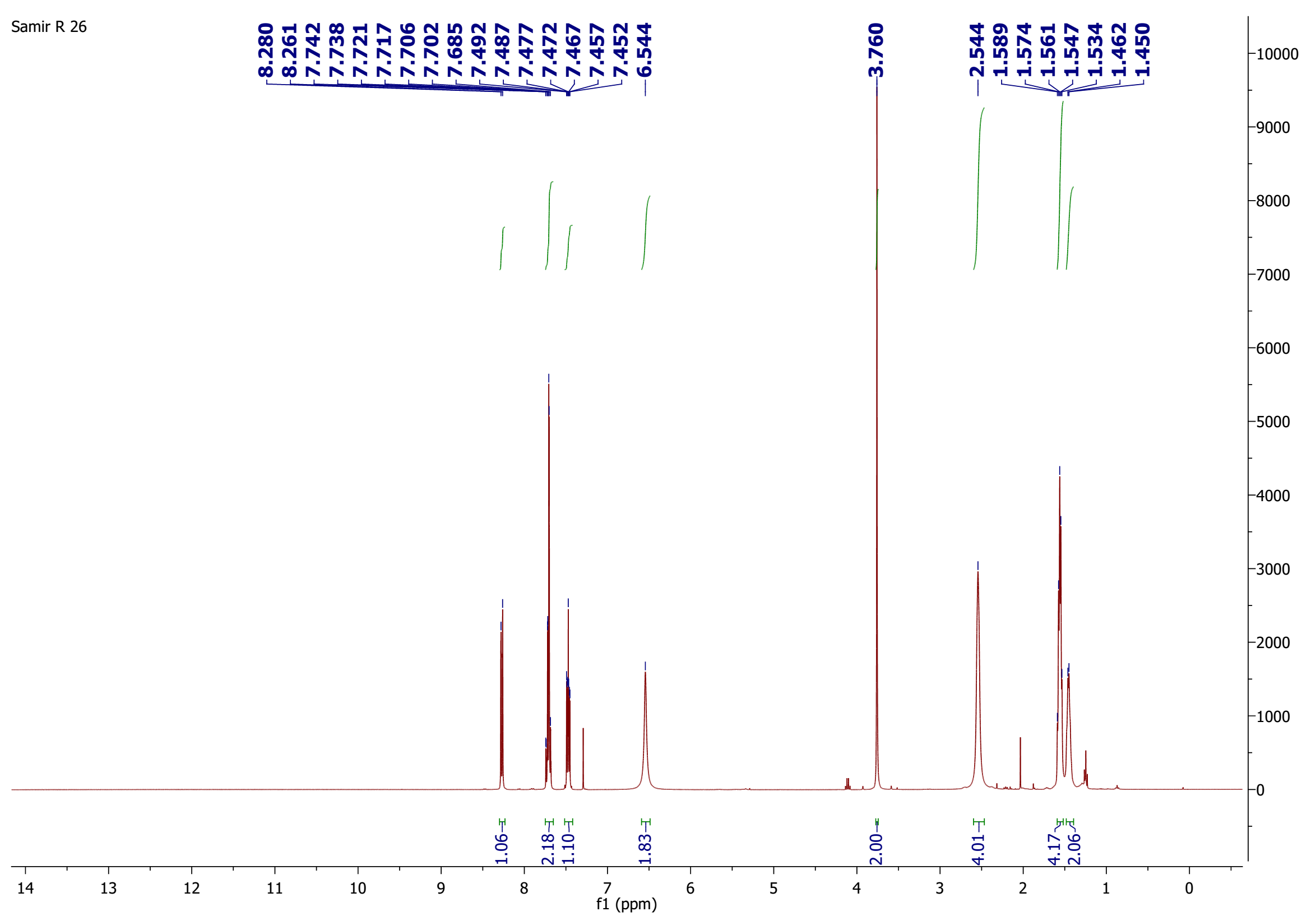


^1^H-NMR spectrum of compound **5c**


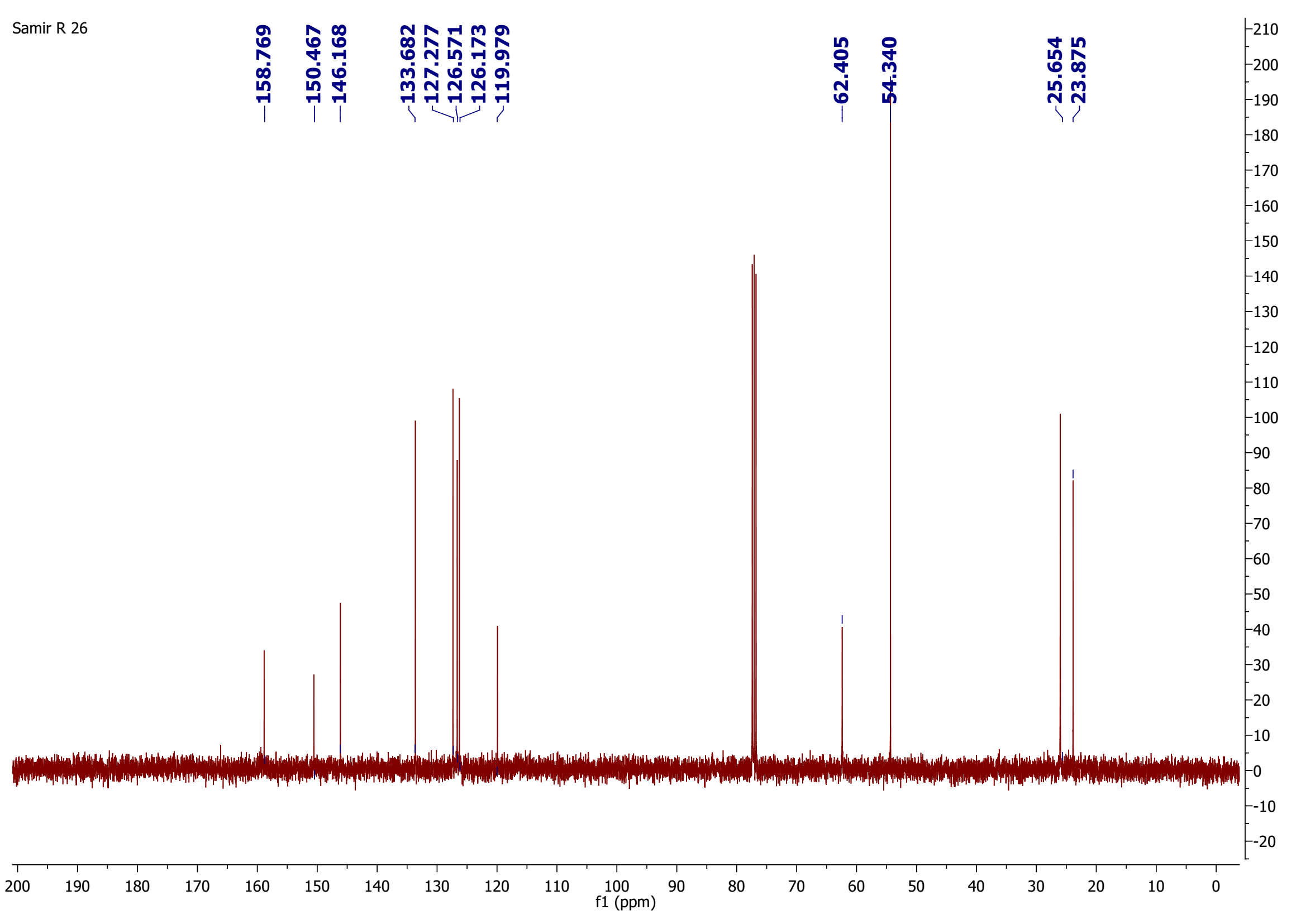


^13^C-NMR spectrum of compound **5c**

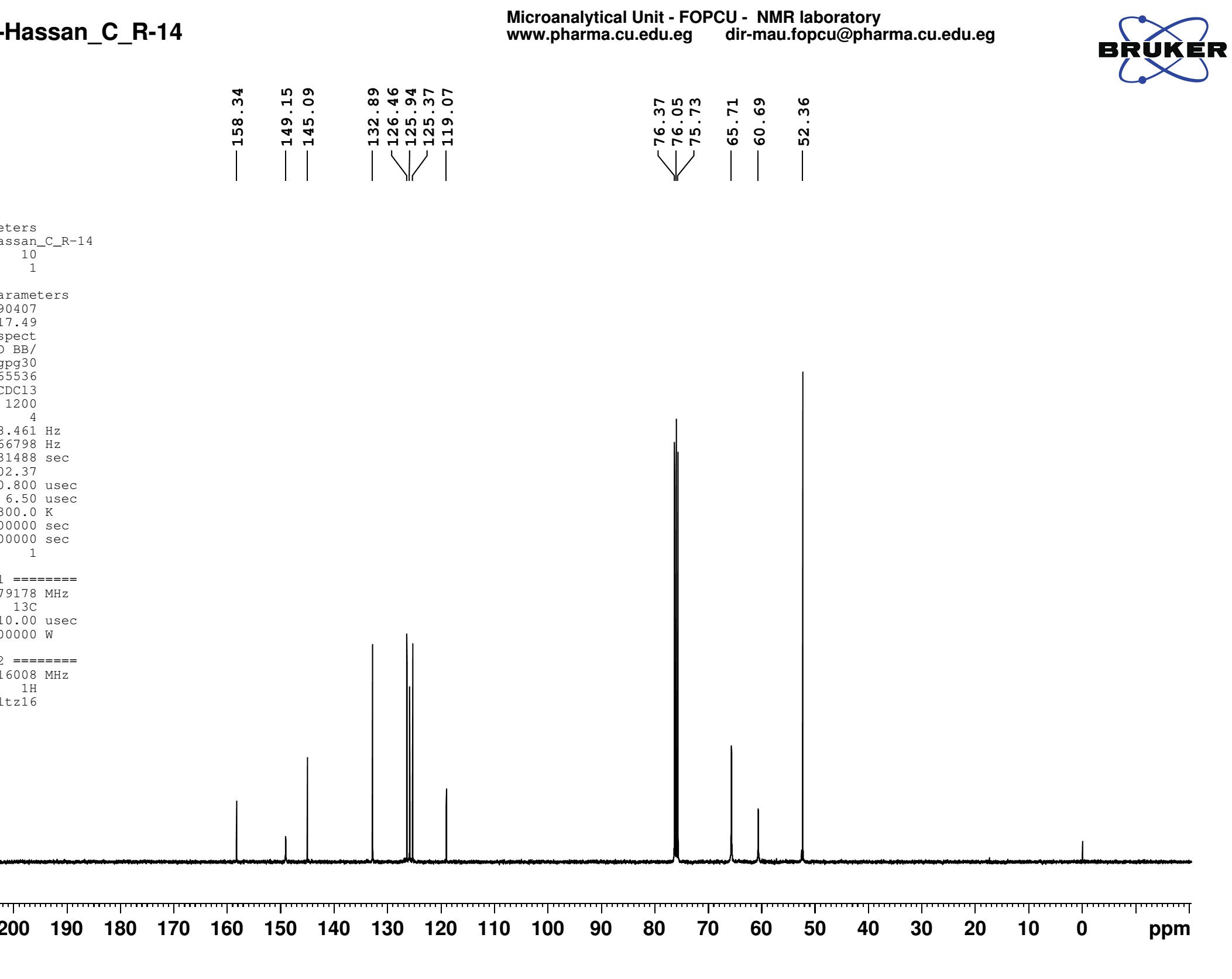


^13^C-NMR spectrum of compound **5d**

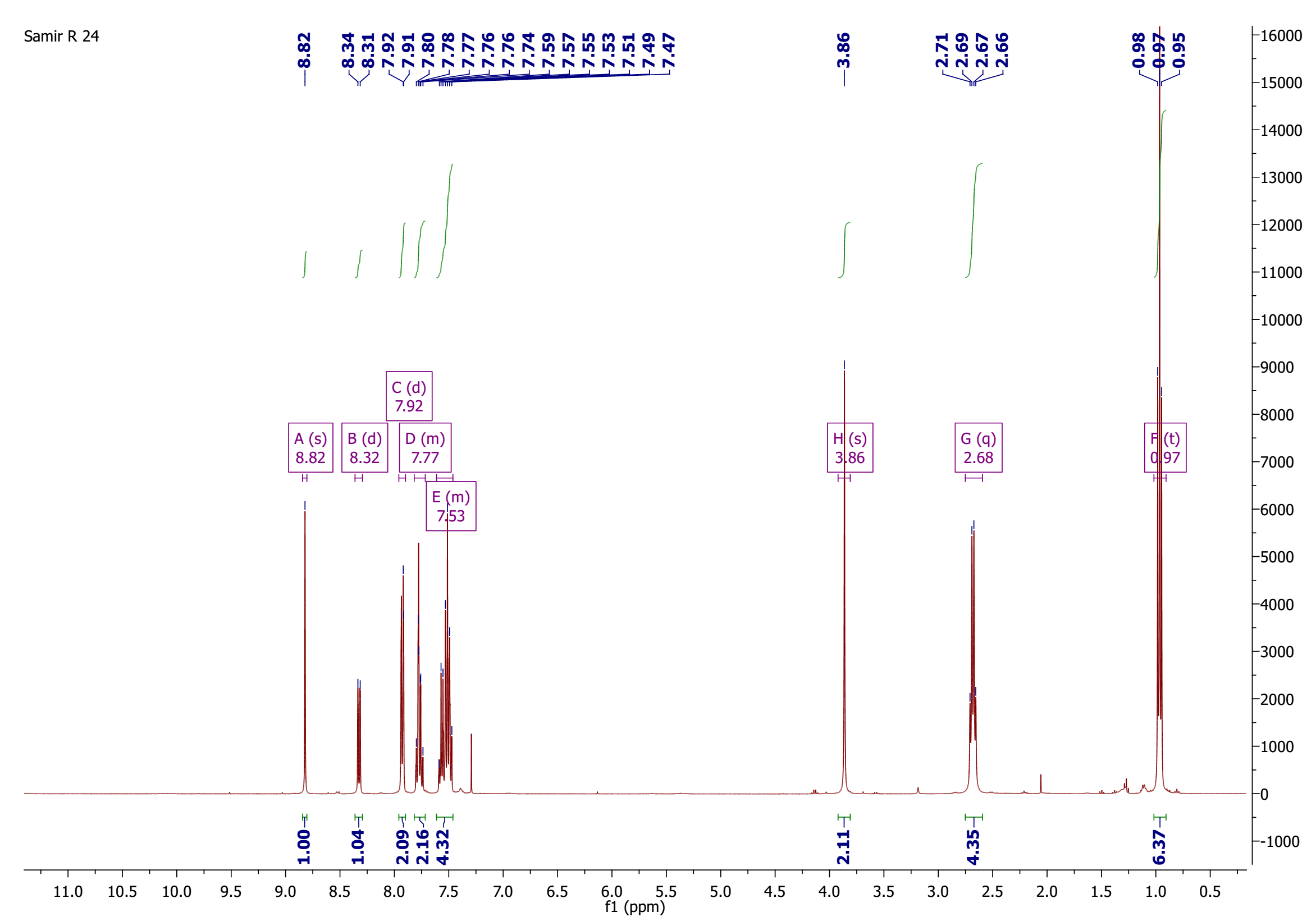


^1^H-NMR spectrum of compound **9**


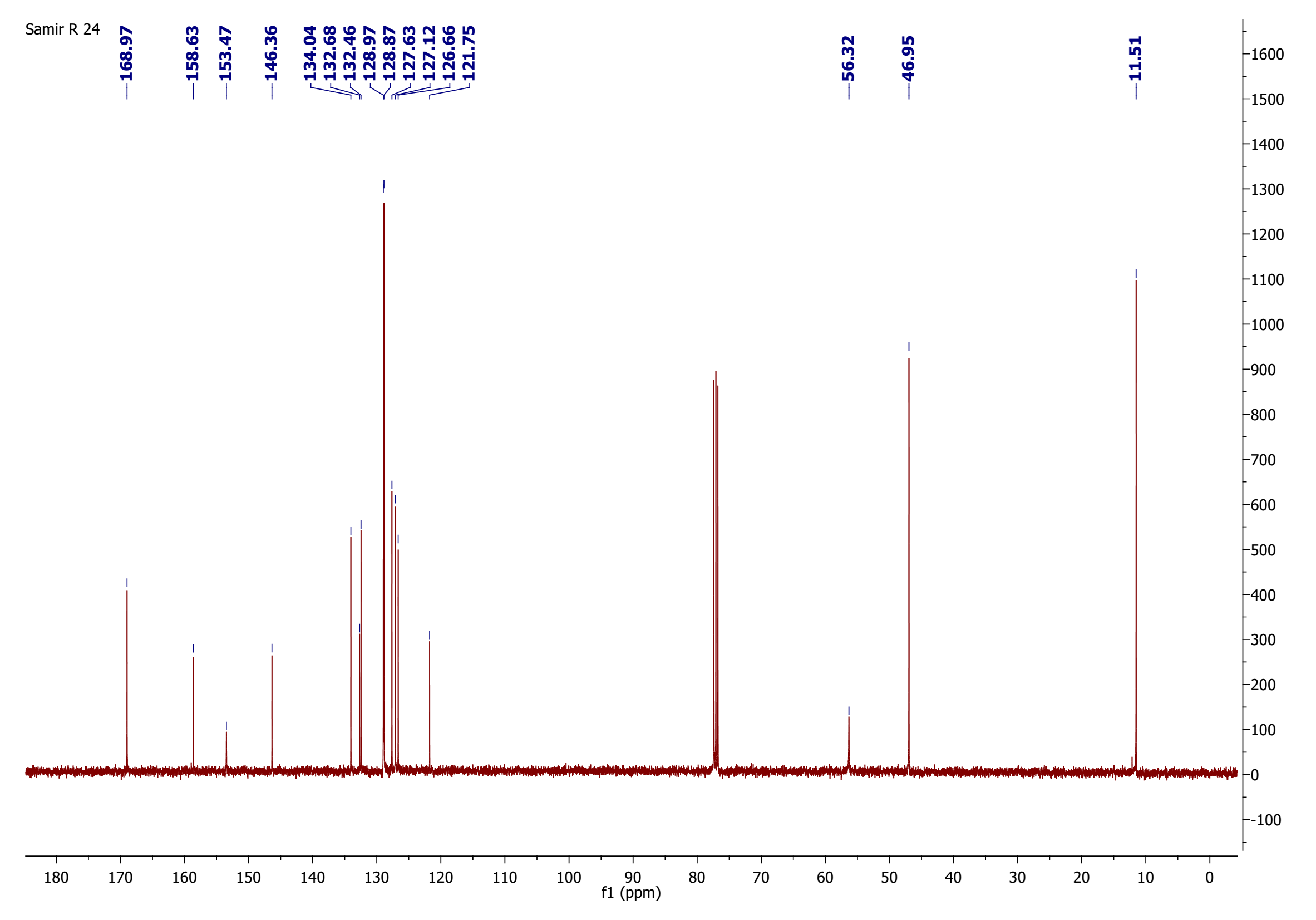


^13^C-NMR spectrum of compound **9**

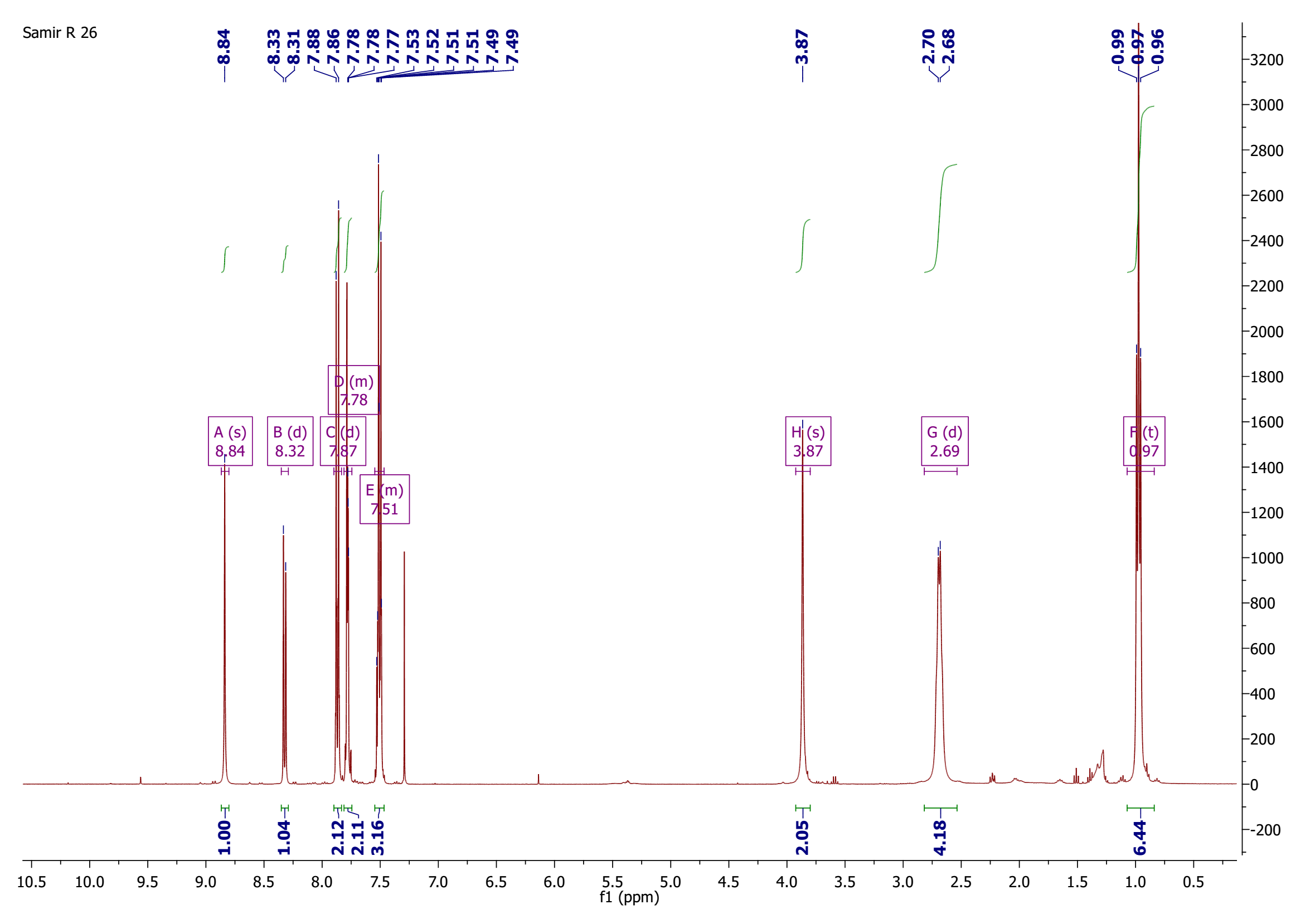


^1^H-NMR spectrum of compound **10**


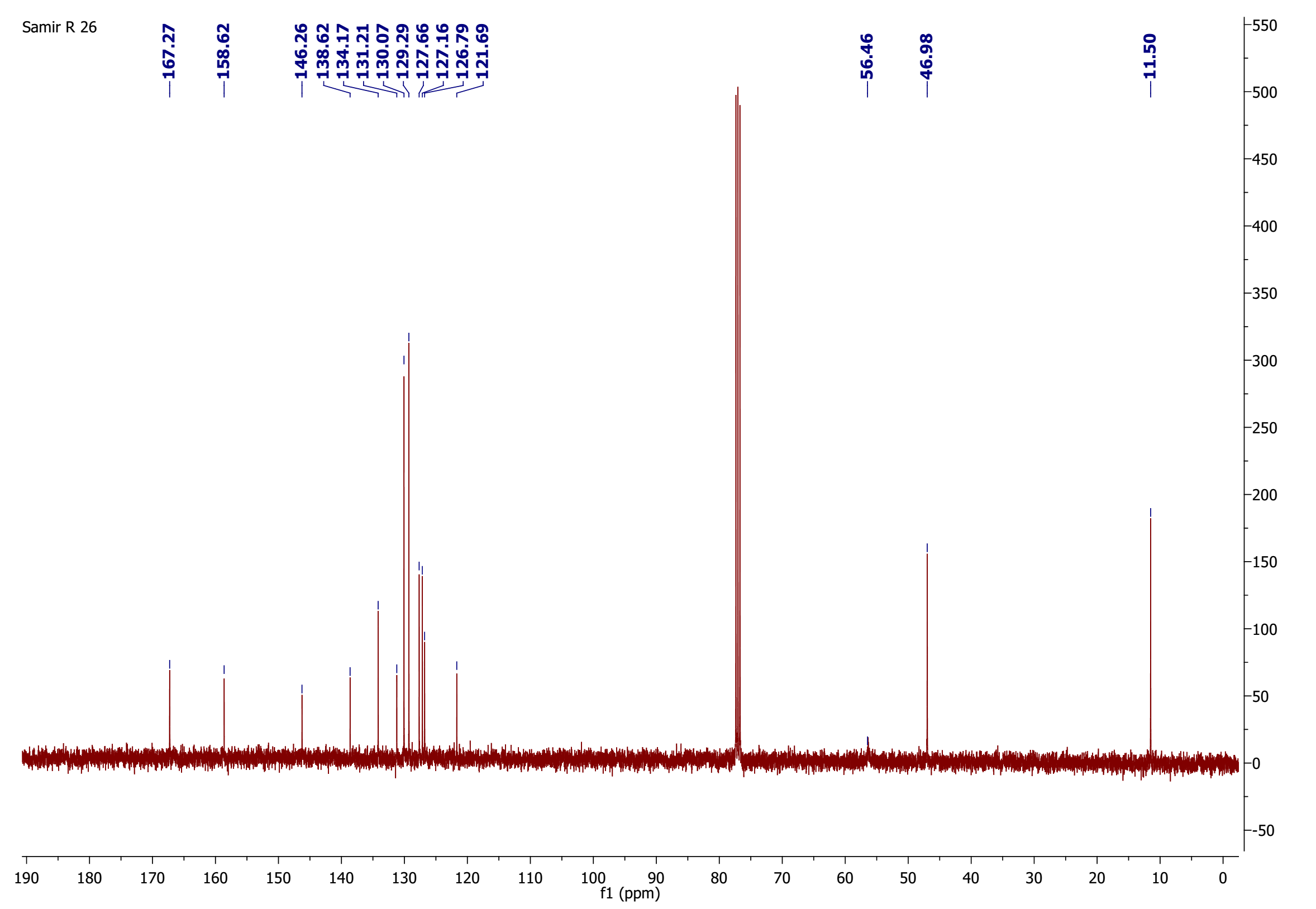


^13^C-NMR spectrum of compound **10**

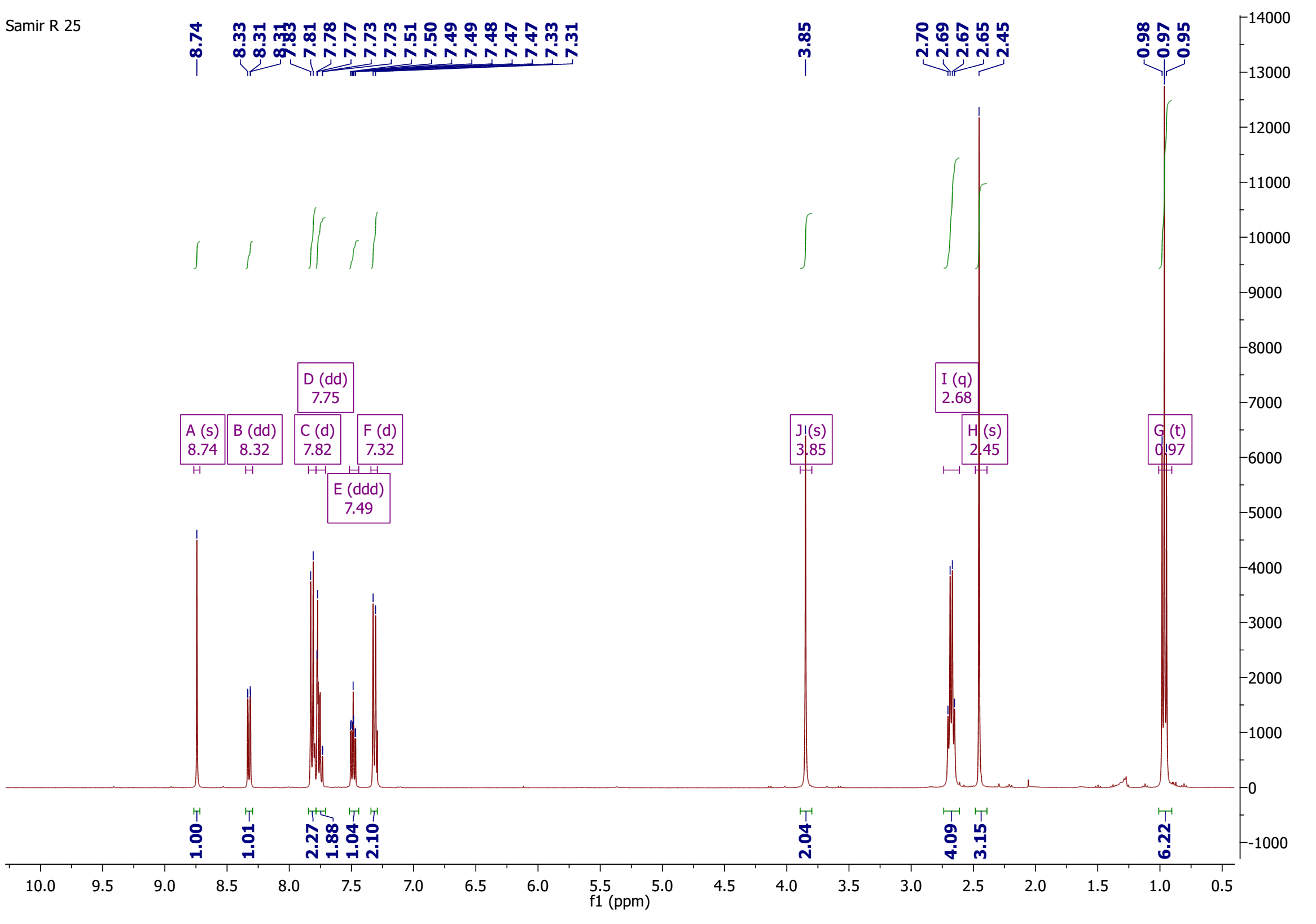


^1^H-NMR spectrum of compound **11**


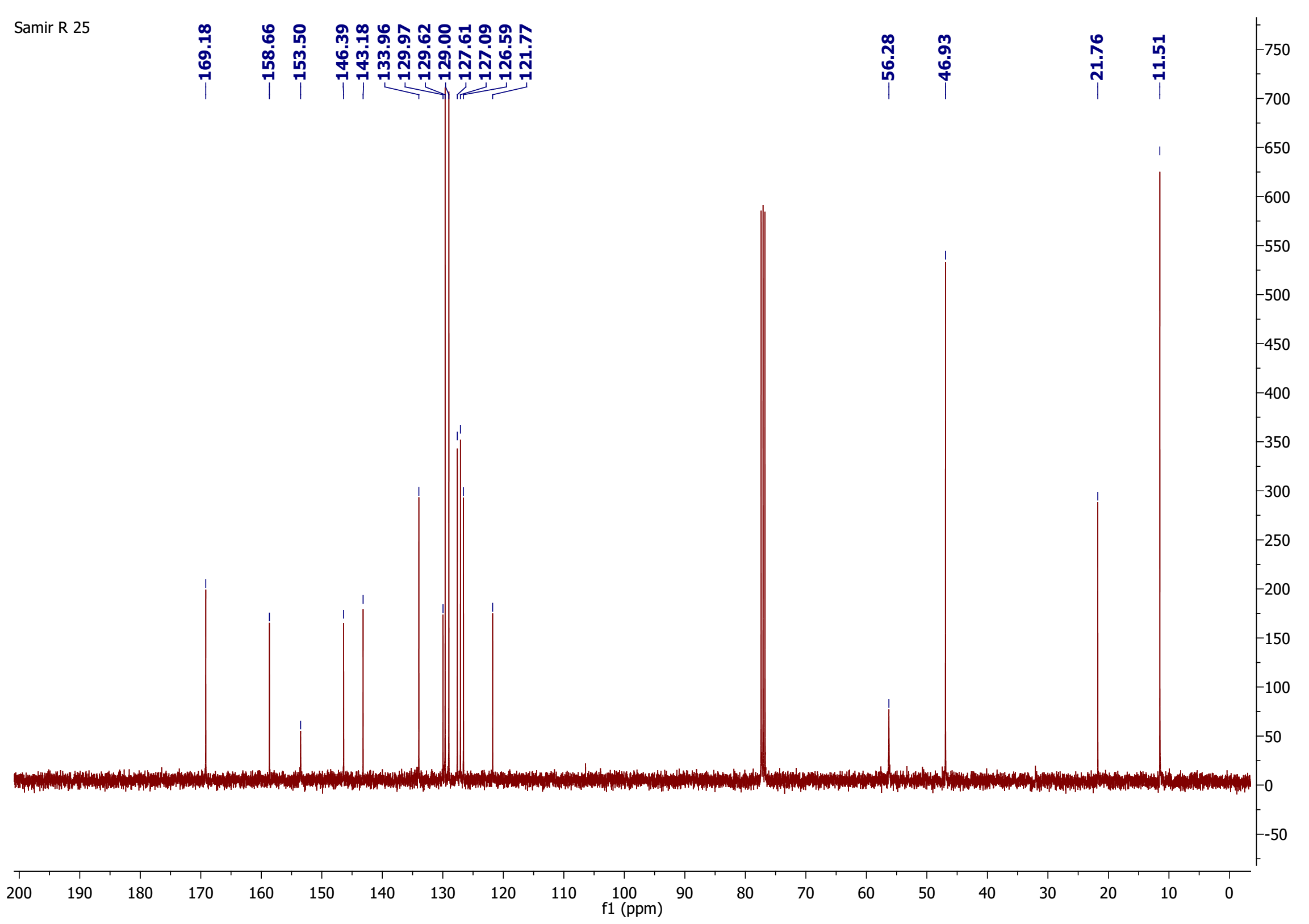


^13^C-NMR spectrum of compound **11**

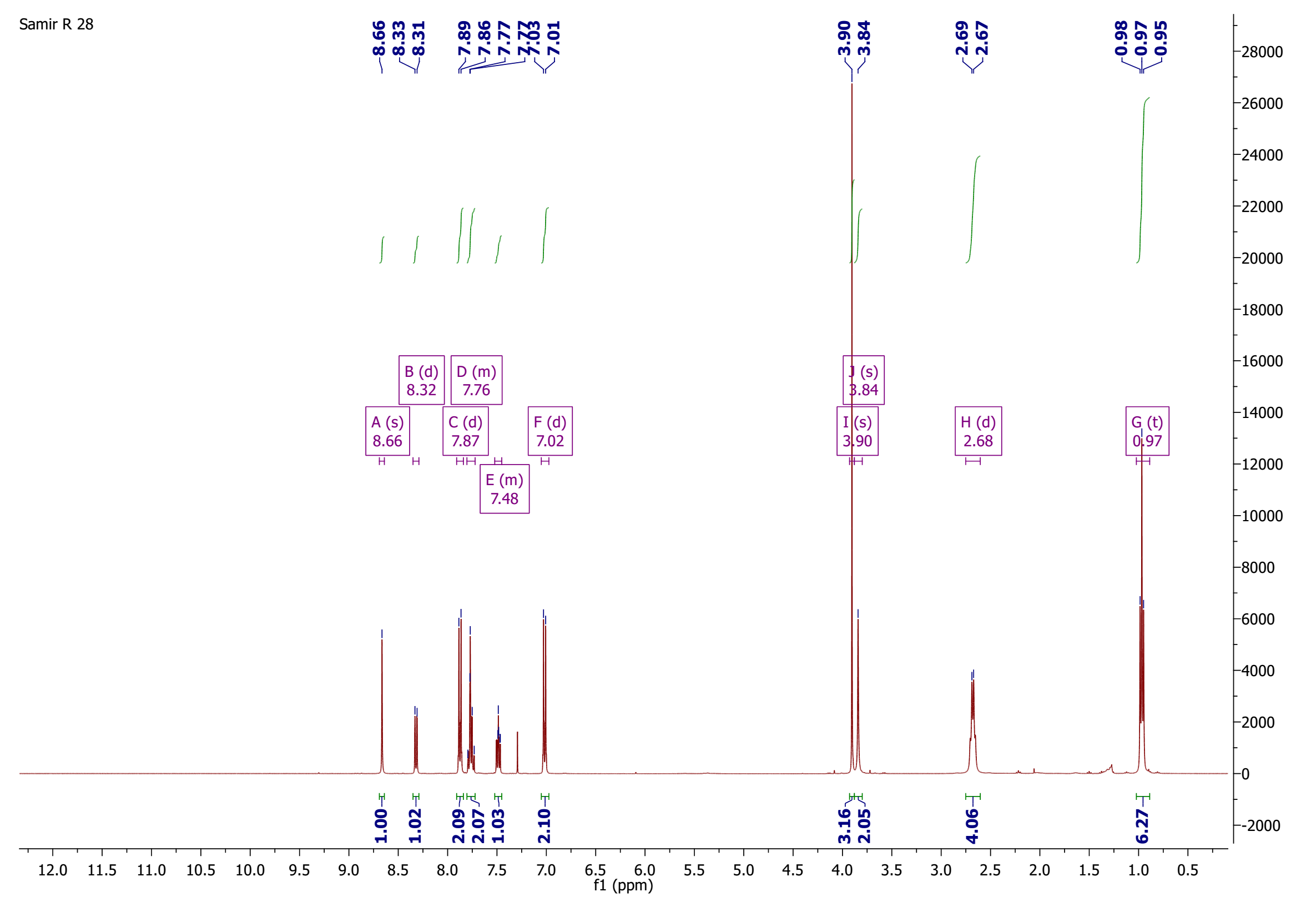


^1^H-NMR spectrum of compound **12**


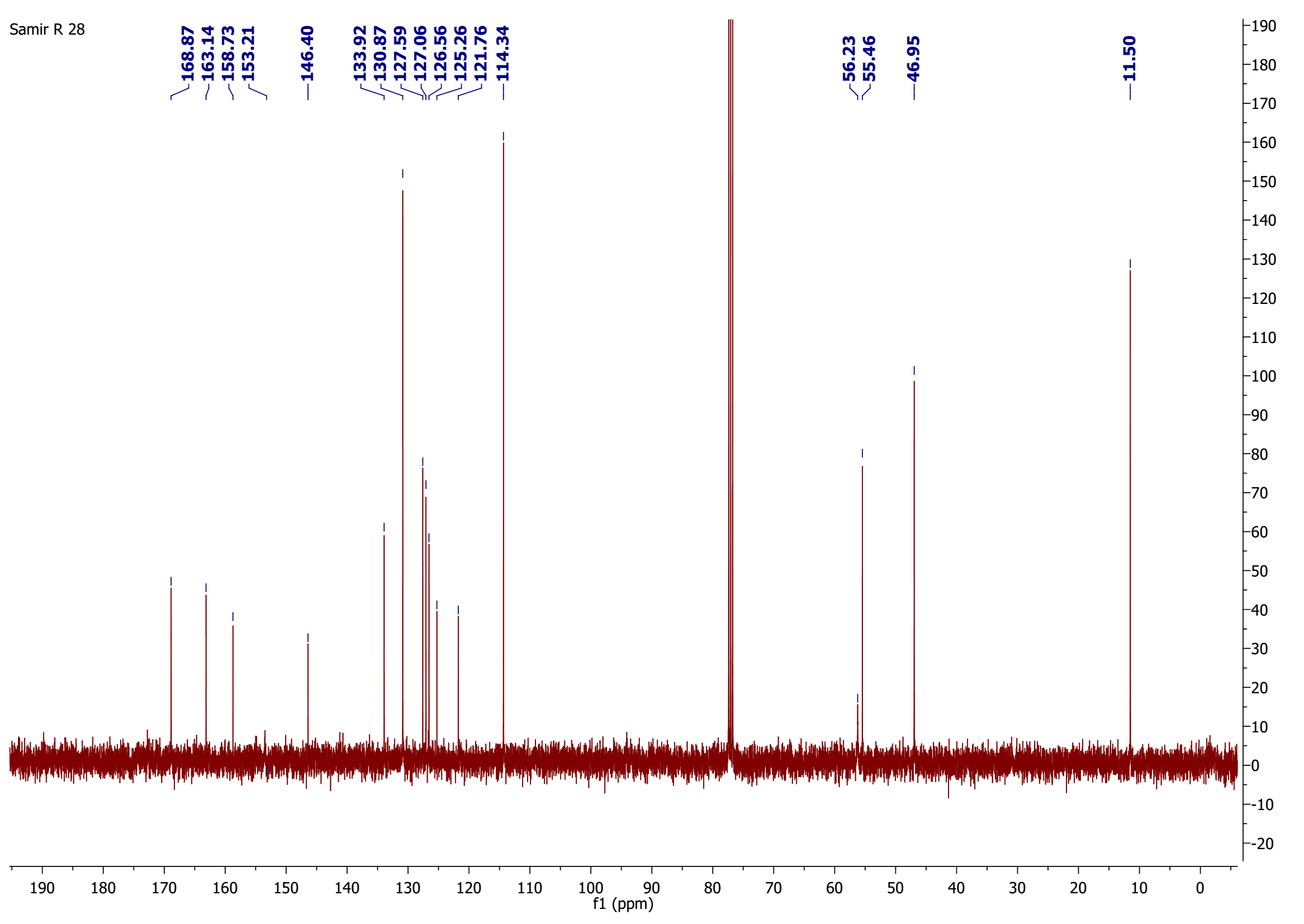


^13^C-NMR spectrum of compound **12**

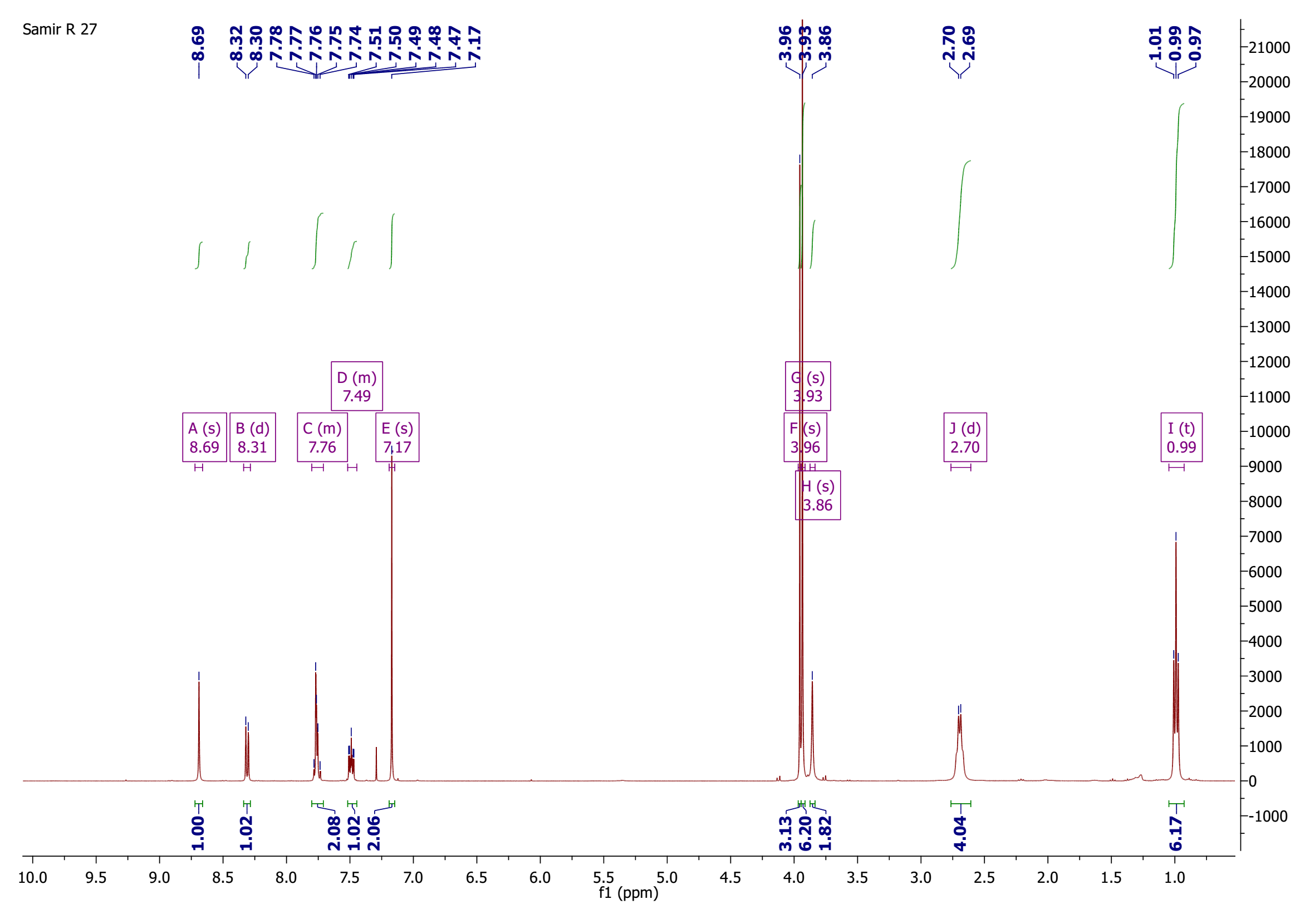


^1^H-NMR spectrum of compound **13**


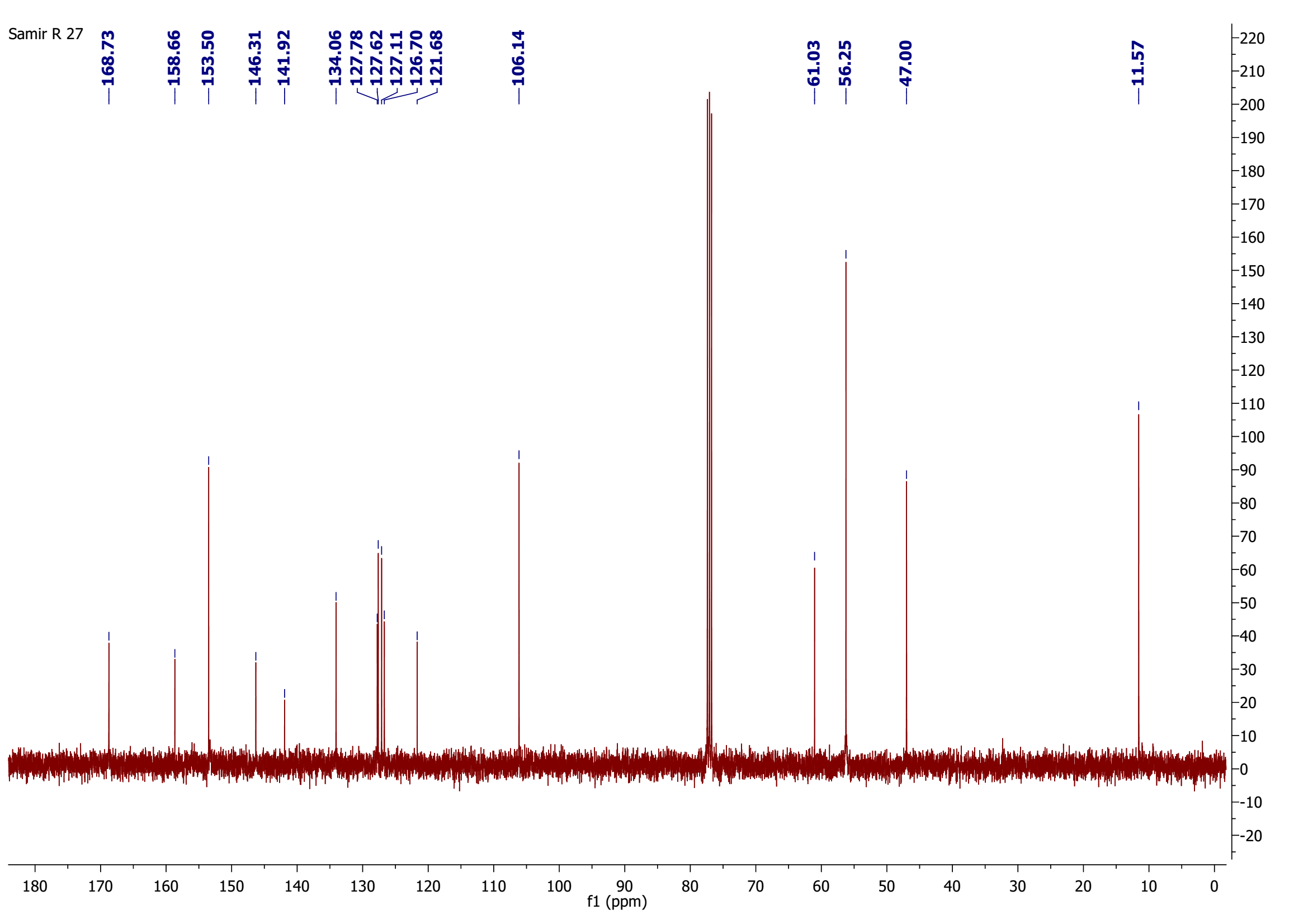


^13^C-NMR spectrum of compound **13**

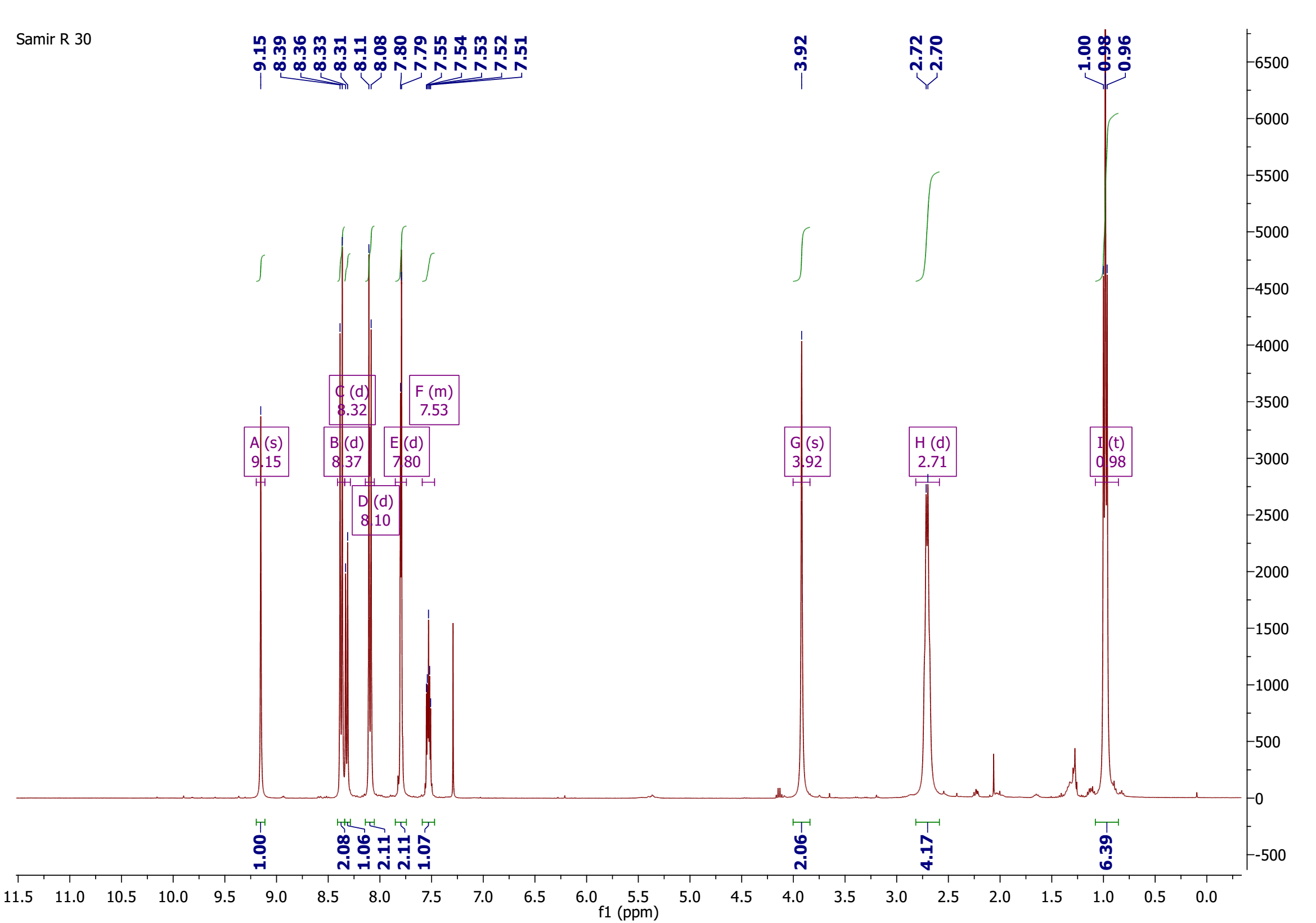


^1^H-NMR spectrum of compound **14**


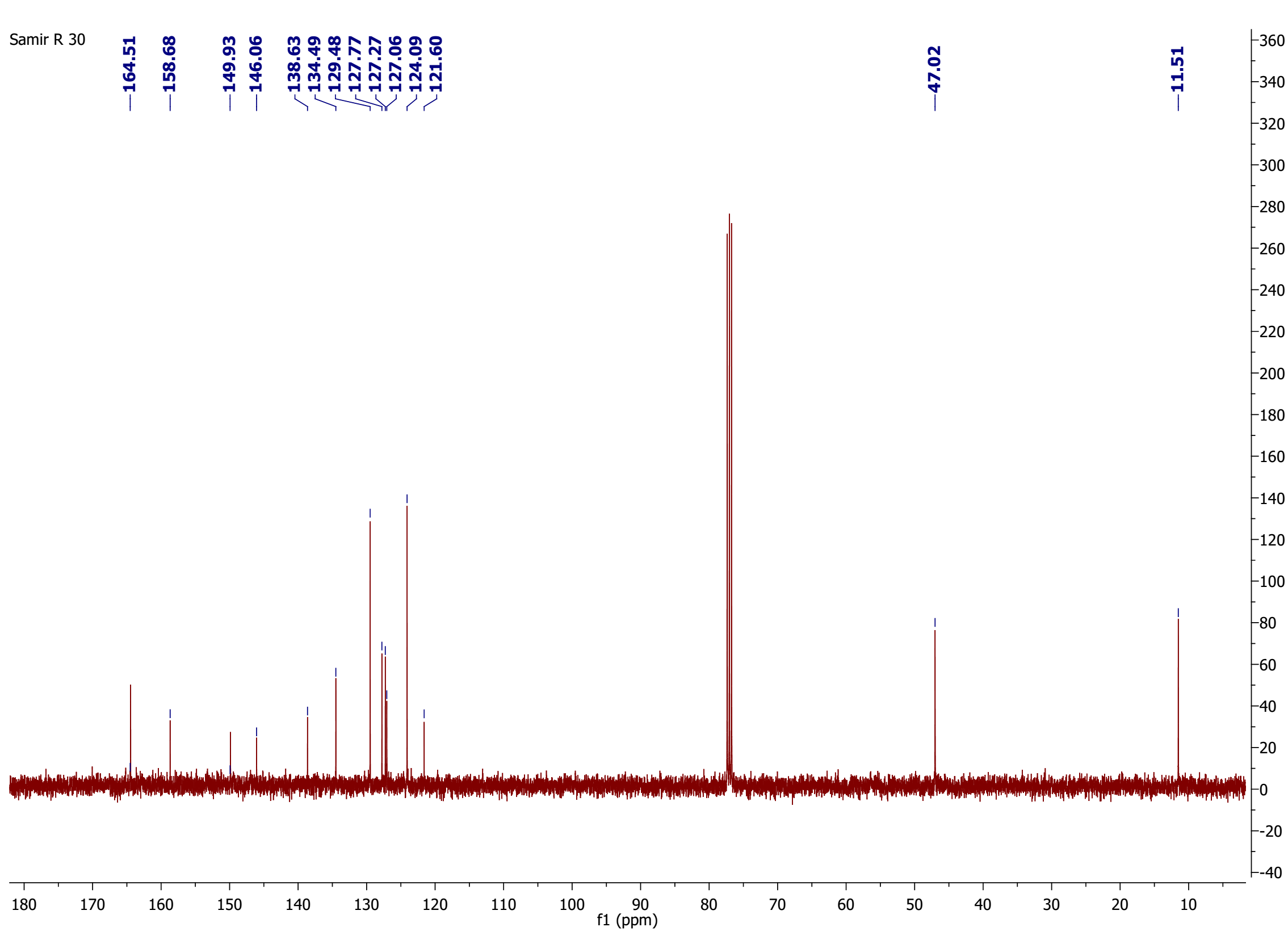


^13^C-NMR spectrum of compound **14**

^1^H-NMR spectrum of compound **15**


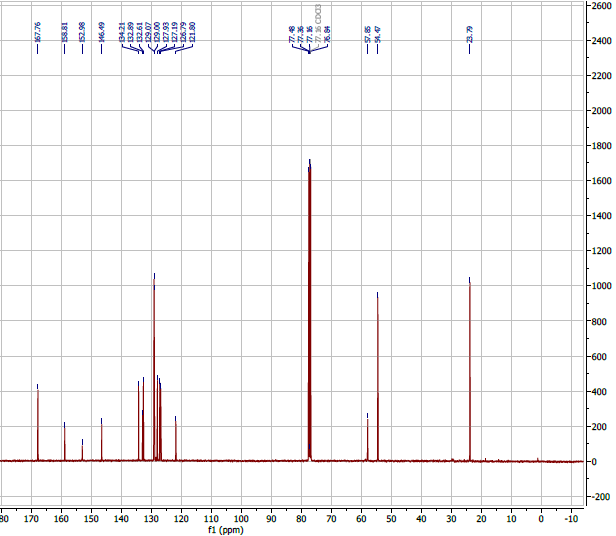


^13^C-NMR spectrum of compound **15**

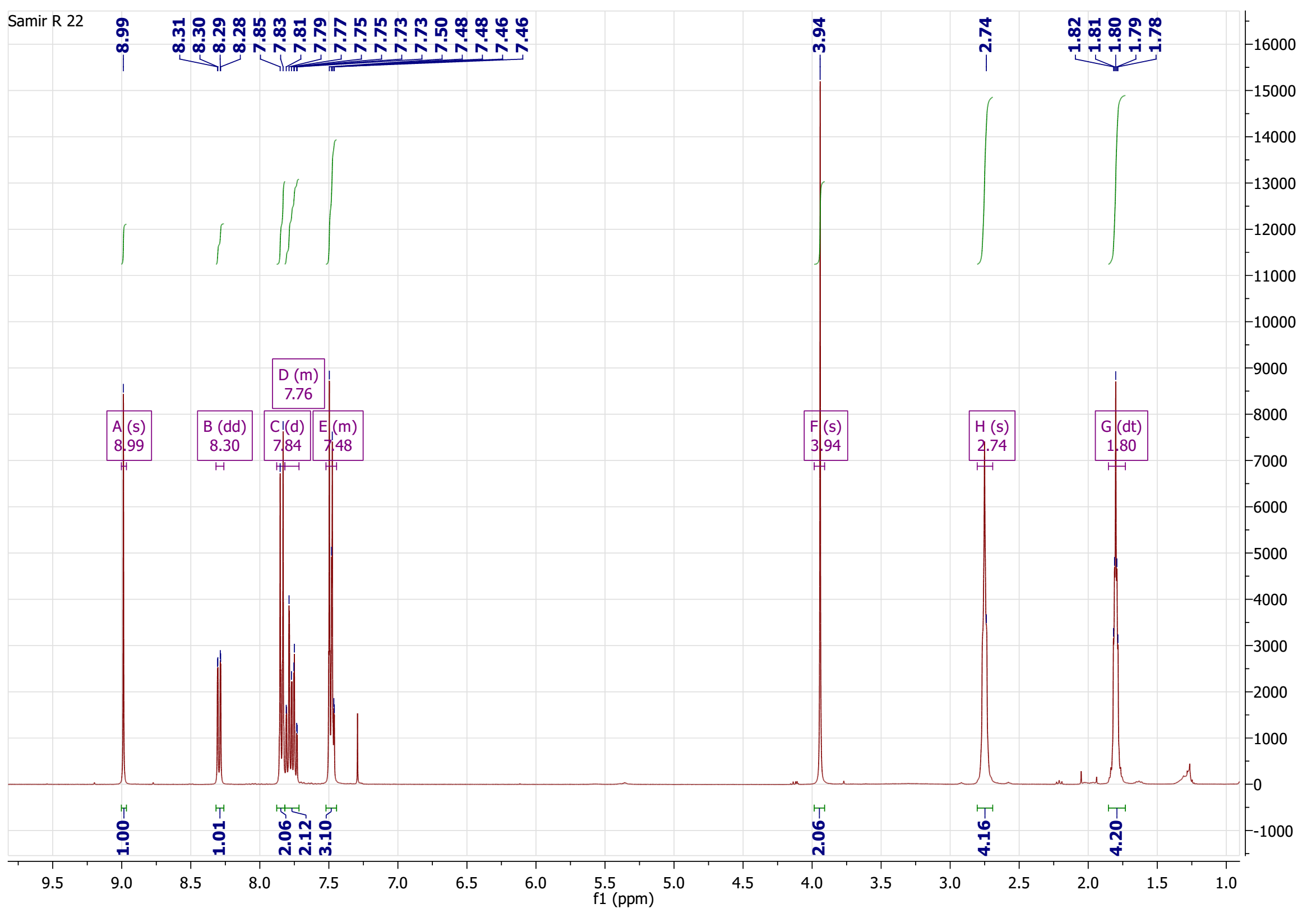


^1^H-NMR spectrum of compound **16**


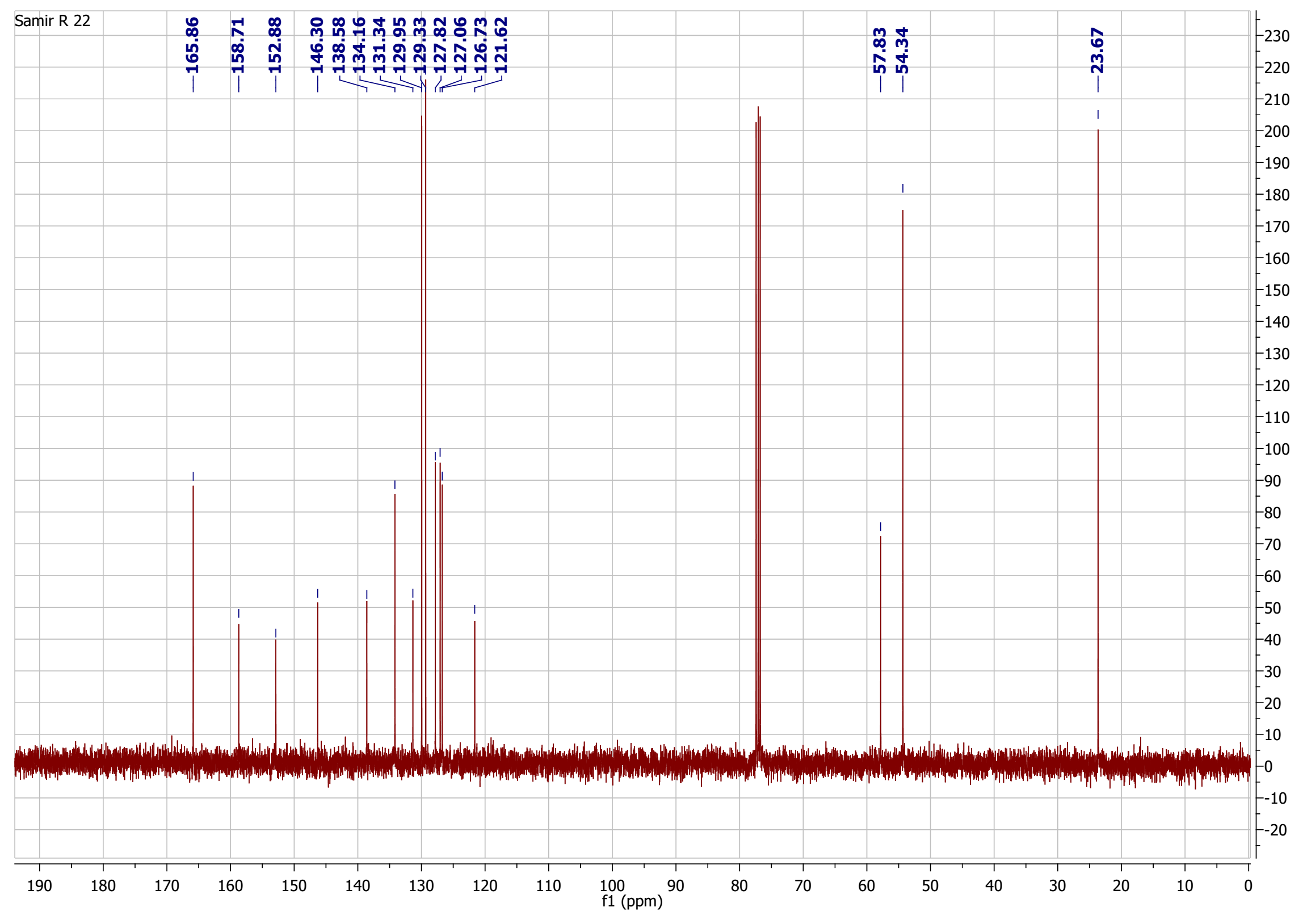


^13^C-NMR spectrum of compound **16**

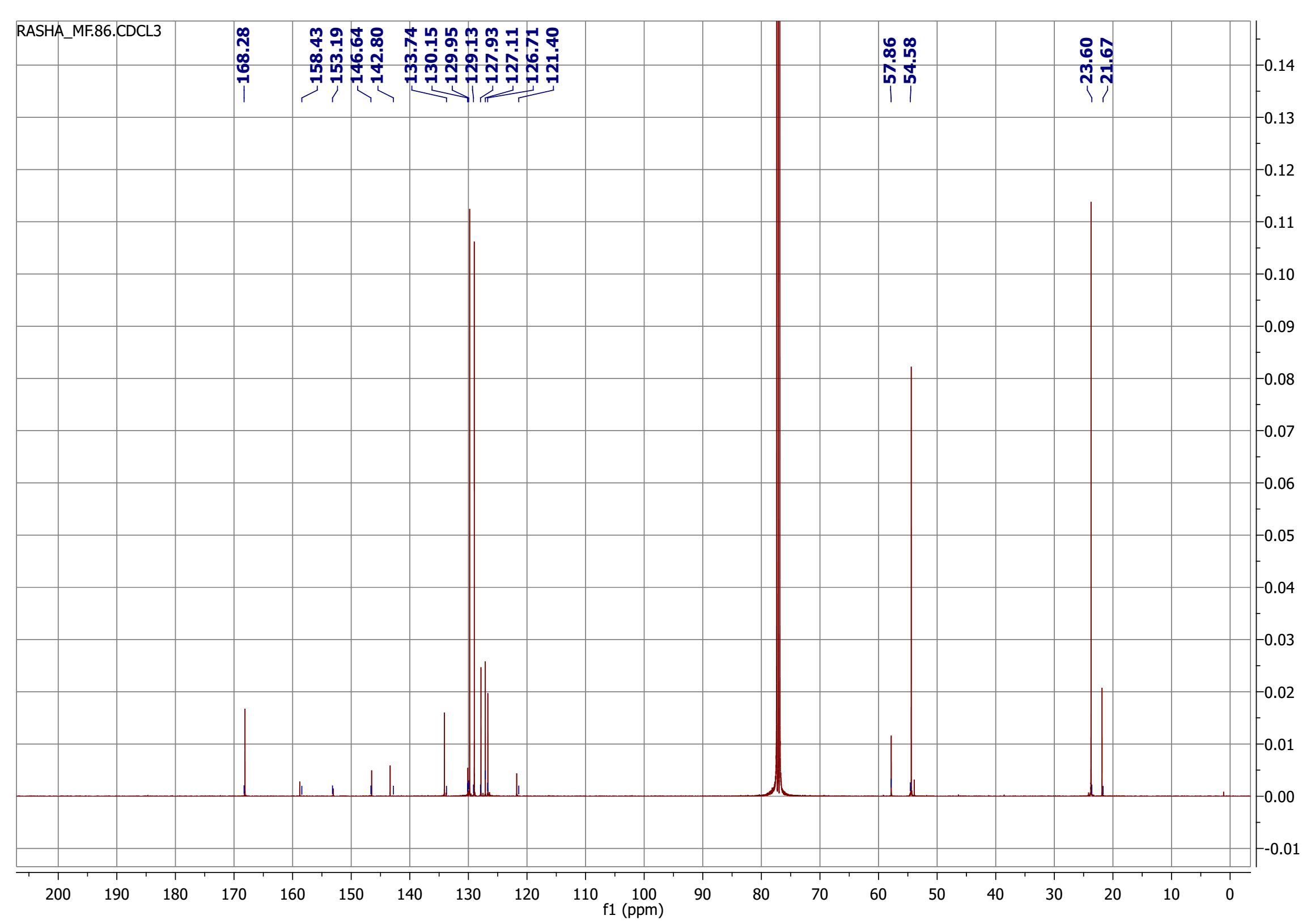


^13^C-NMR spectrum of compound **17**

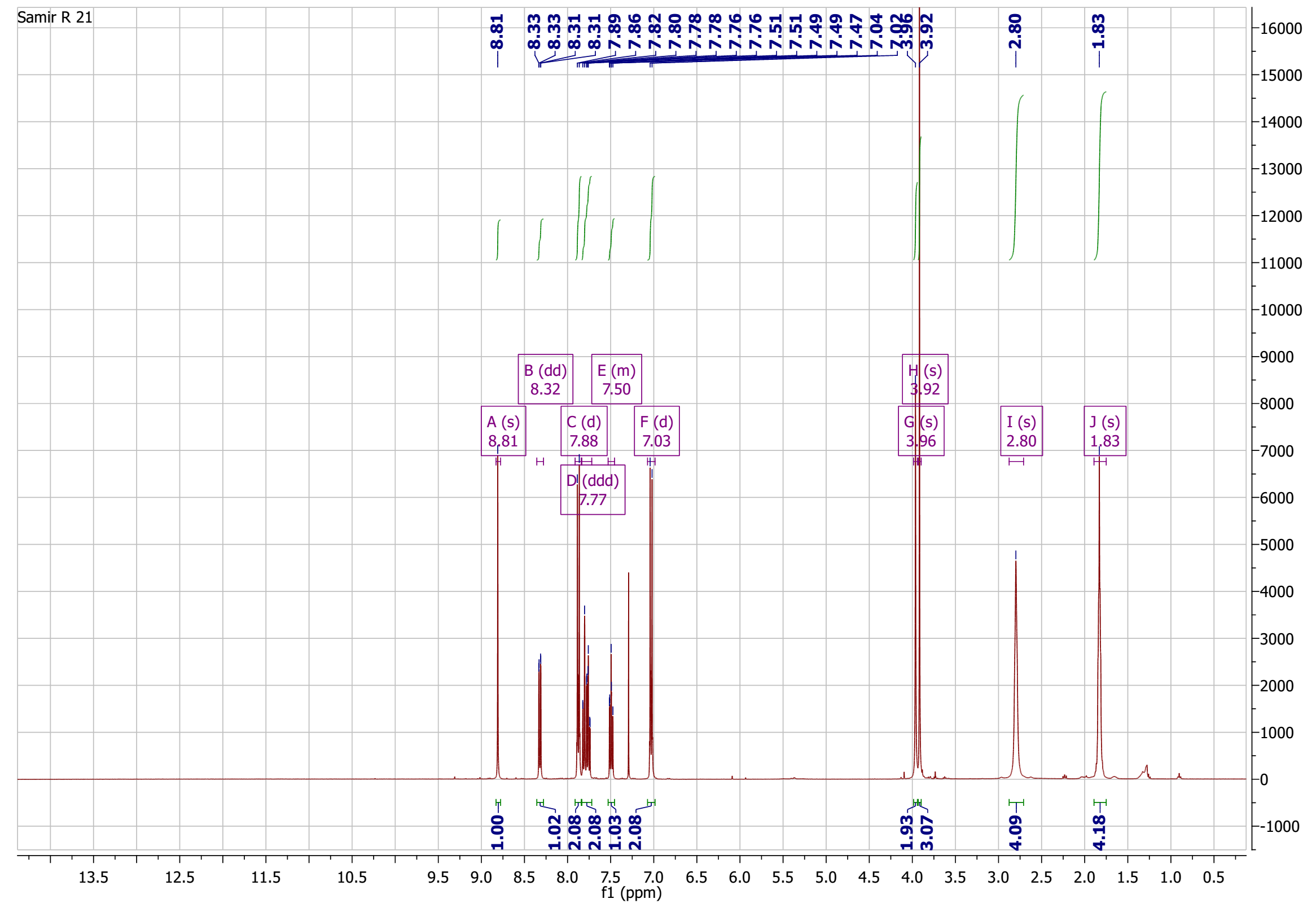


^1^H-NMR spectrum of compound **18**


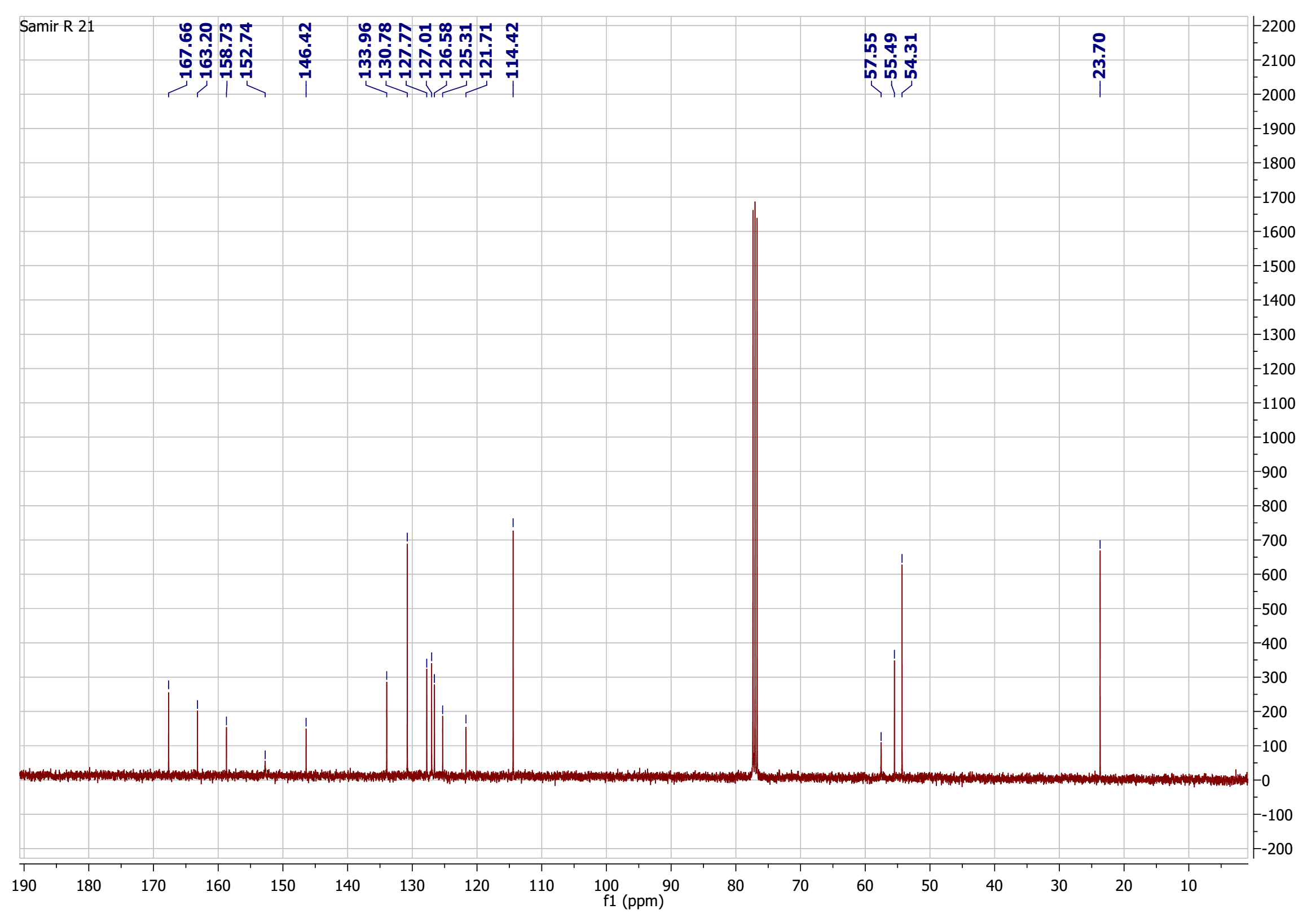


^13^C-NMR spectrum of compound **18**

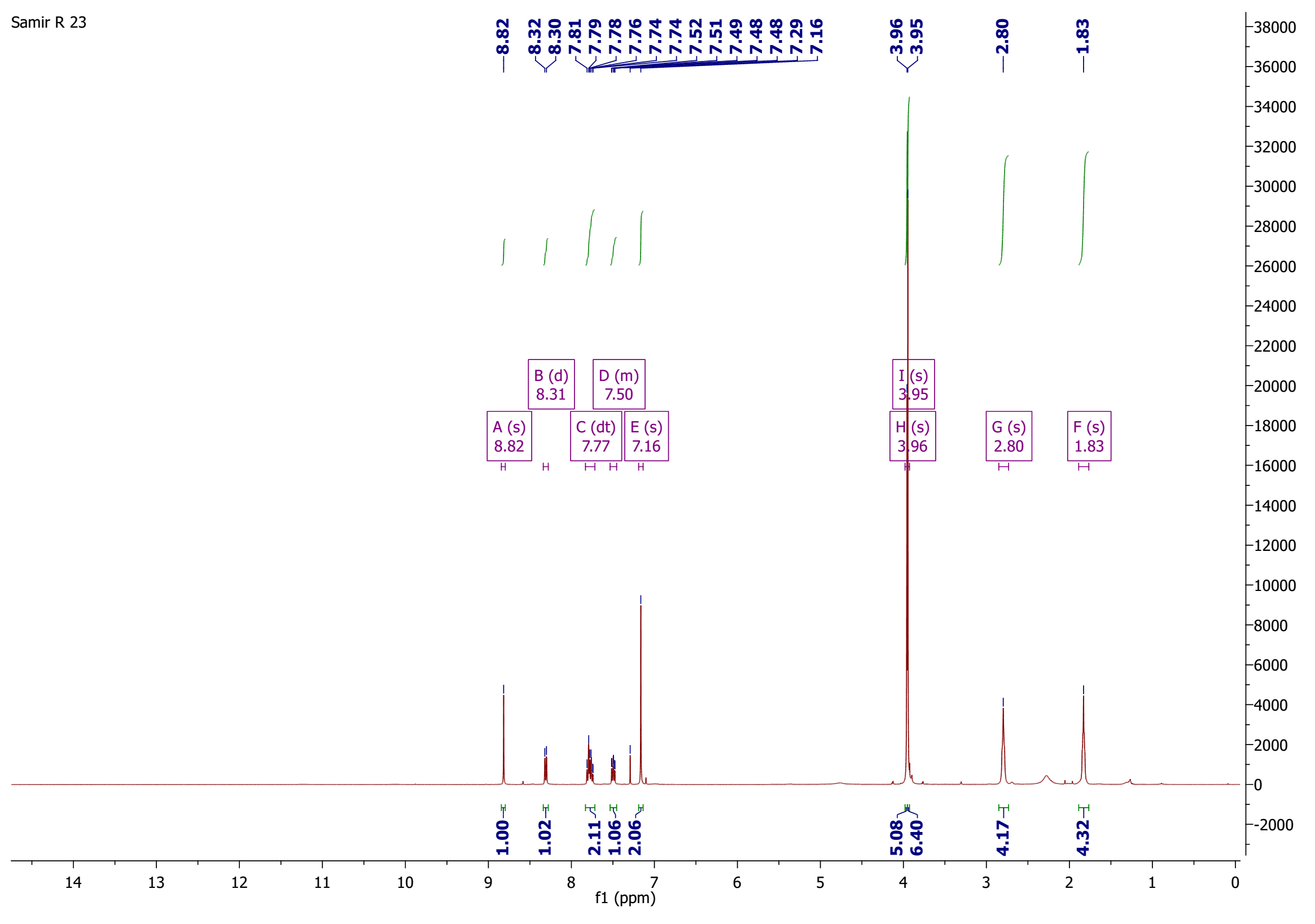


^1^H-NMR spectrum of compound **19**


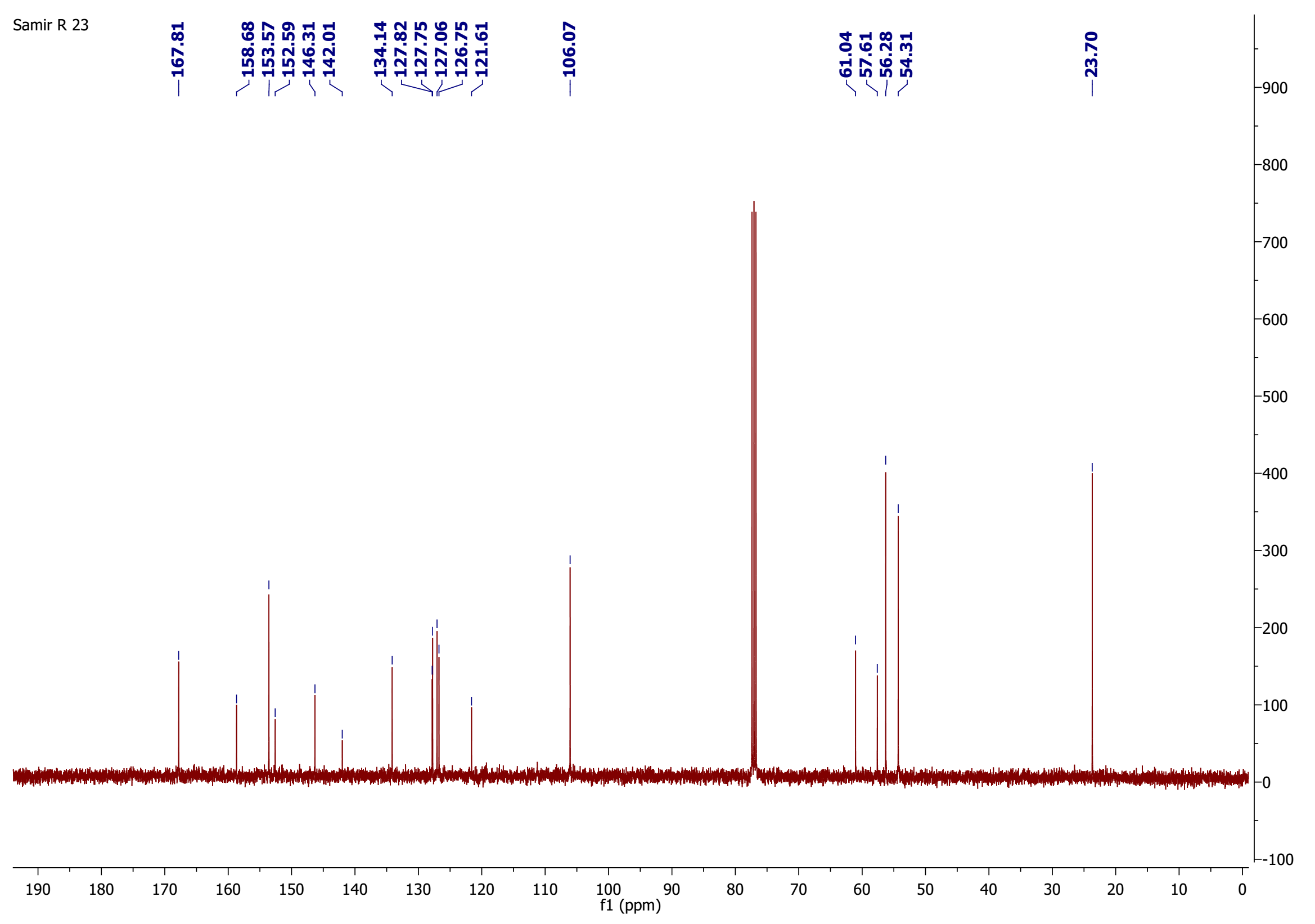


^13^C-NMR spectrum of compound **19**

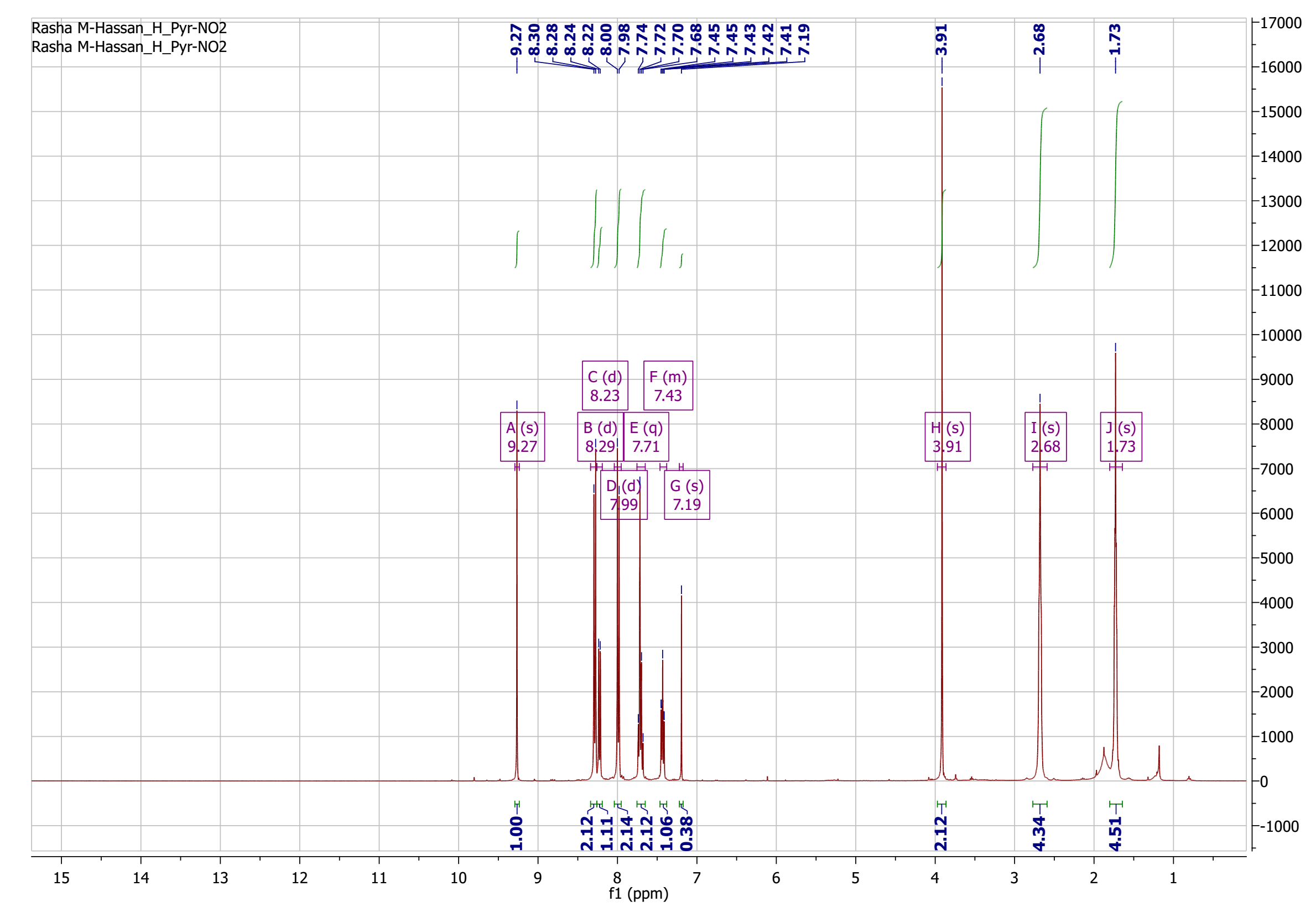


^1^H-NMR spectrum of compound **20**


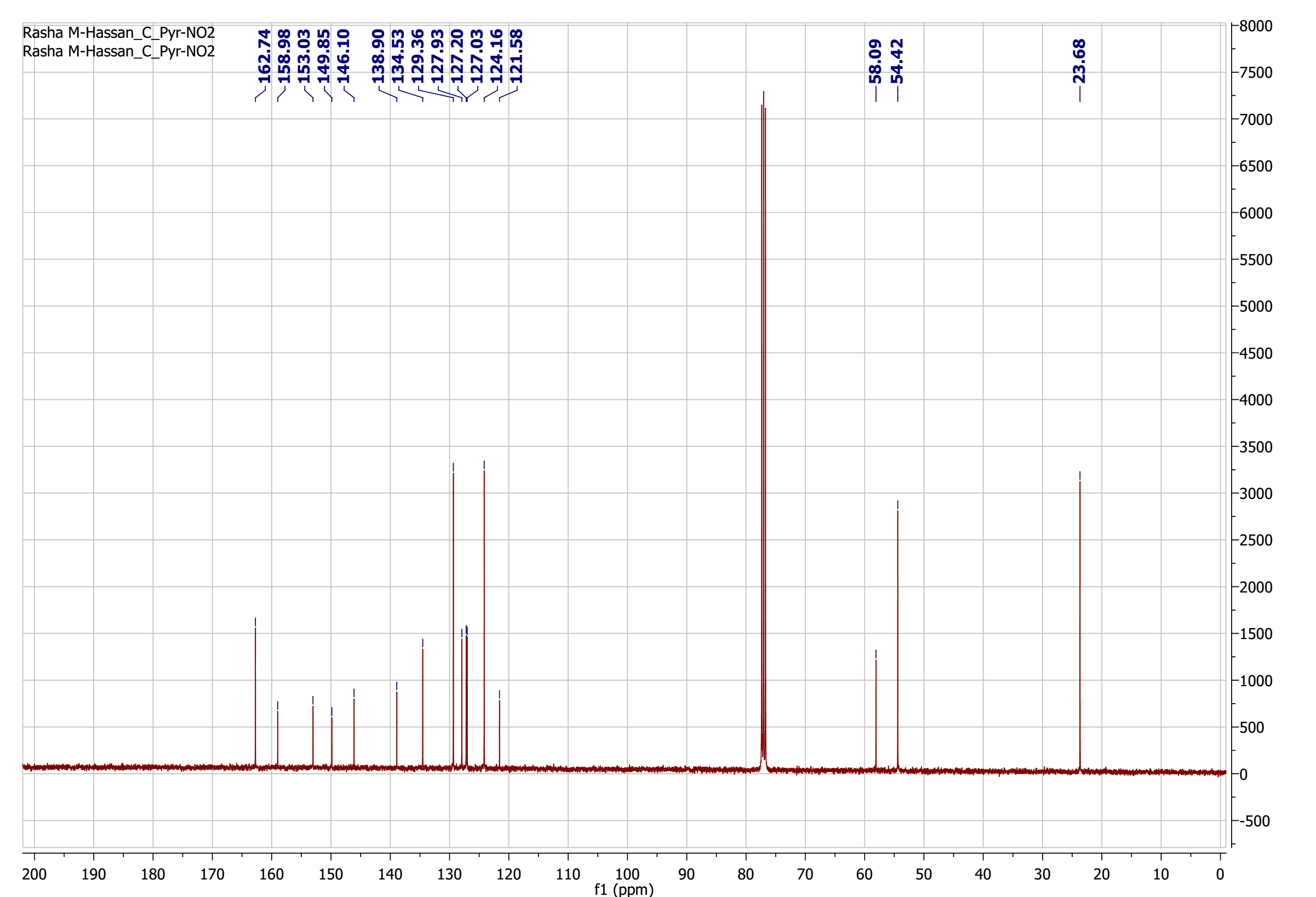


^13^C-NMR spectrum of compound **20**

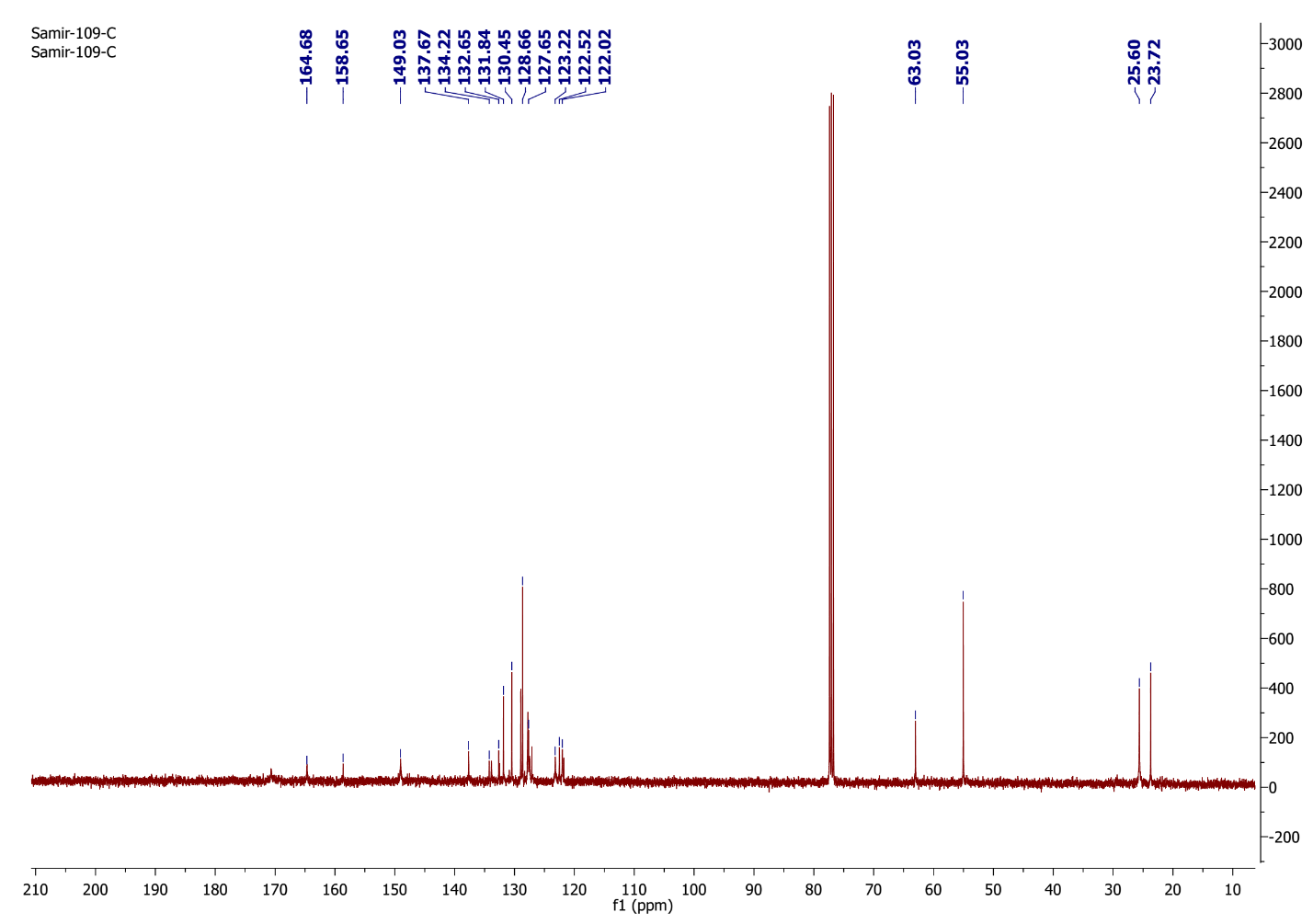


^13^C-NMR spectrum of compound **21**

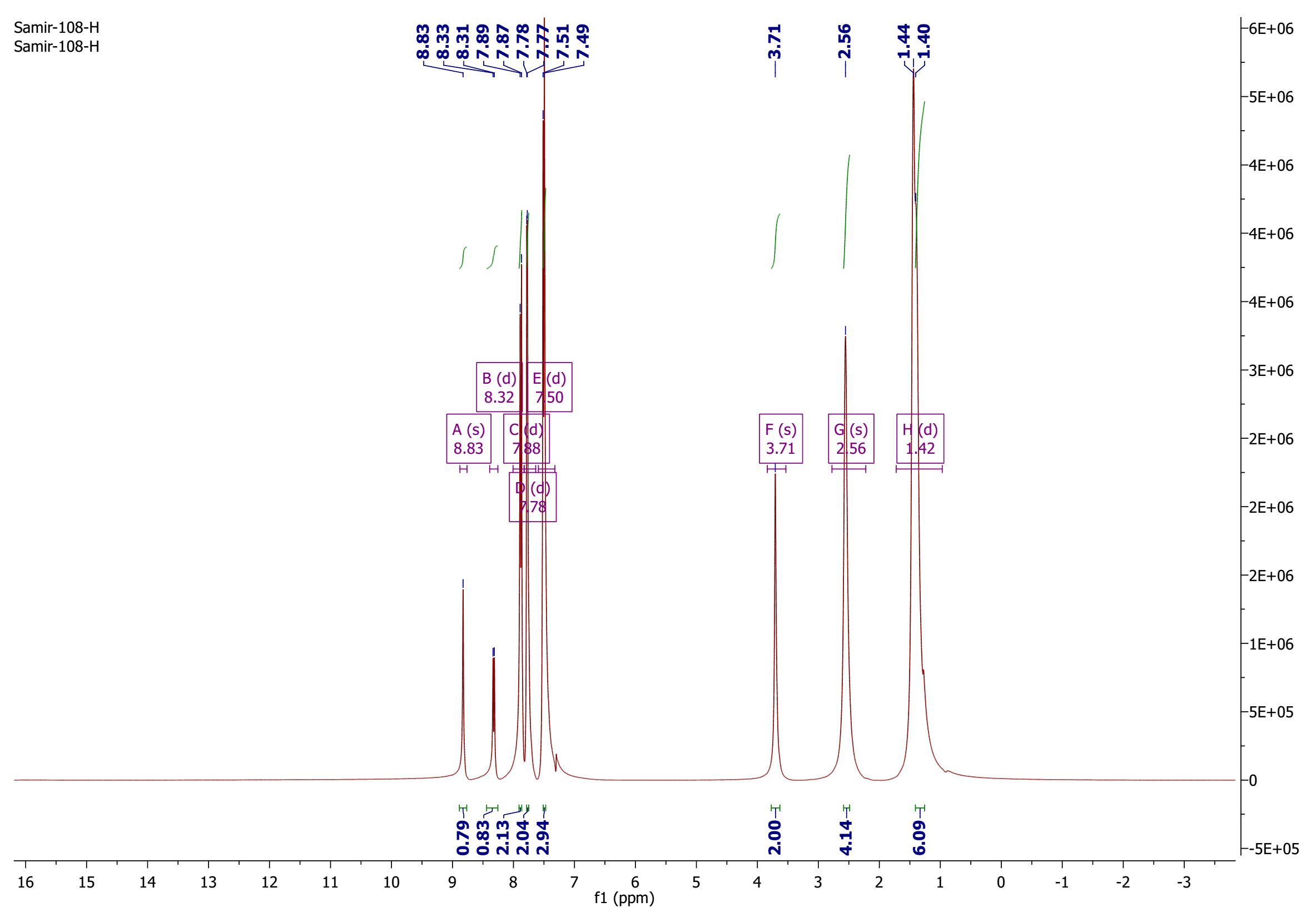


^1^H-NMR spectrum of compound **22**


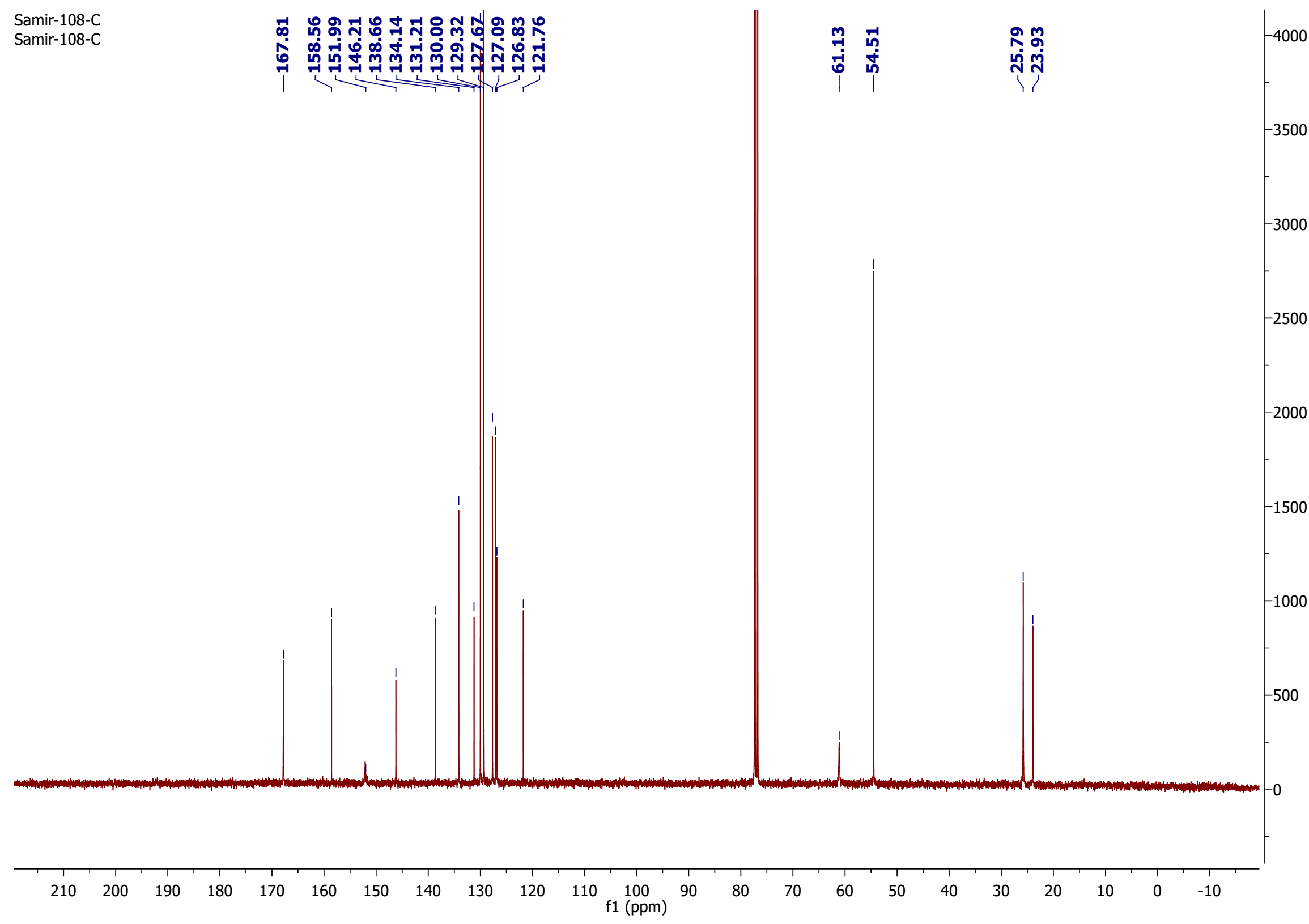


^13^C-NMR spectrum of compound **22**

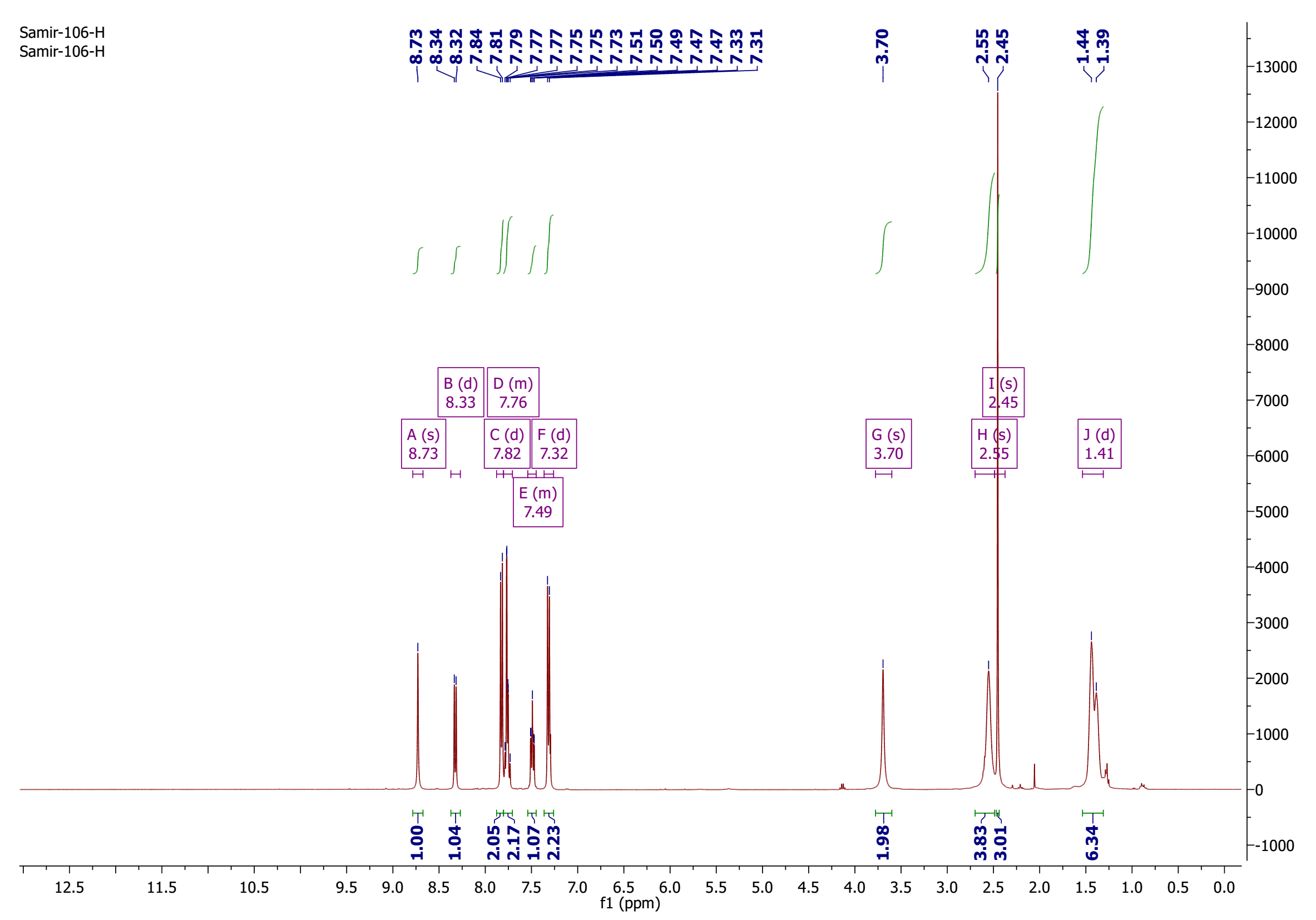


^1^H-NMR spectrum of compound **23**


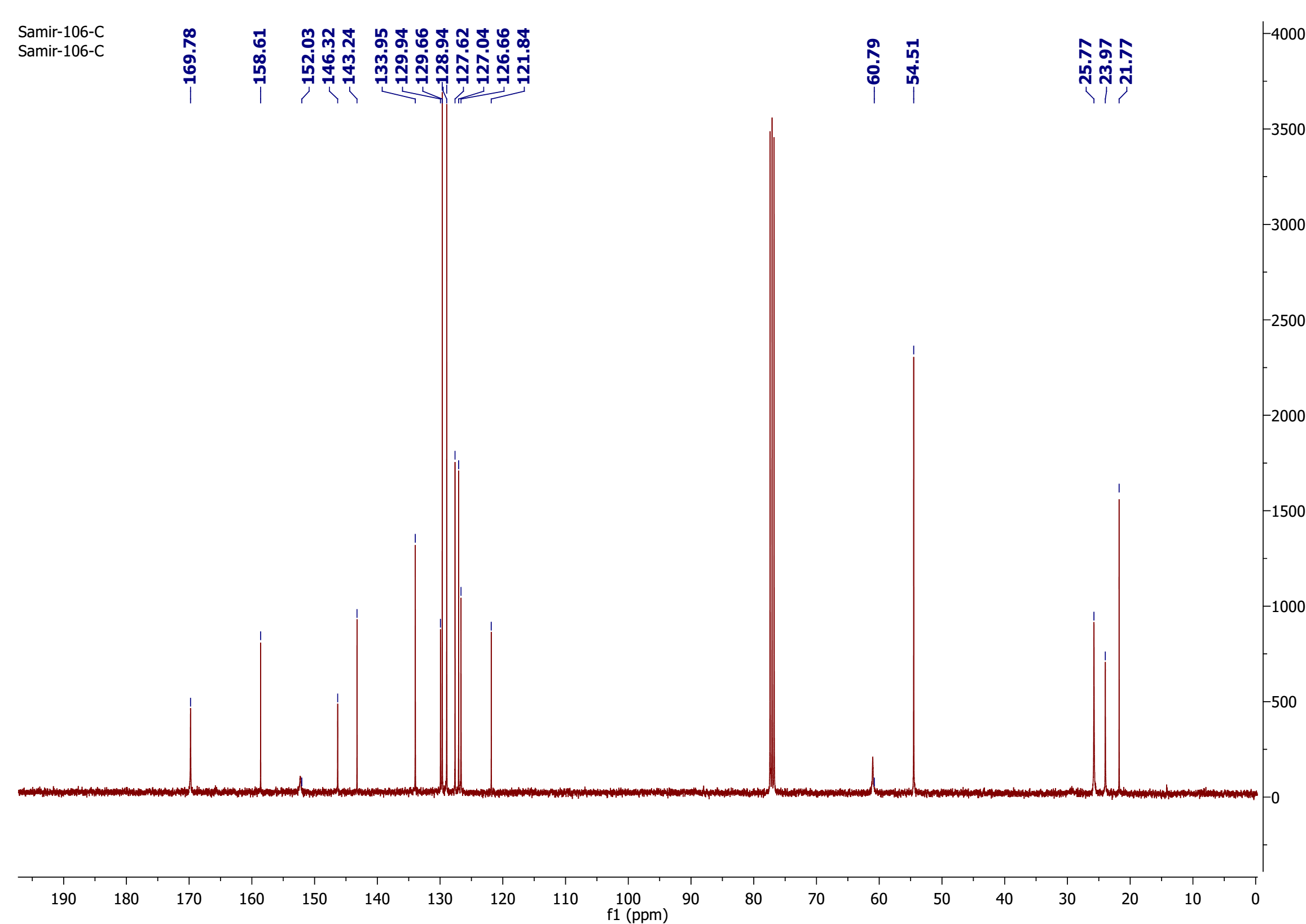


^13^C-NMR spectrum of compound **23**

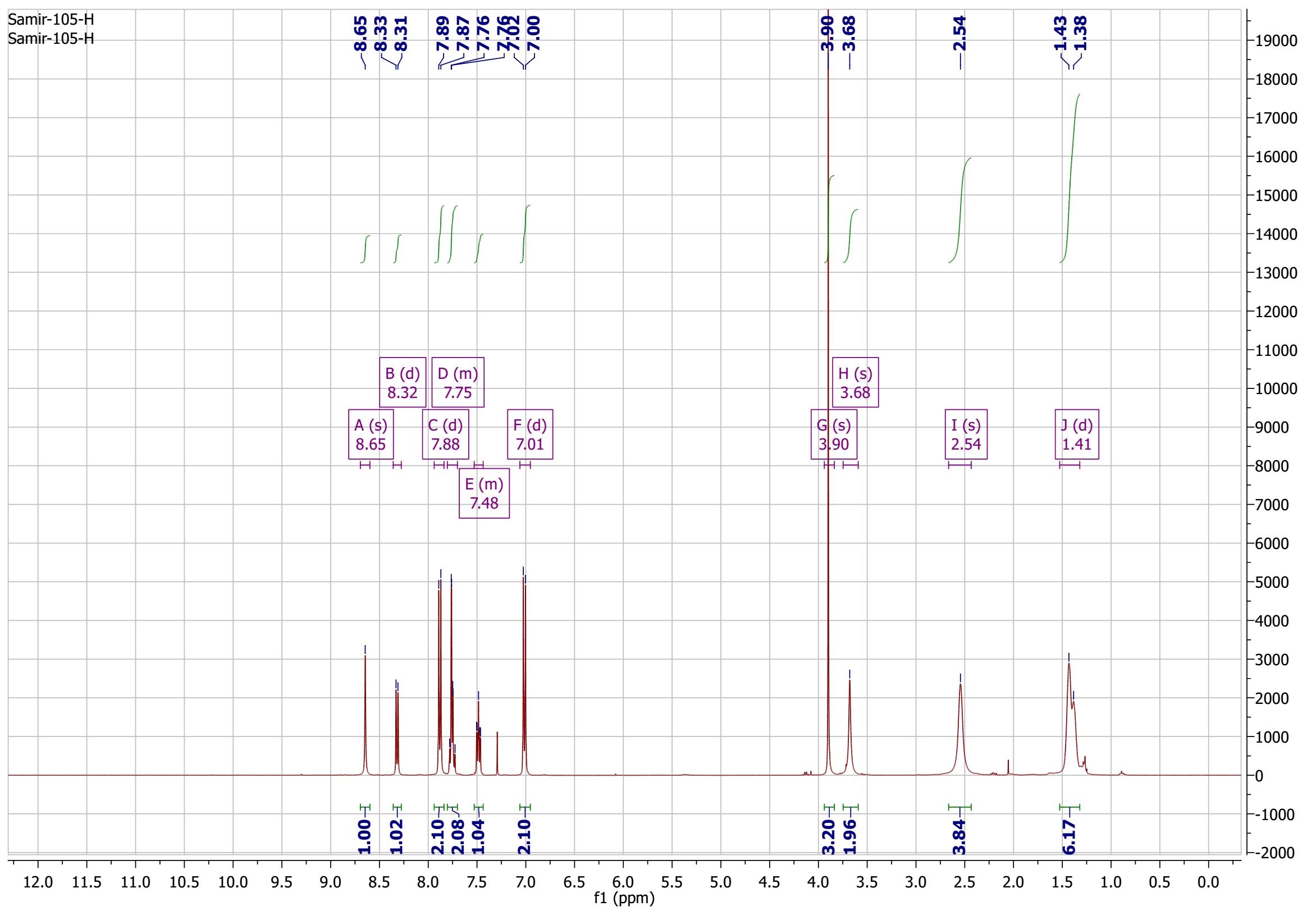


^1^H-NMR spectrum of compound **24**


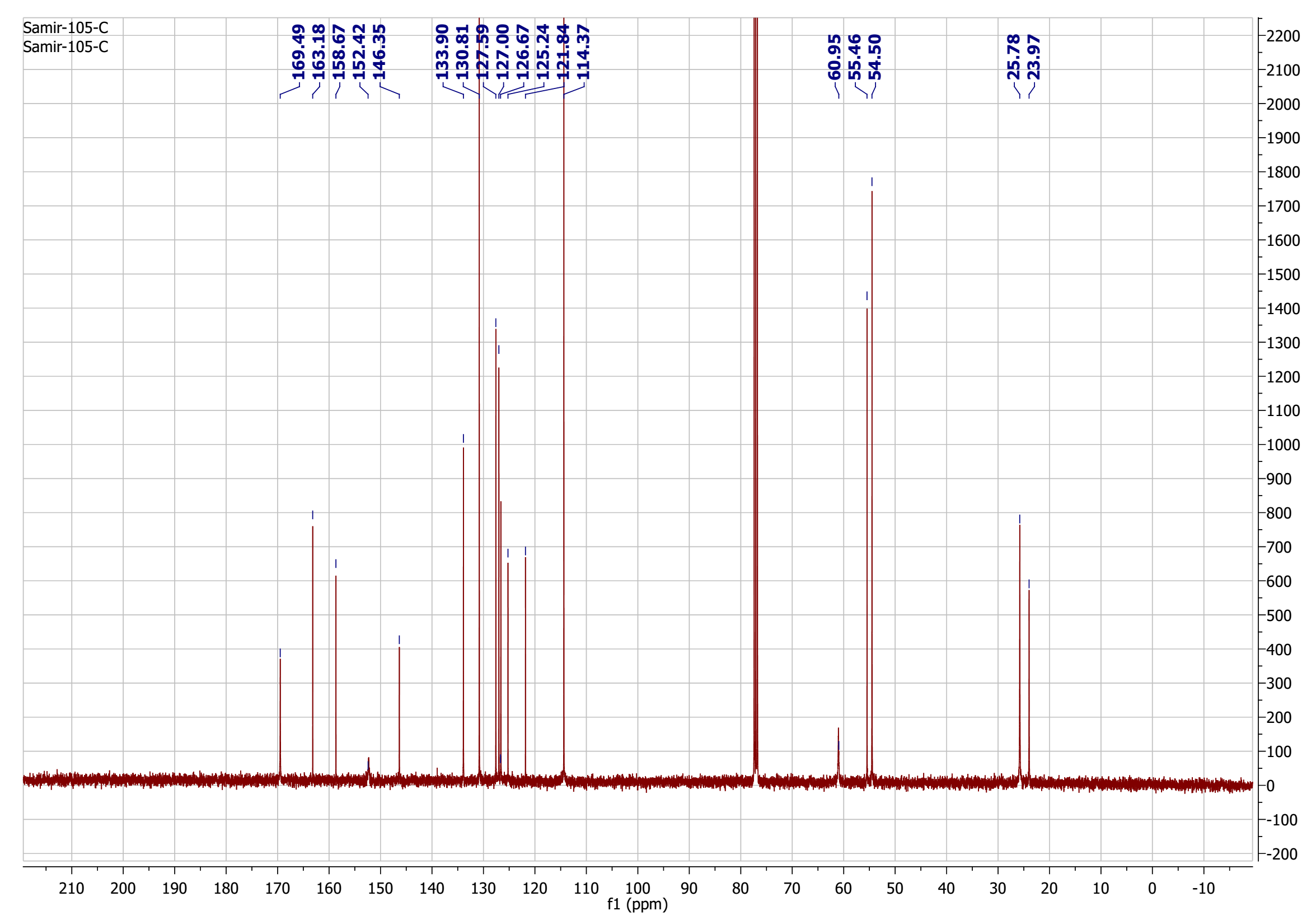


^13^C-NMR spectrum of compound **24**

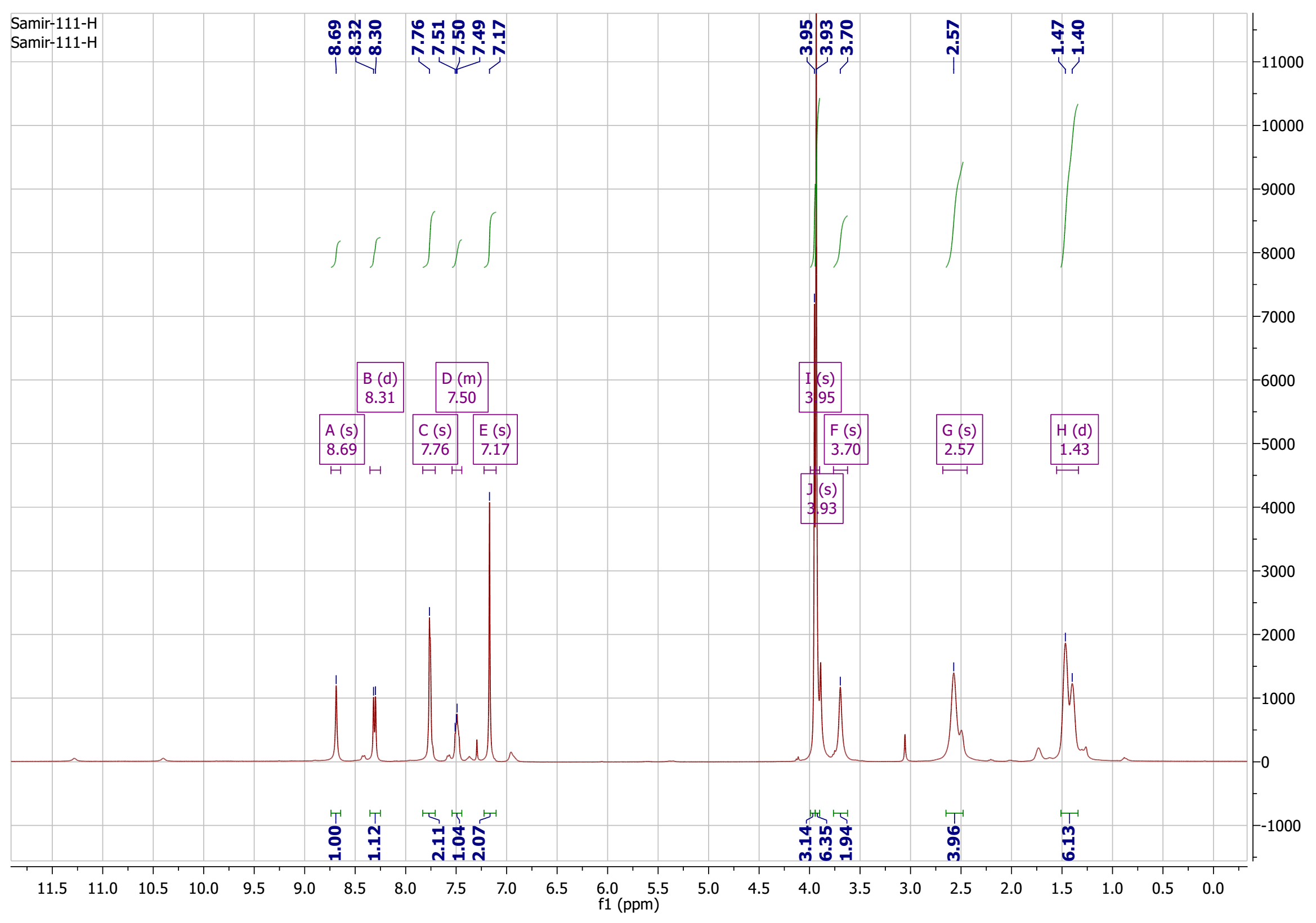


^1^H-NMR spectrum of compound **25**


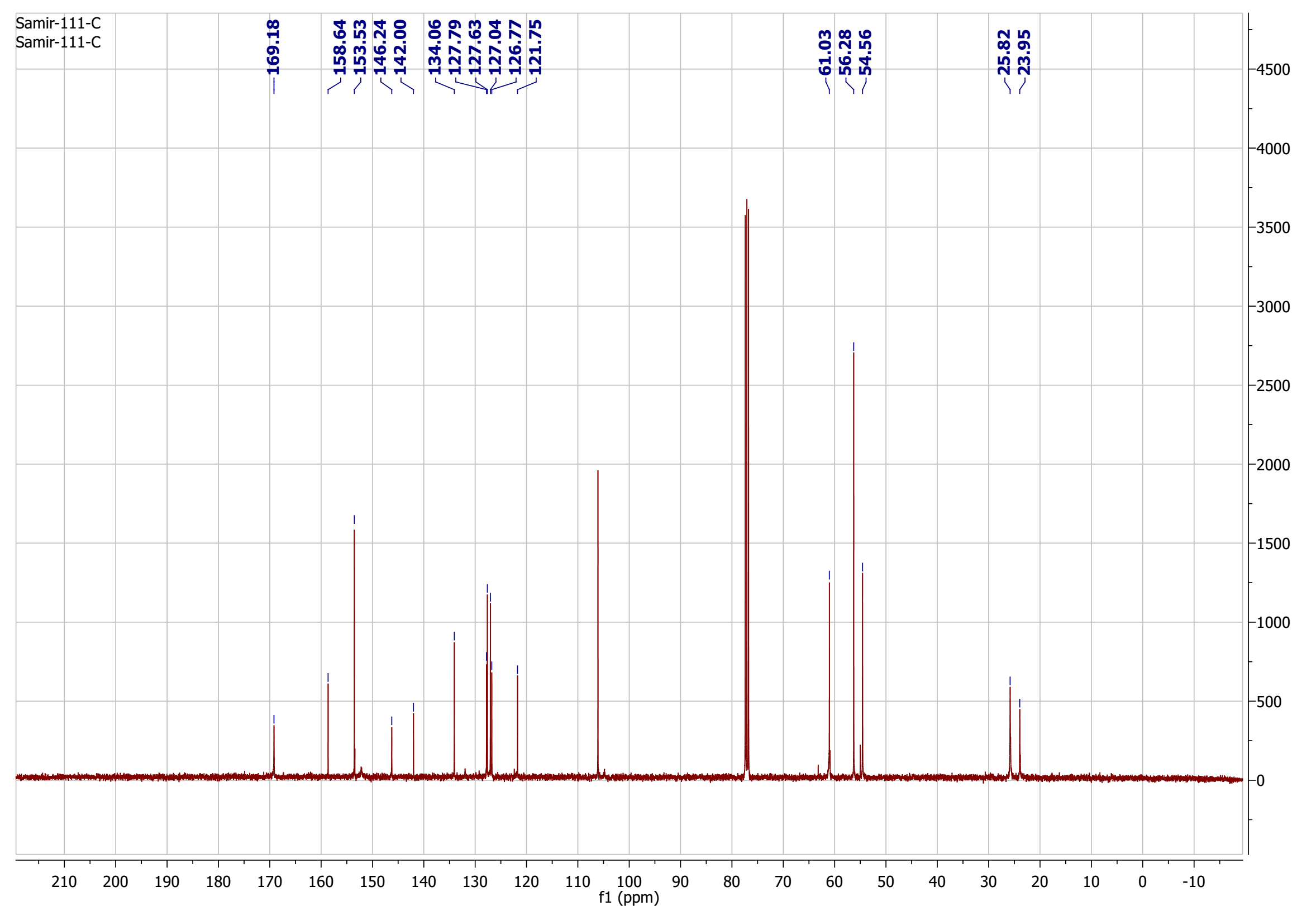


^13^C-NMR spectrum of compound **25**

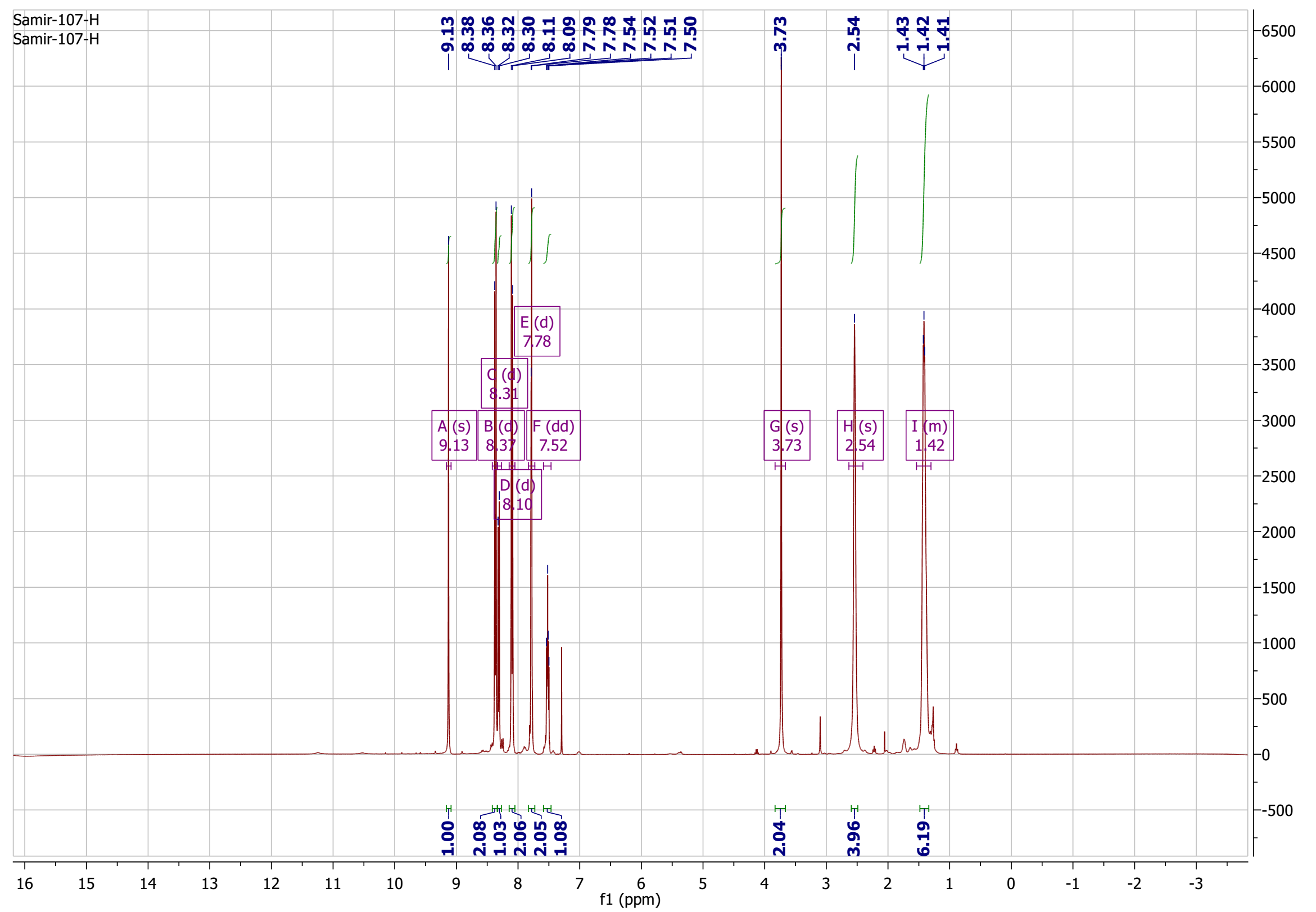


^1^H-NMR spectrum of compound **26**


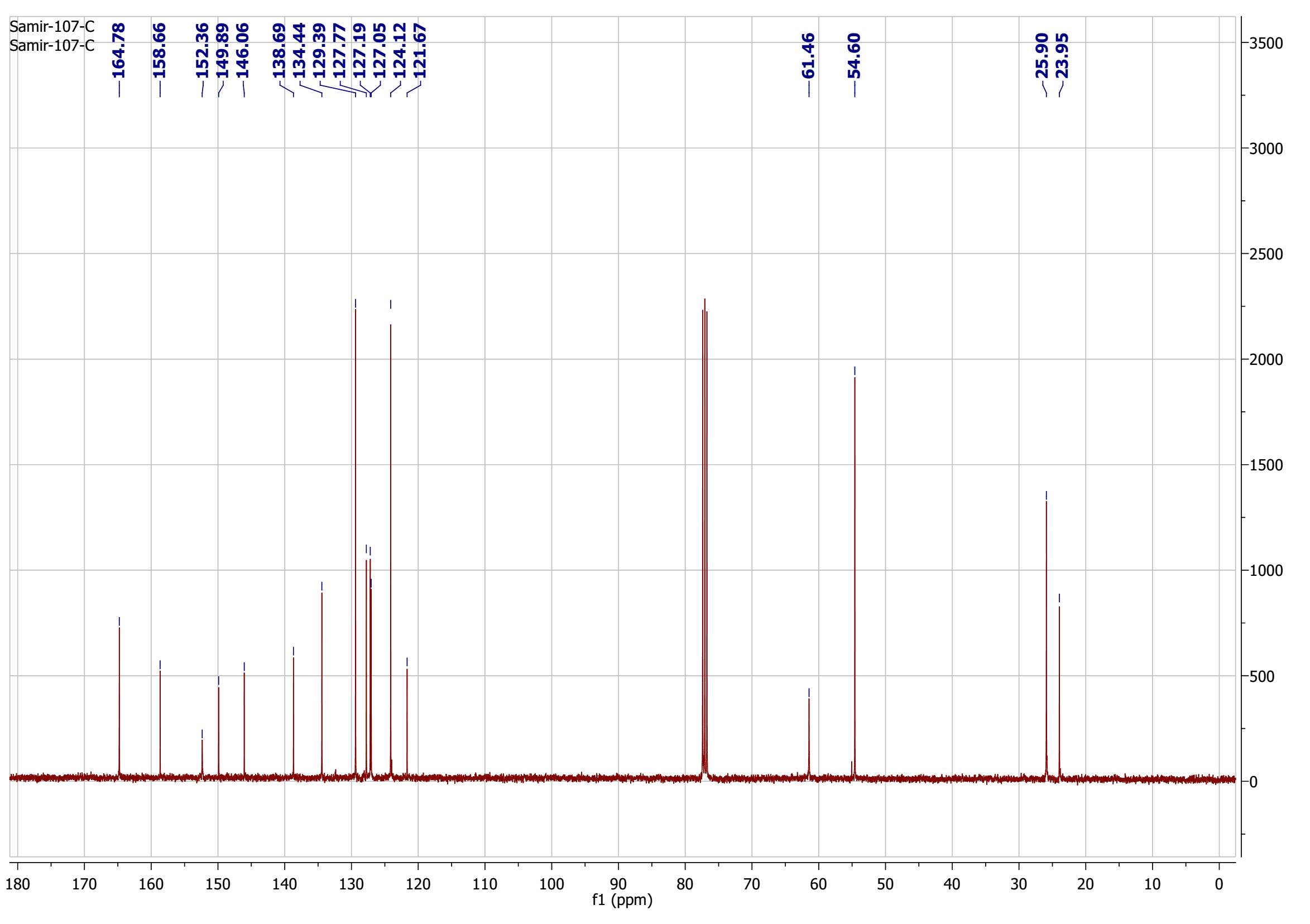


^13^C-NMR spectrum of compound **26**

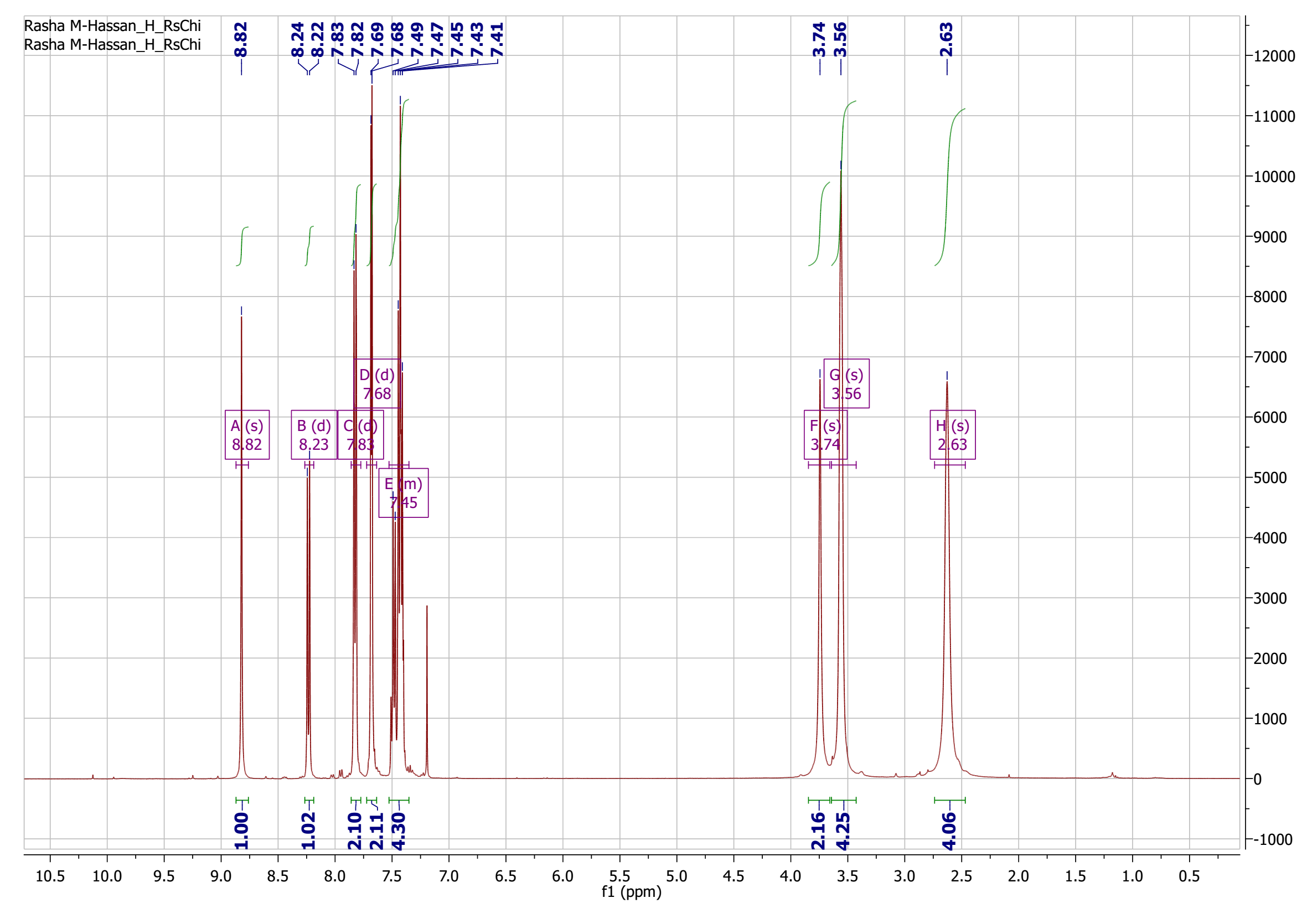


^1^H-NMR spectrum of compound **27**


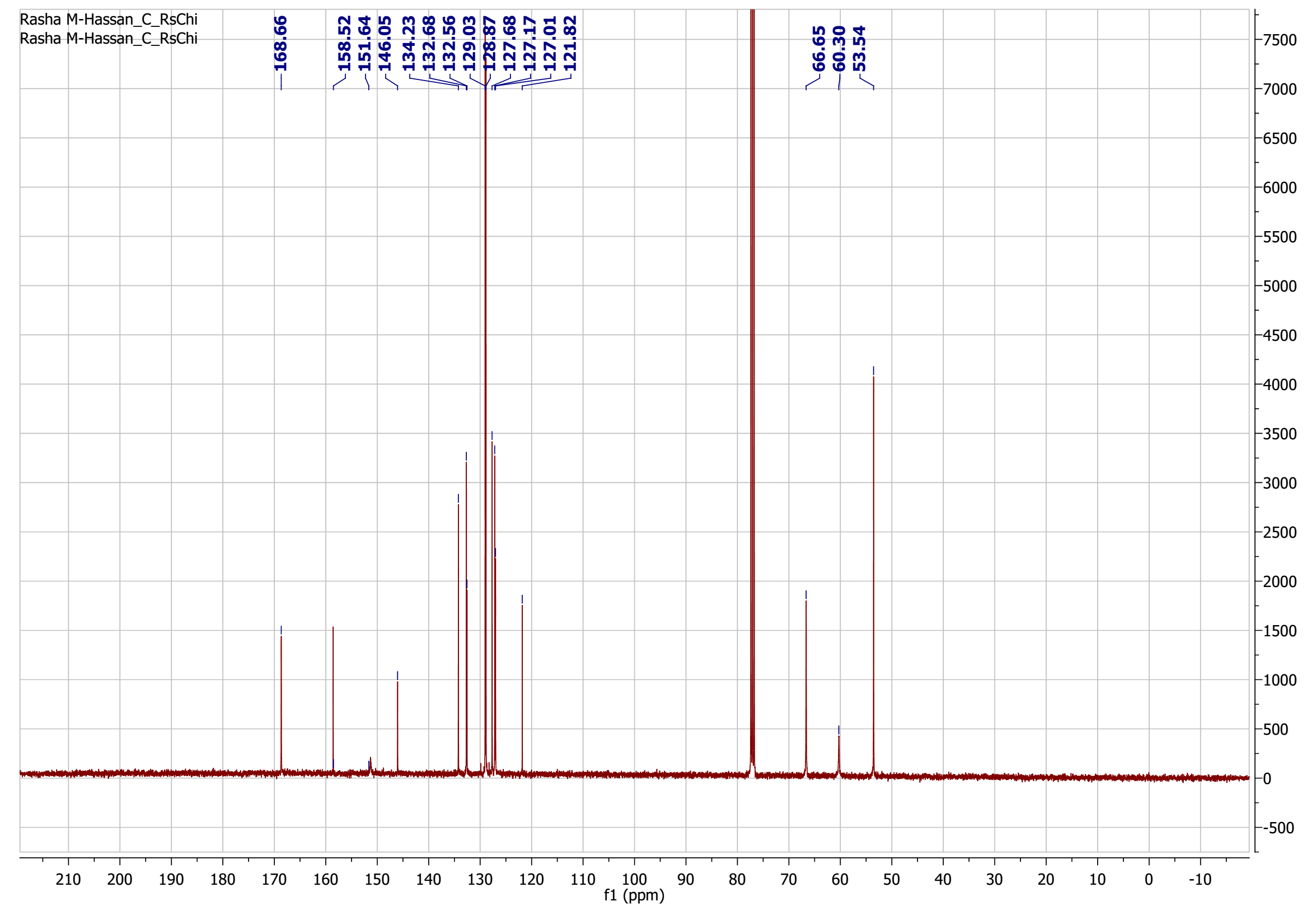


^13^C-NMR spectrum of compound **27**

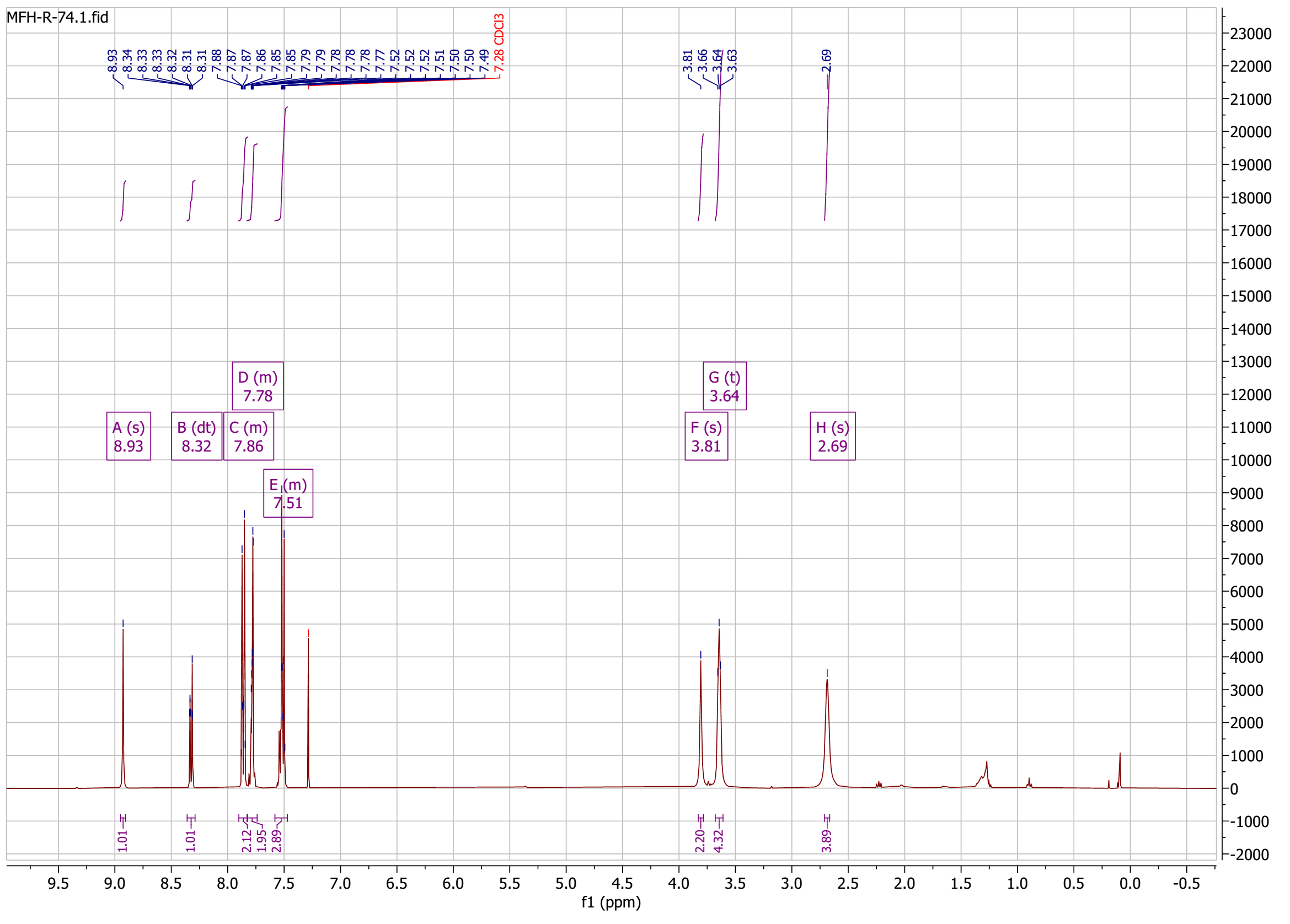


^1^H-NMR spectrum of compound **28**


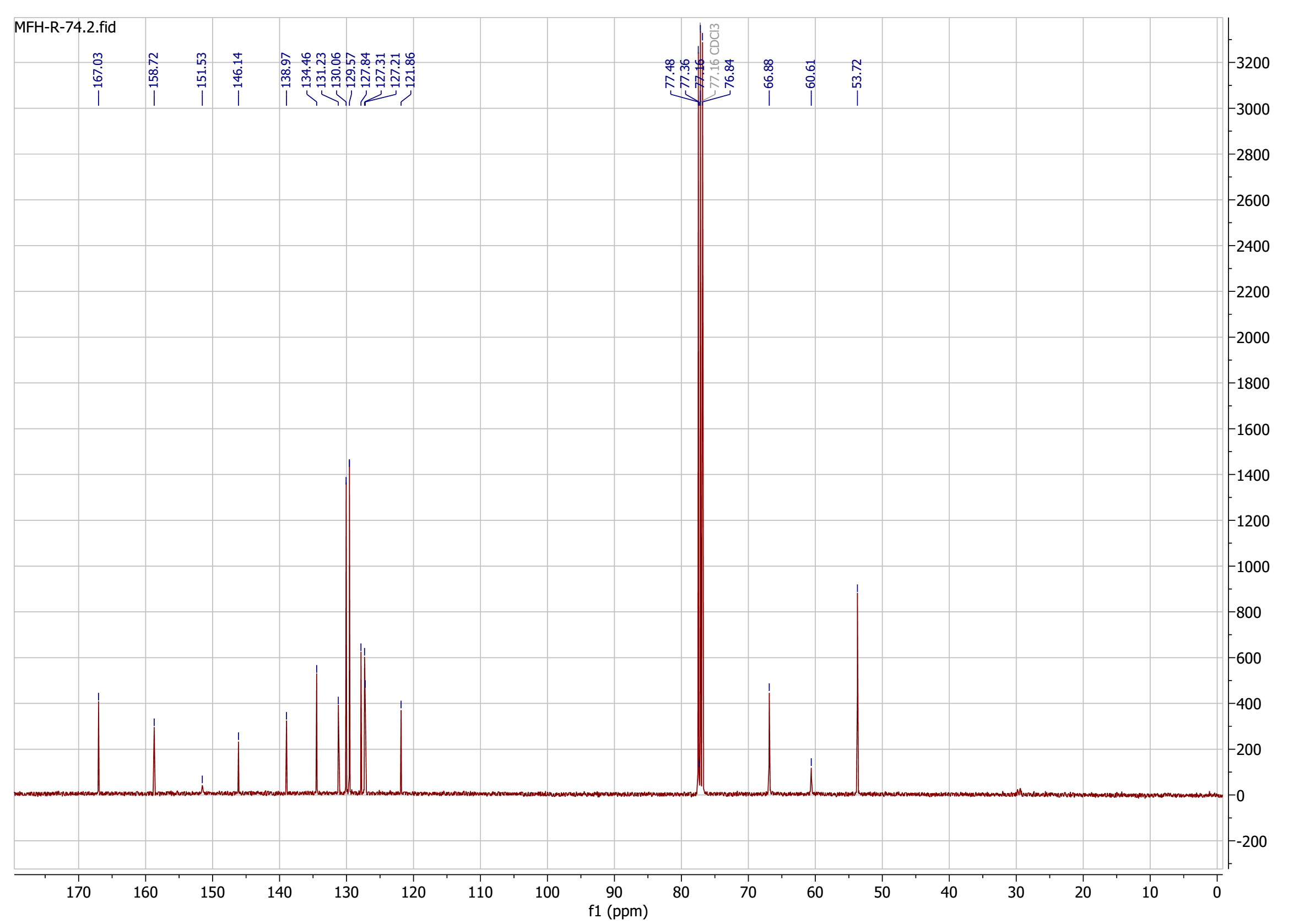


^13^C-NMR spectrum of compound **28**

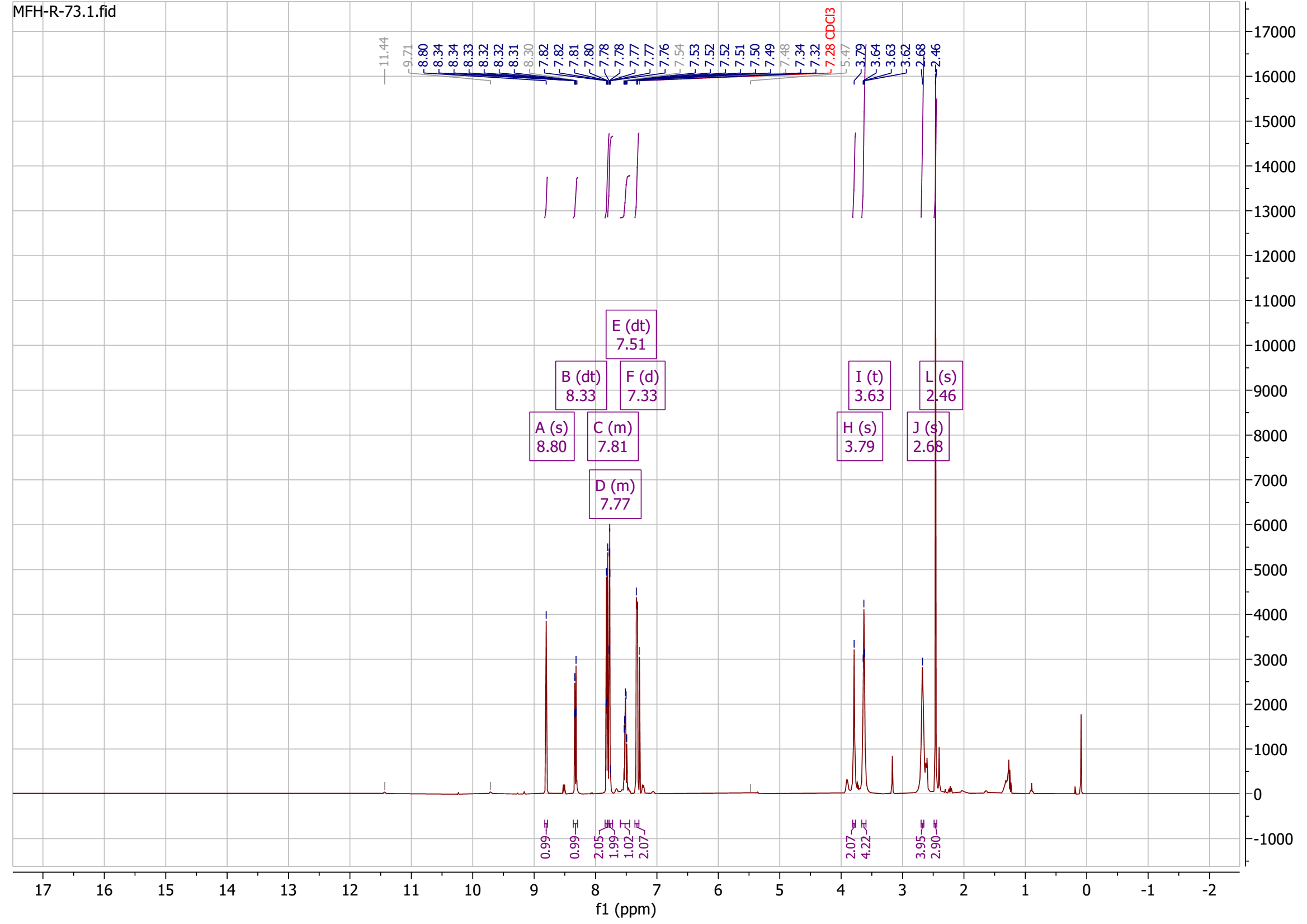


^1^H-NMR spectrum of compound **29**


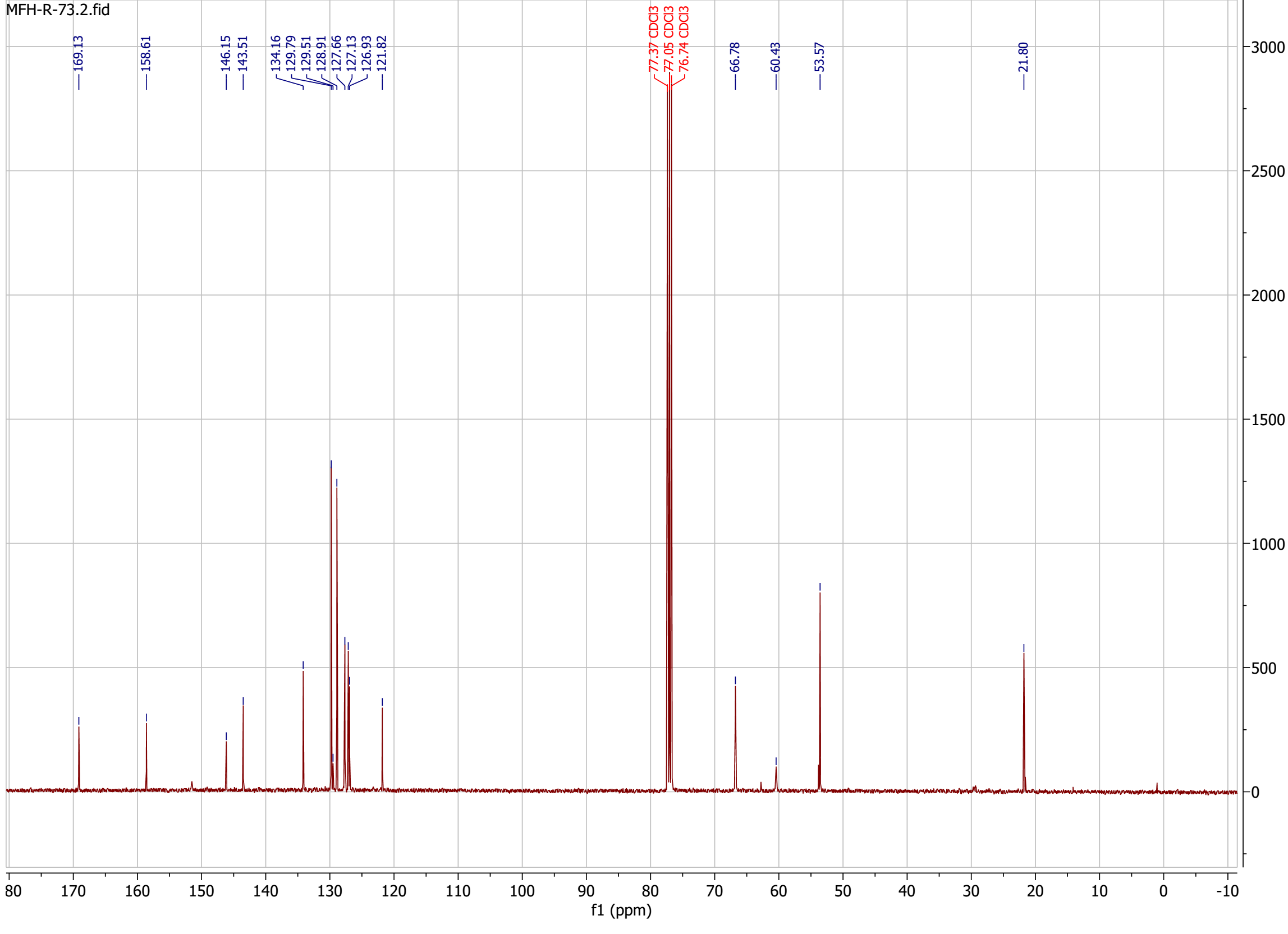


^13^C-NMR spectrum of compound **29**

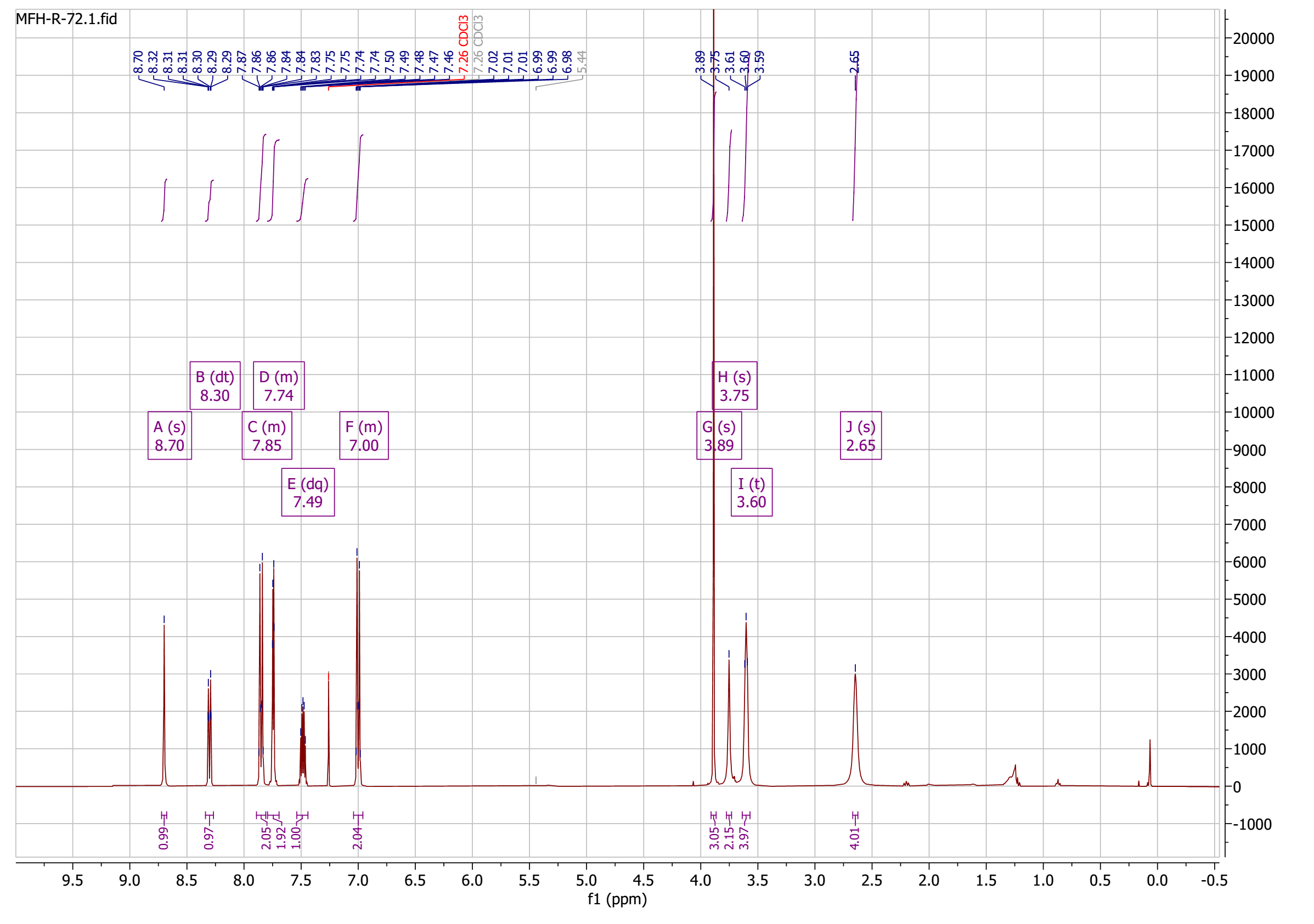


^1^H-NMR spectrum of compound **30**


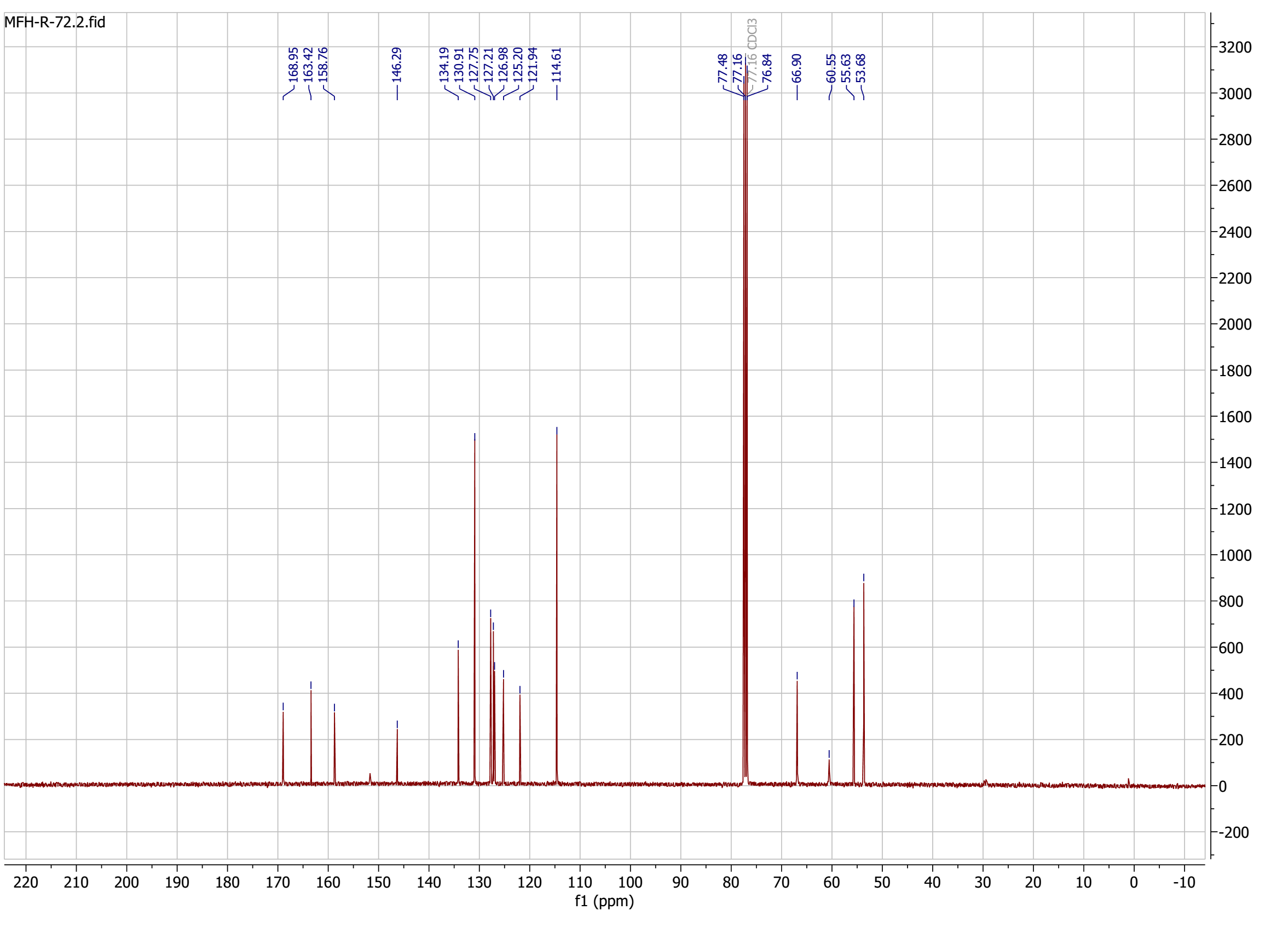


^13^C-NMR spectrum of compound **30**

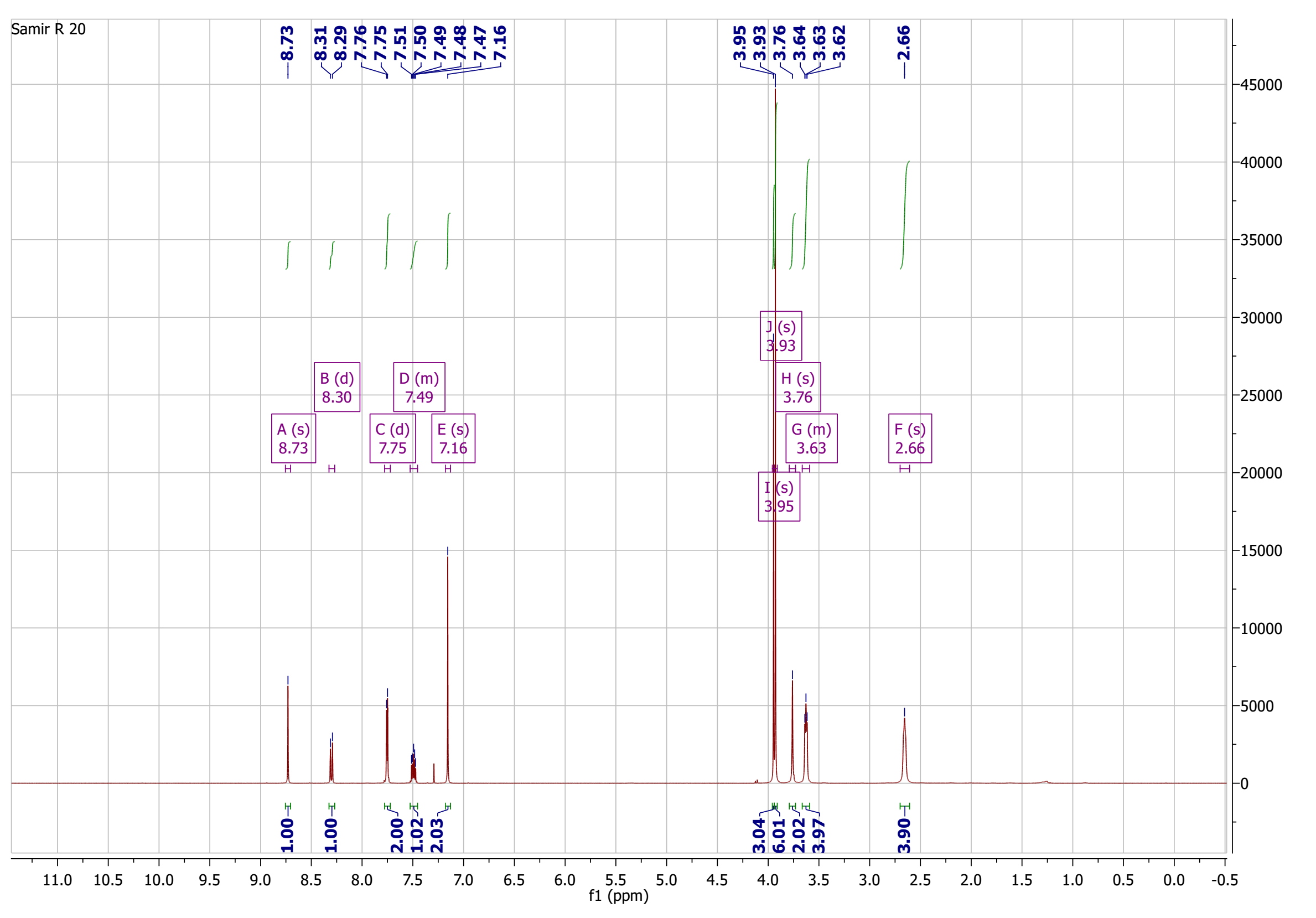


^1^H-NMR spectrum of compound **31**


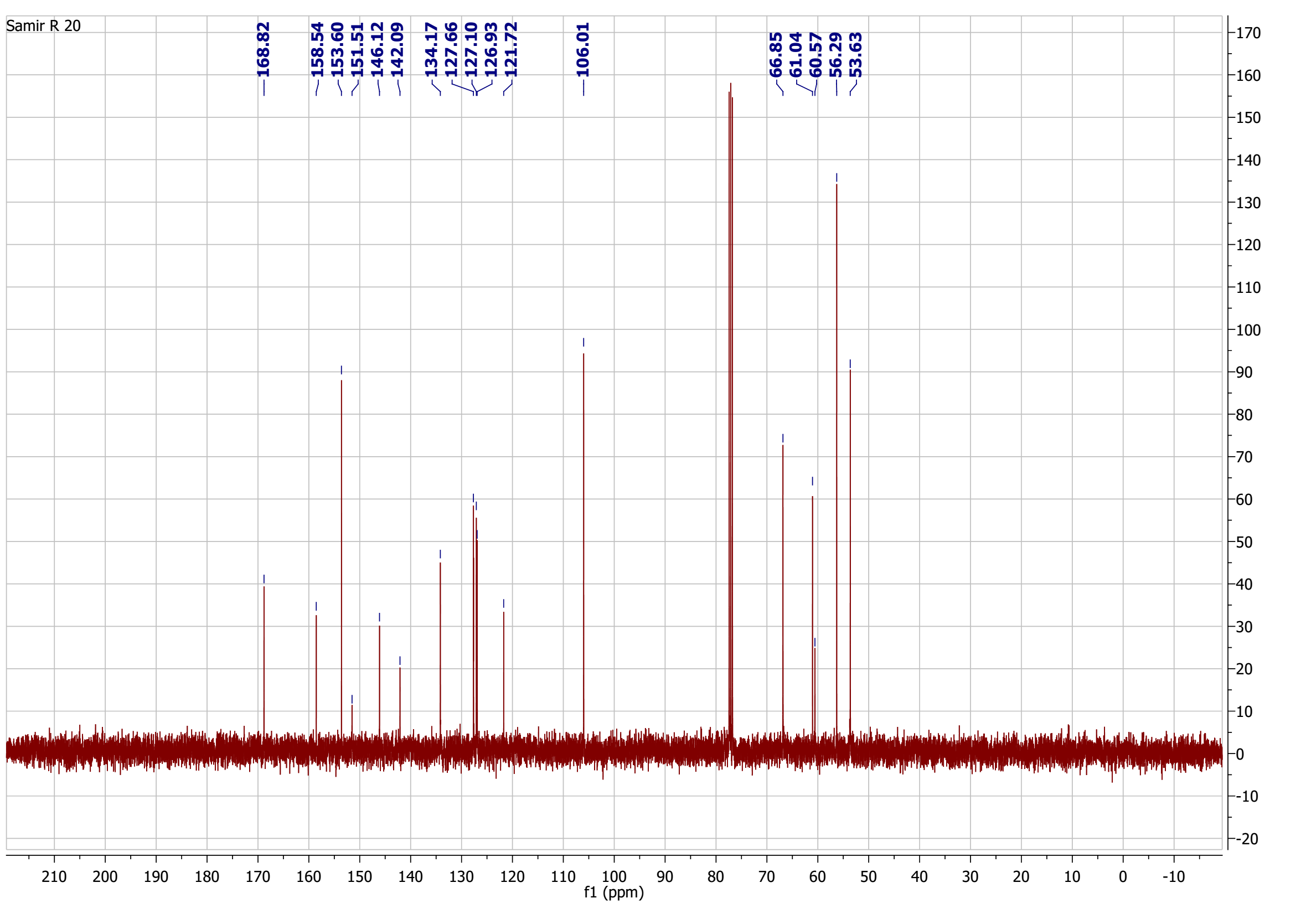


^13^C-NMR spectrum of compound **31**

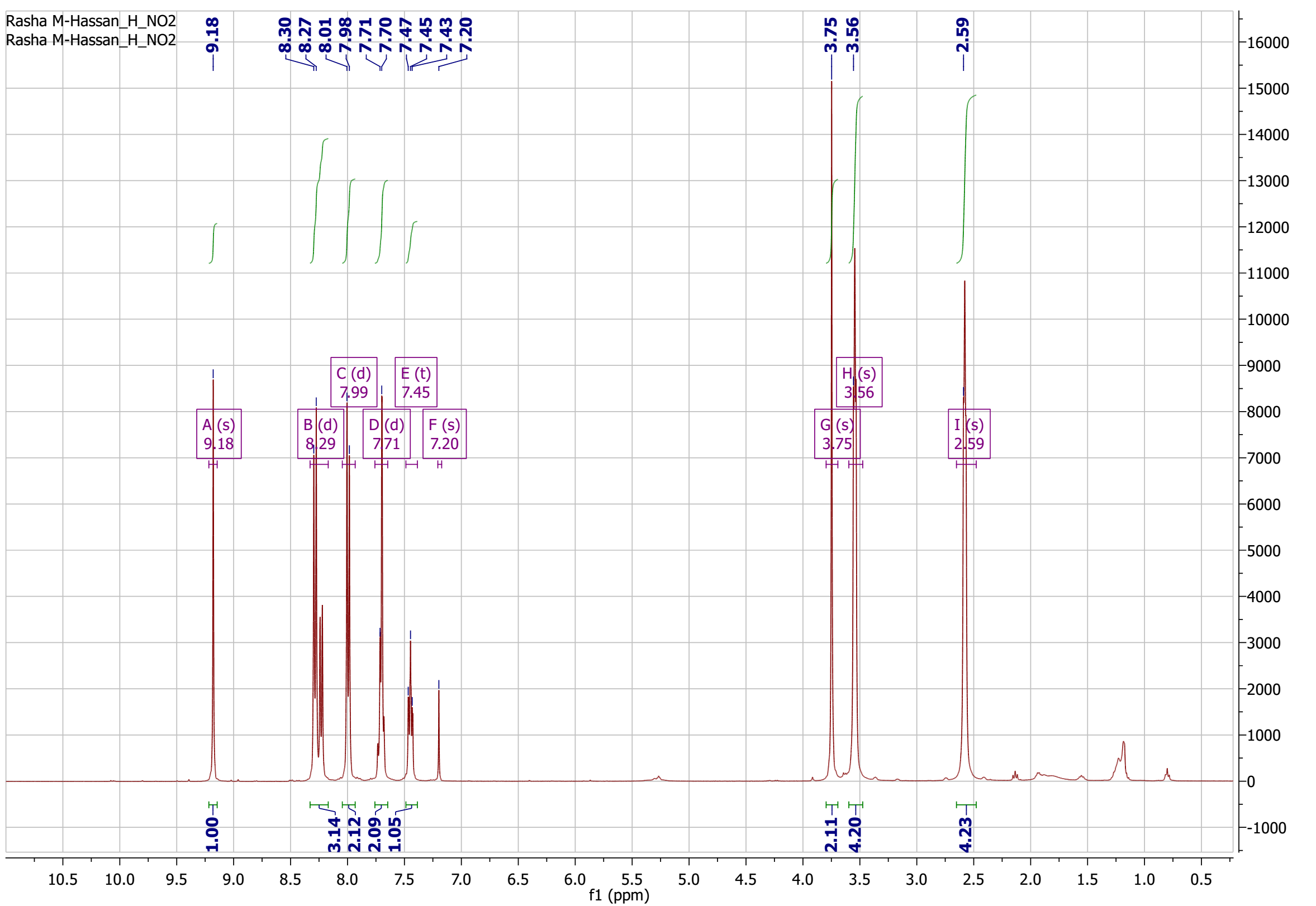


^1^H-NMR spectrum of compound **32**


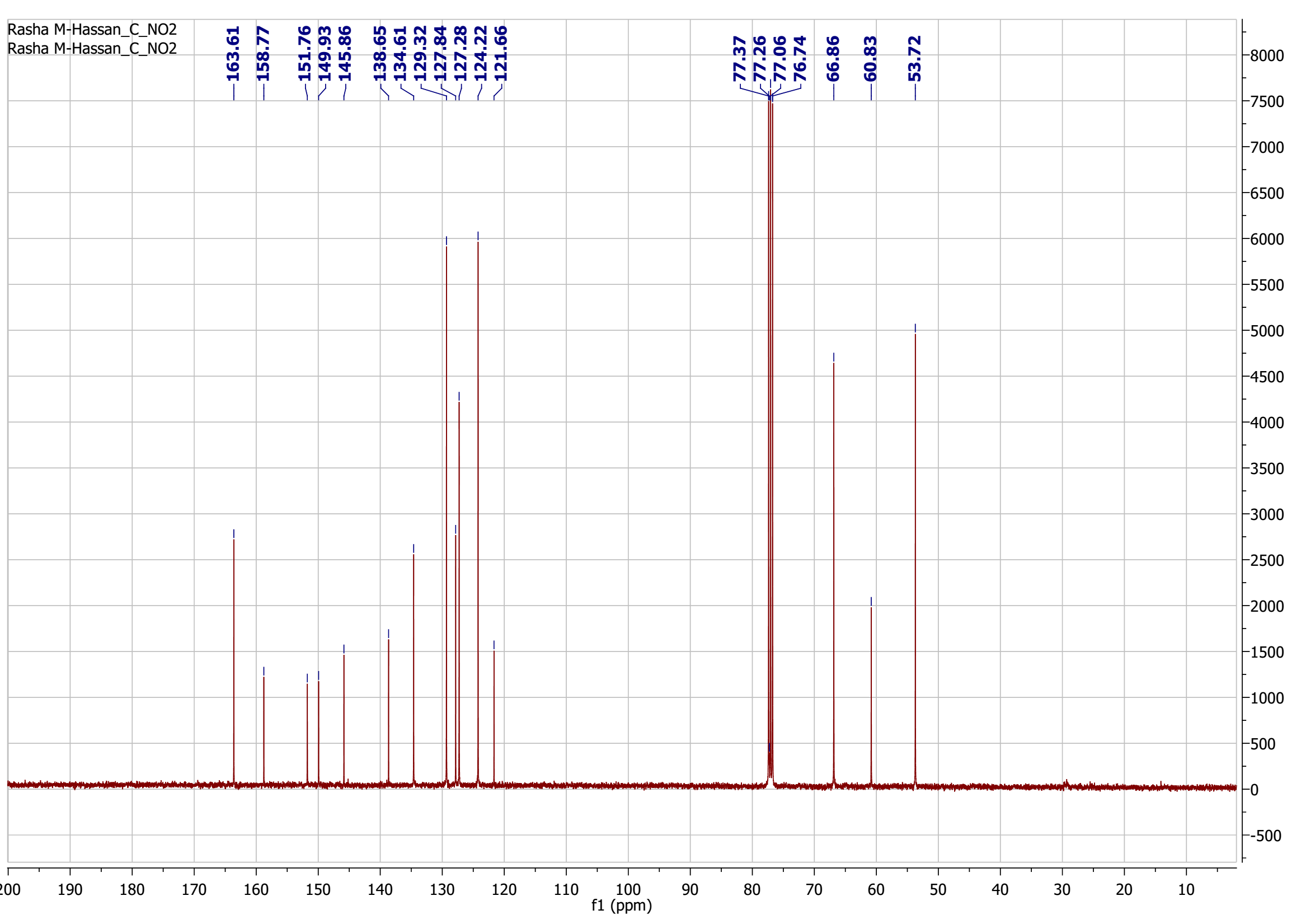


^13^C-NMR spectrum of compound **32**


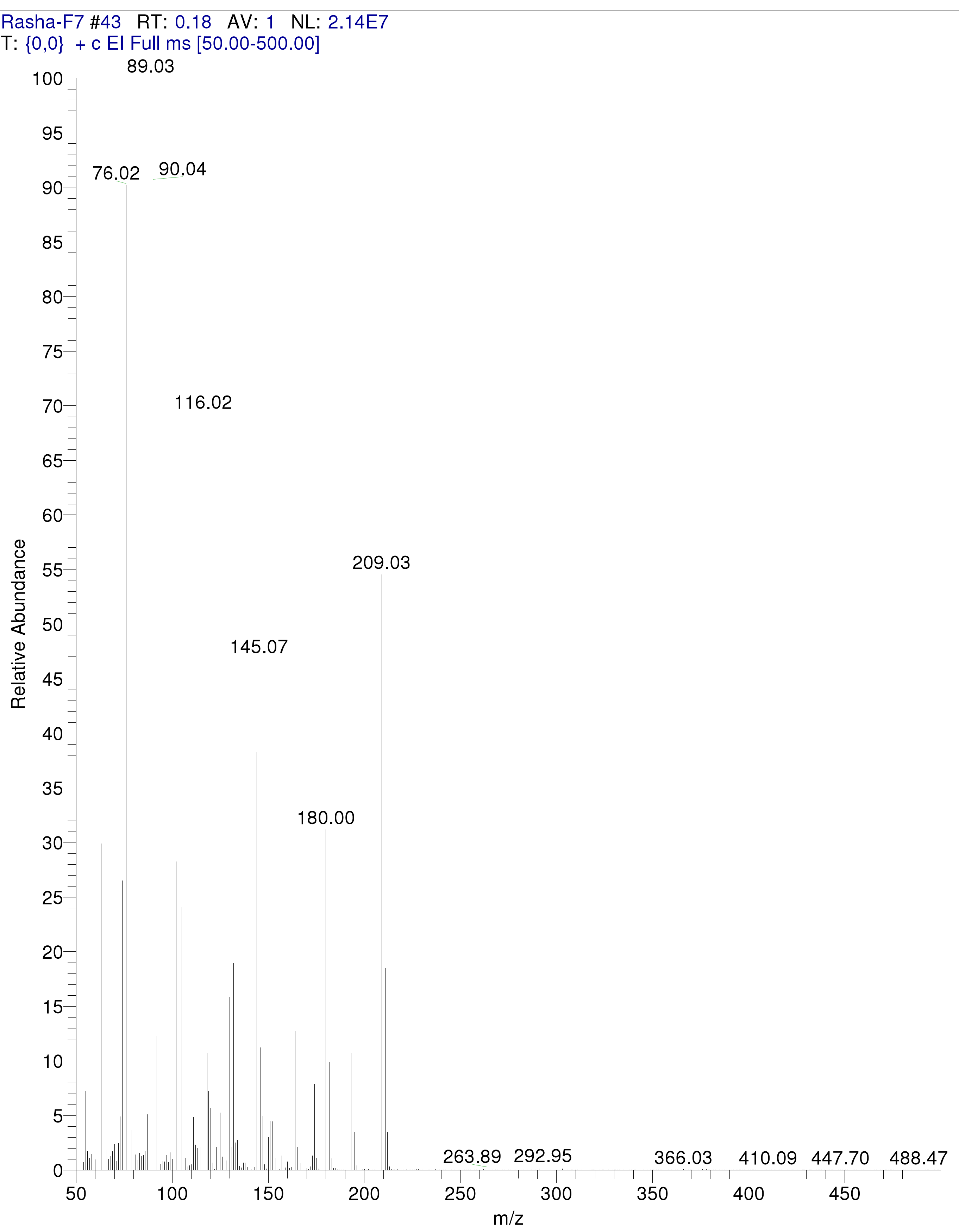
Mass spectrum for compound **8**


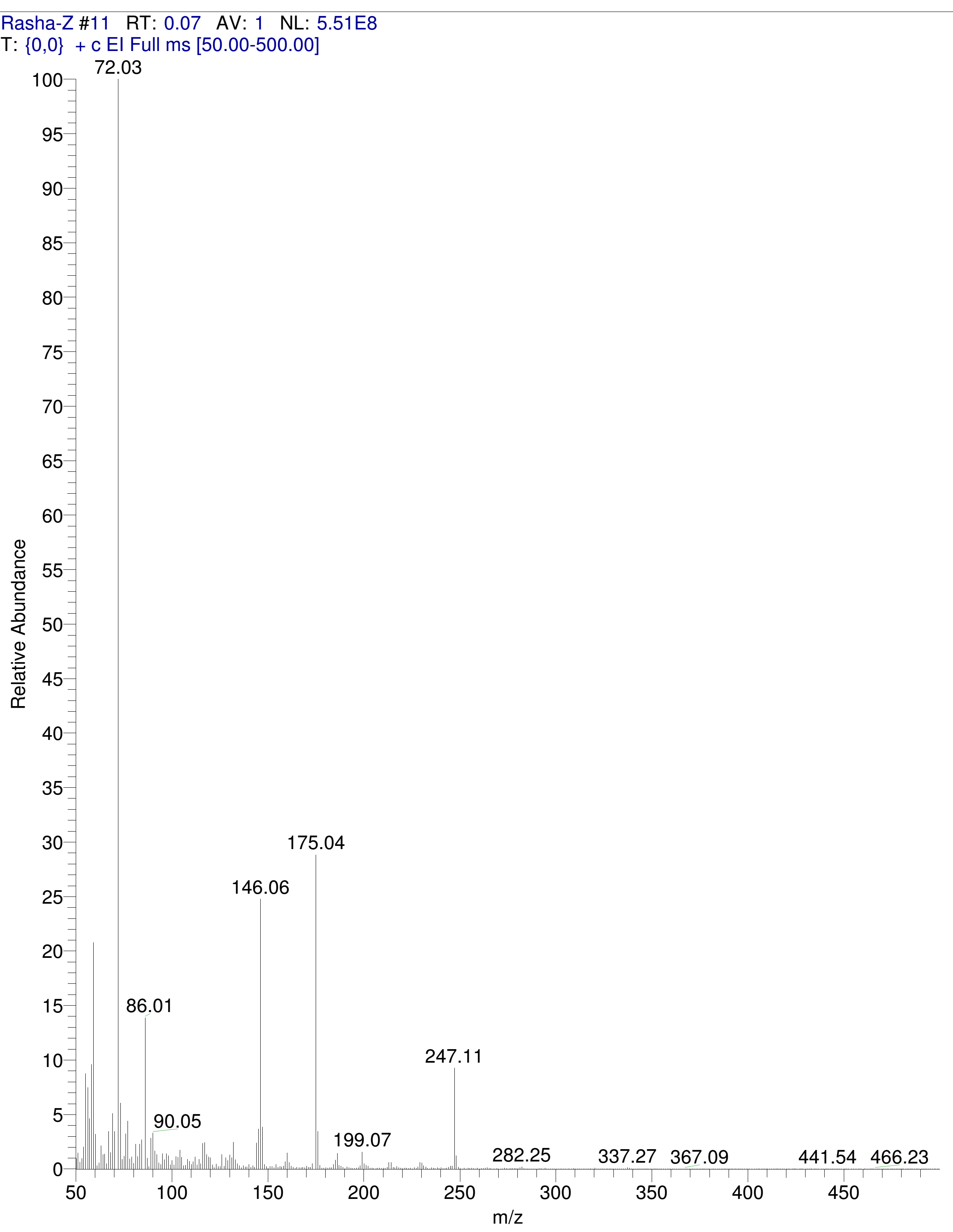


Mass spectrum for compound **5a**


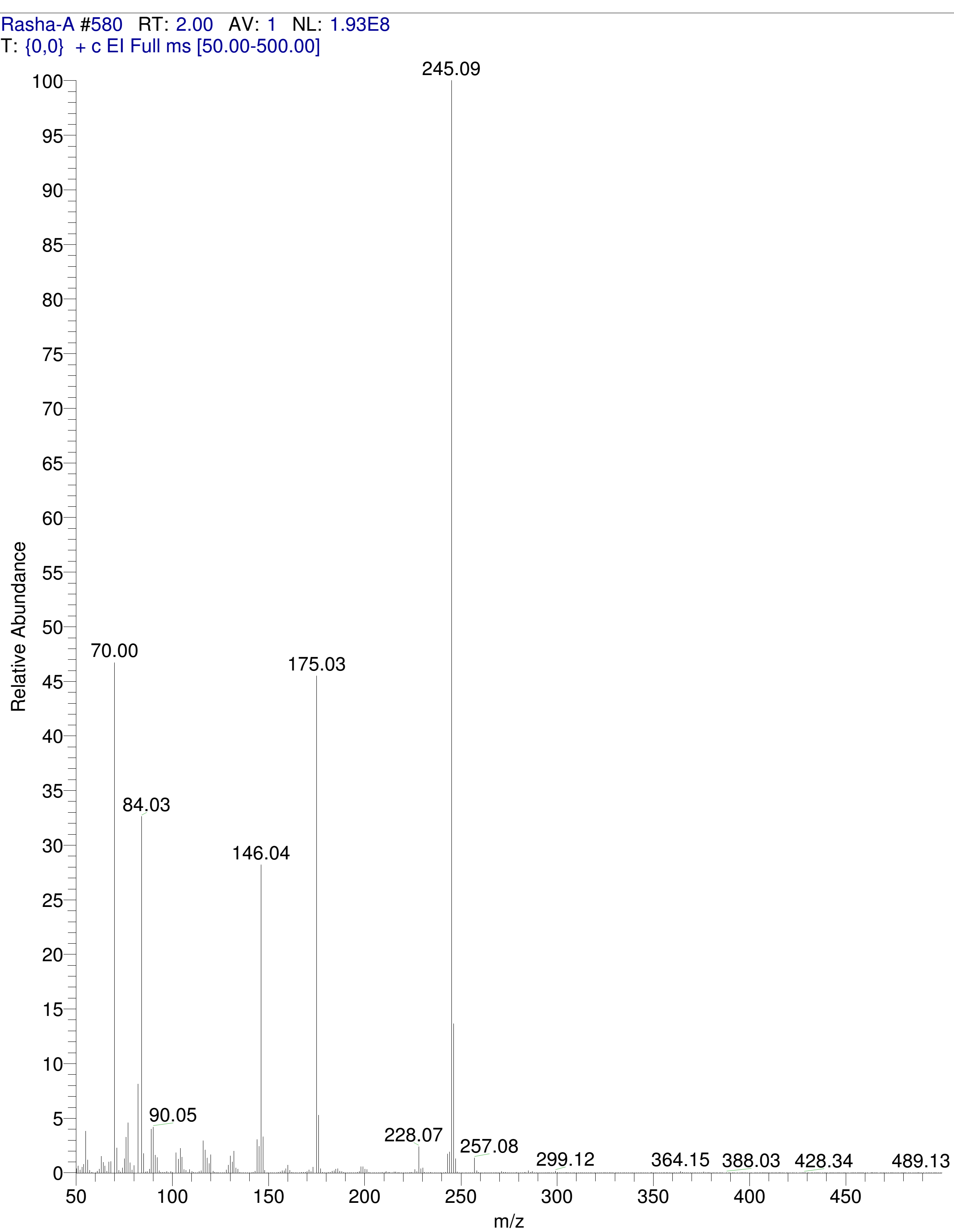
Mass spectrum for compound **5b**


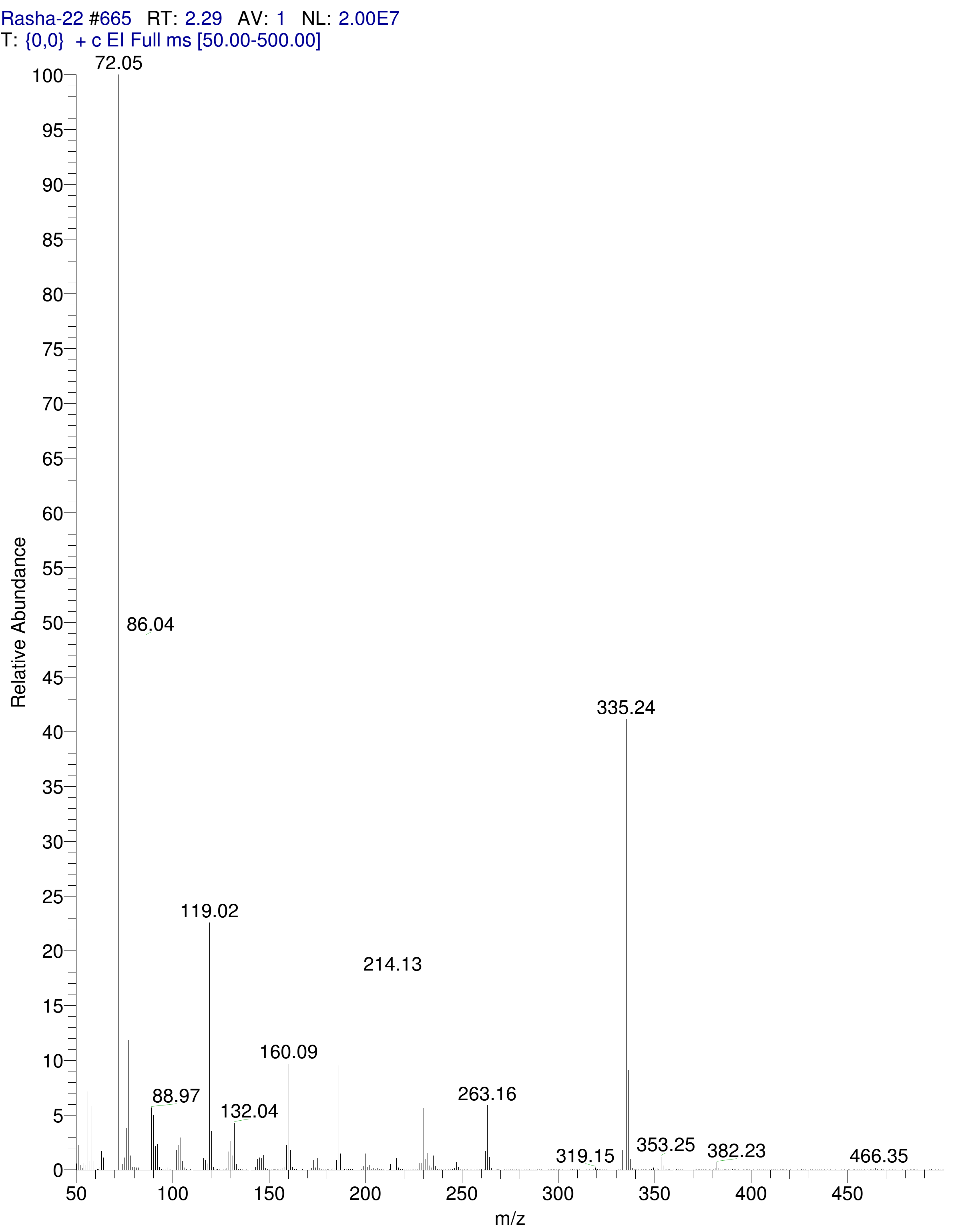


Mass spectrum for compound **9**


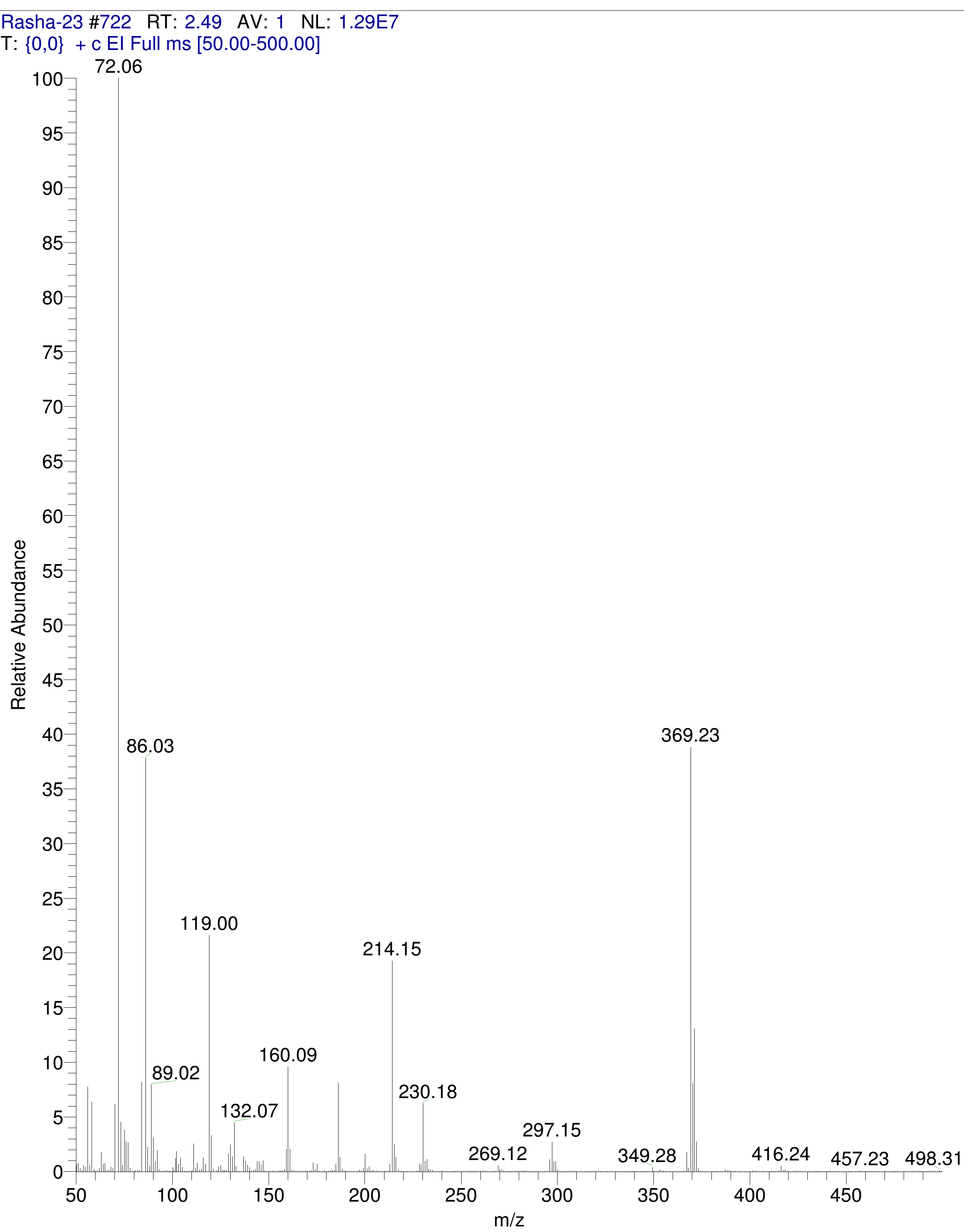


Mass spectrum for compound **10**


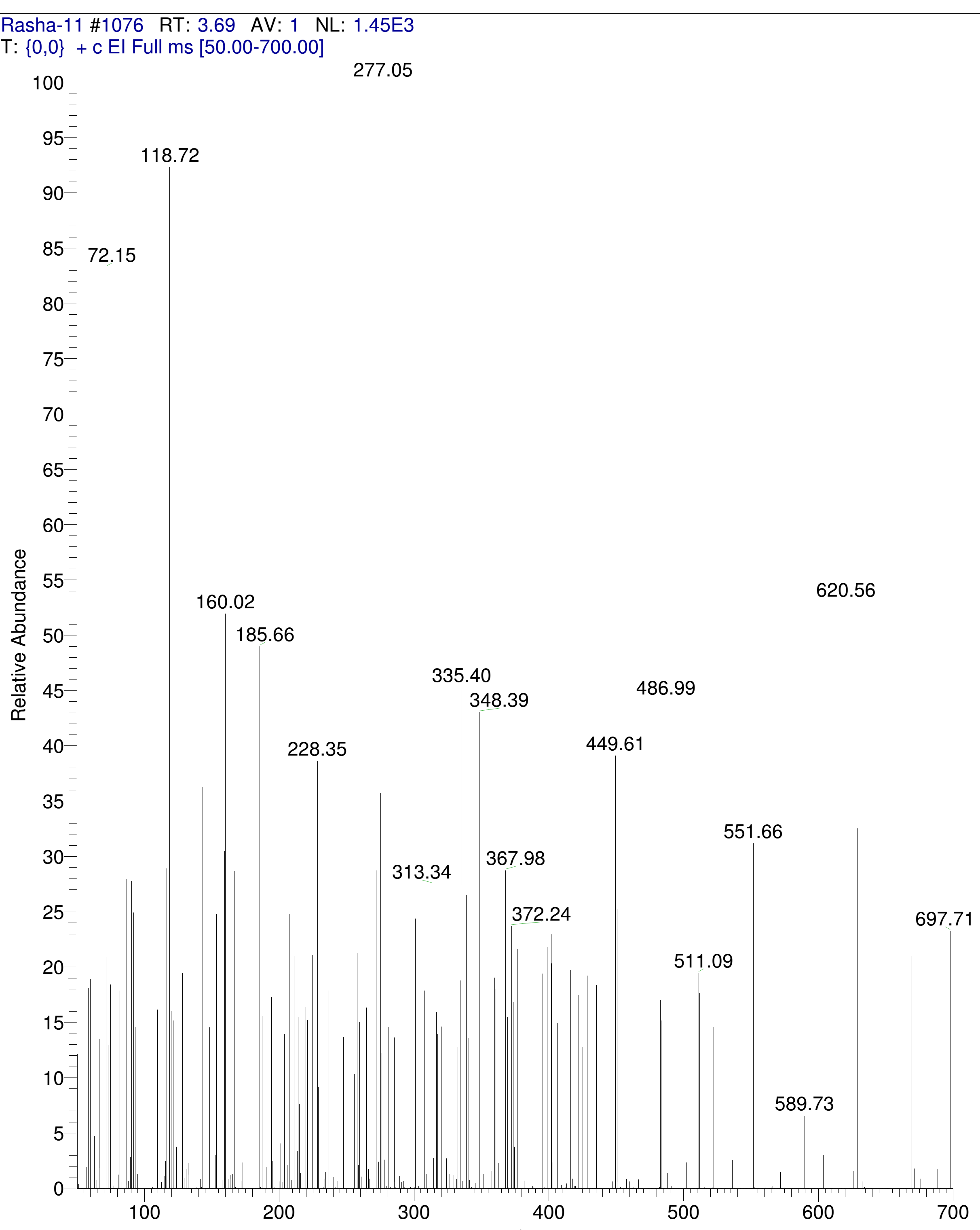


Mass spectrum for compound **11**


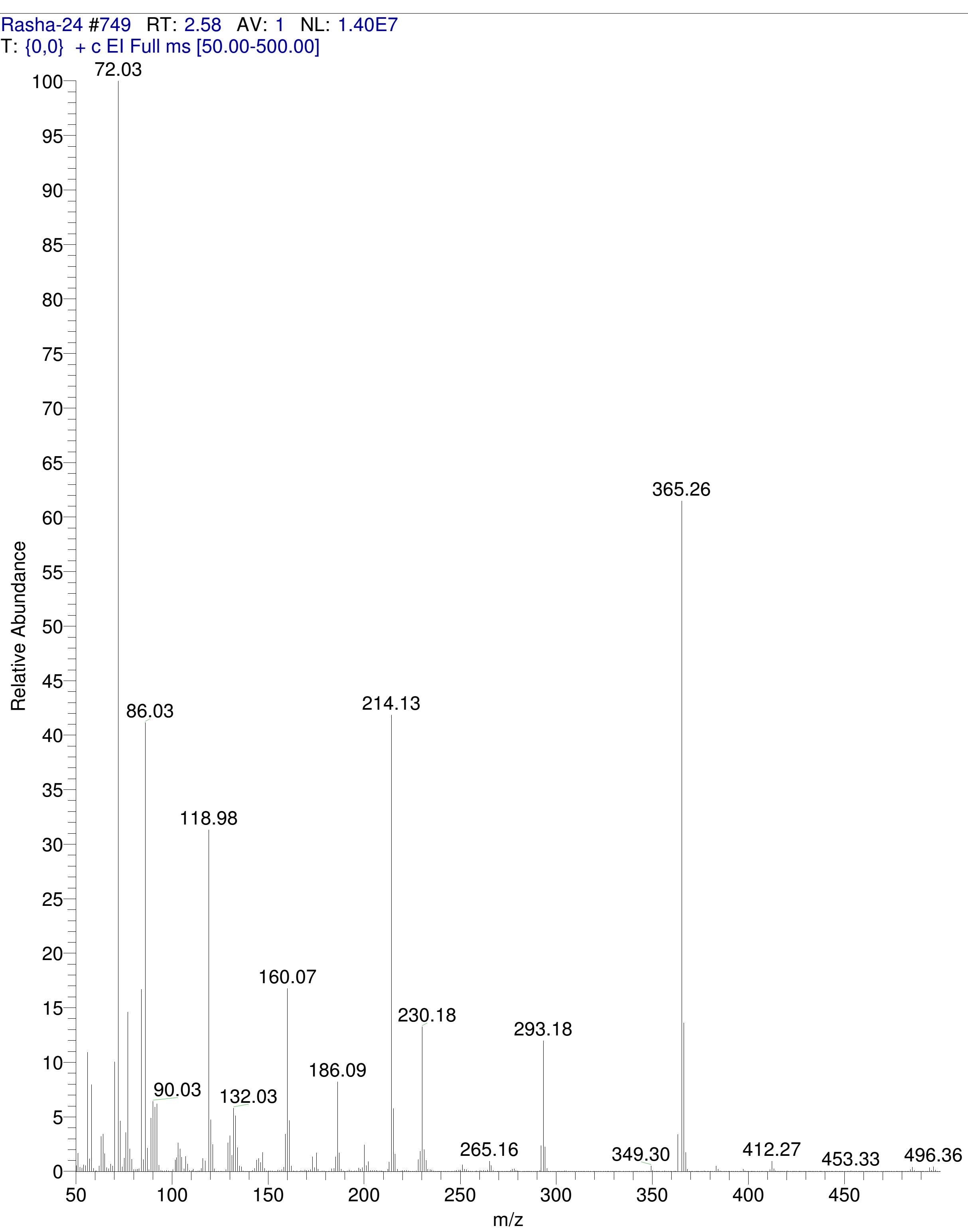


Mass spectrum for compound **12**


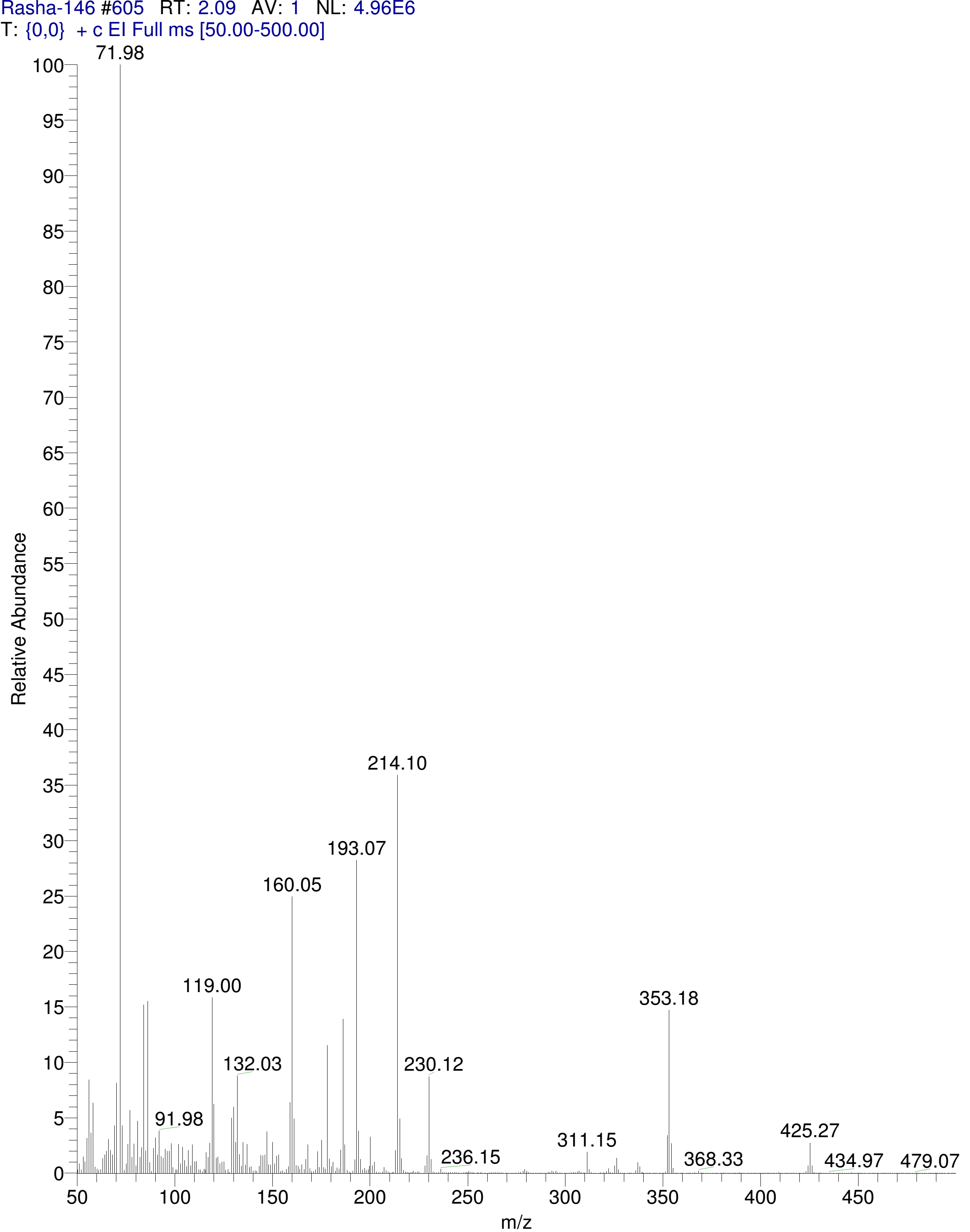


Mass spectrum for compound **13**


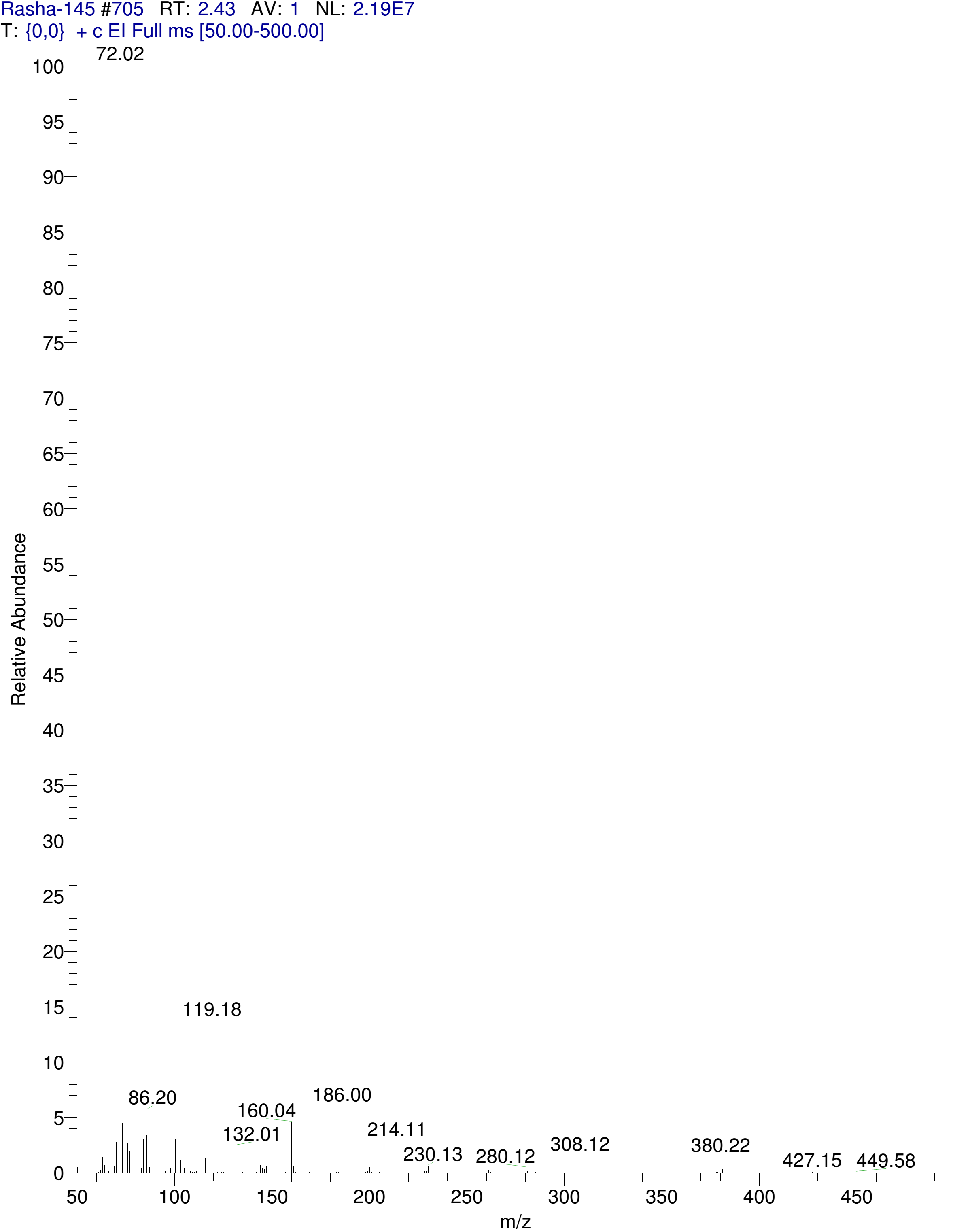


Mass spectrum for compound **14**


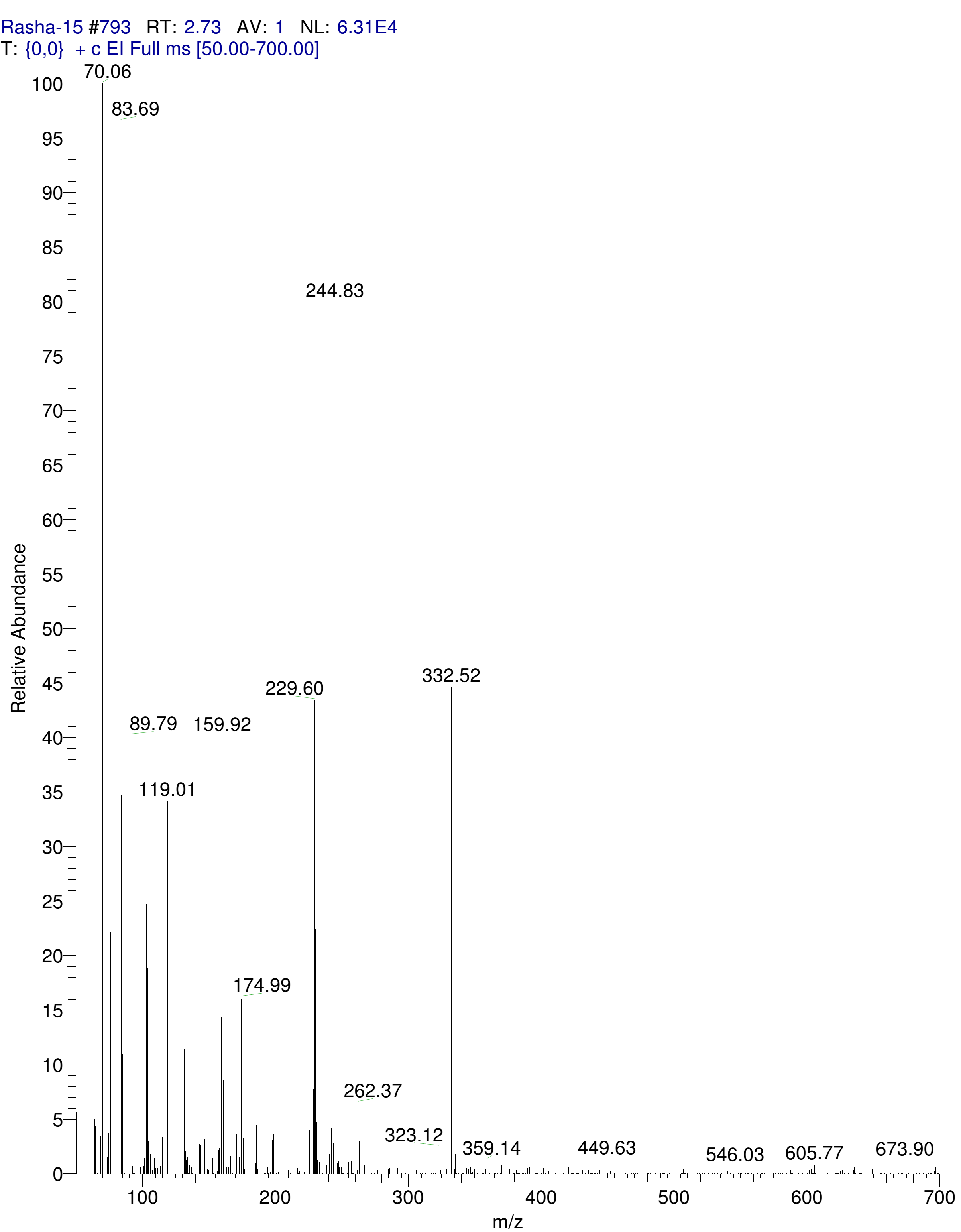


Mass spectrum for compound **15**


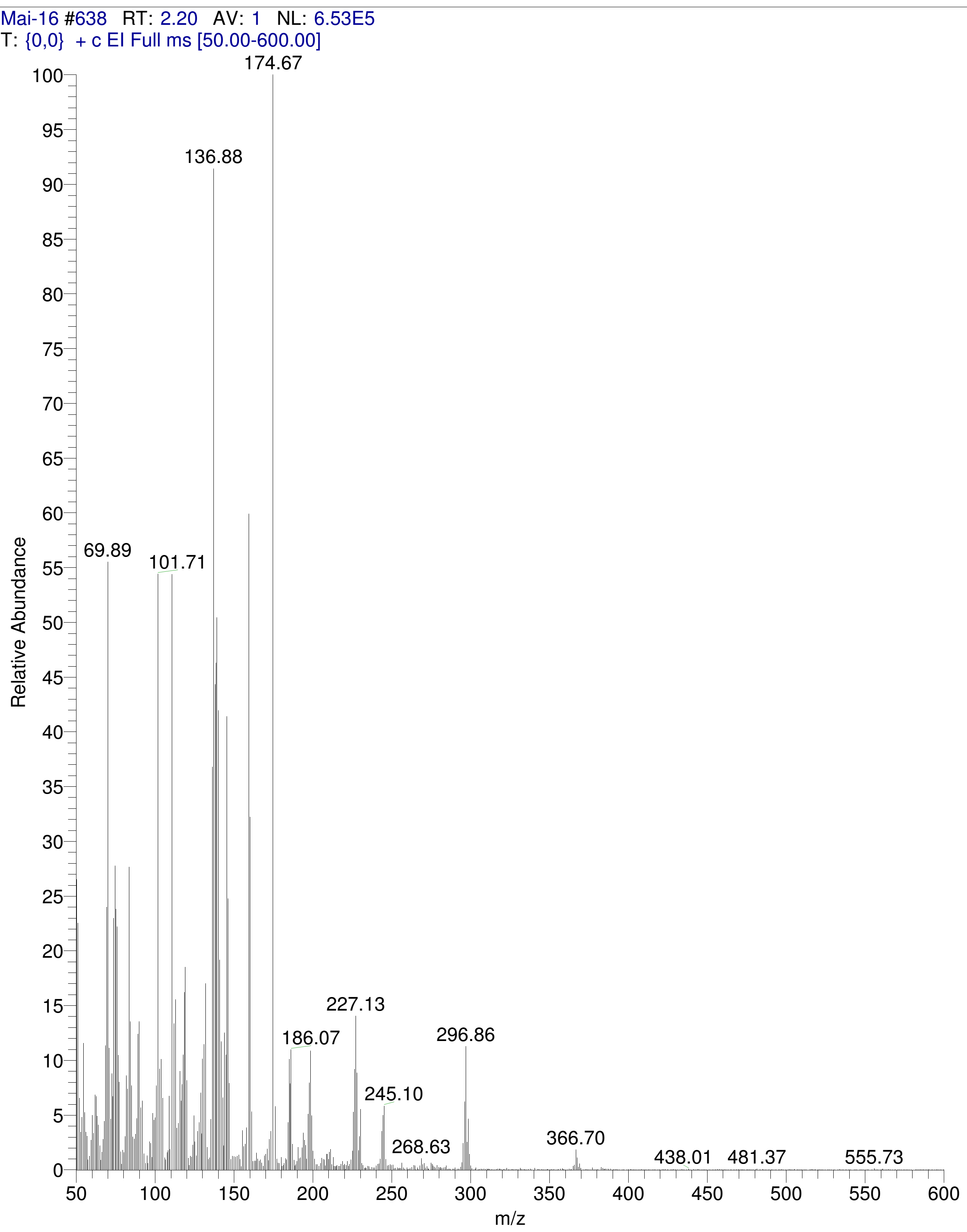


Mass spectrum for compound **16**


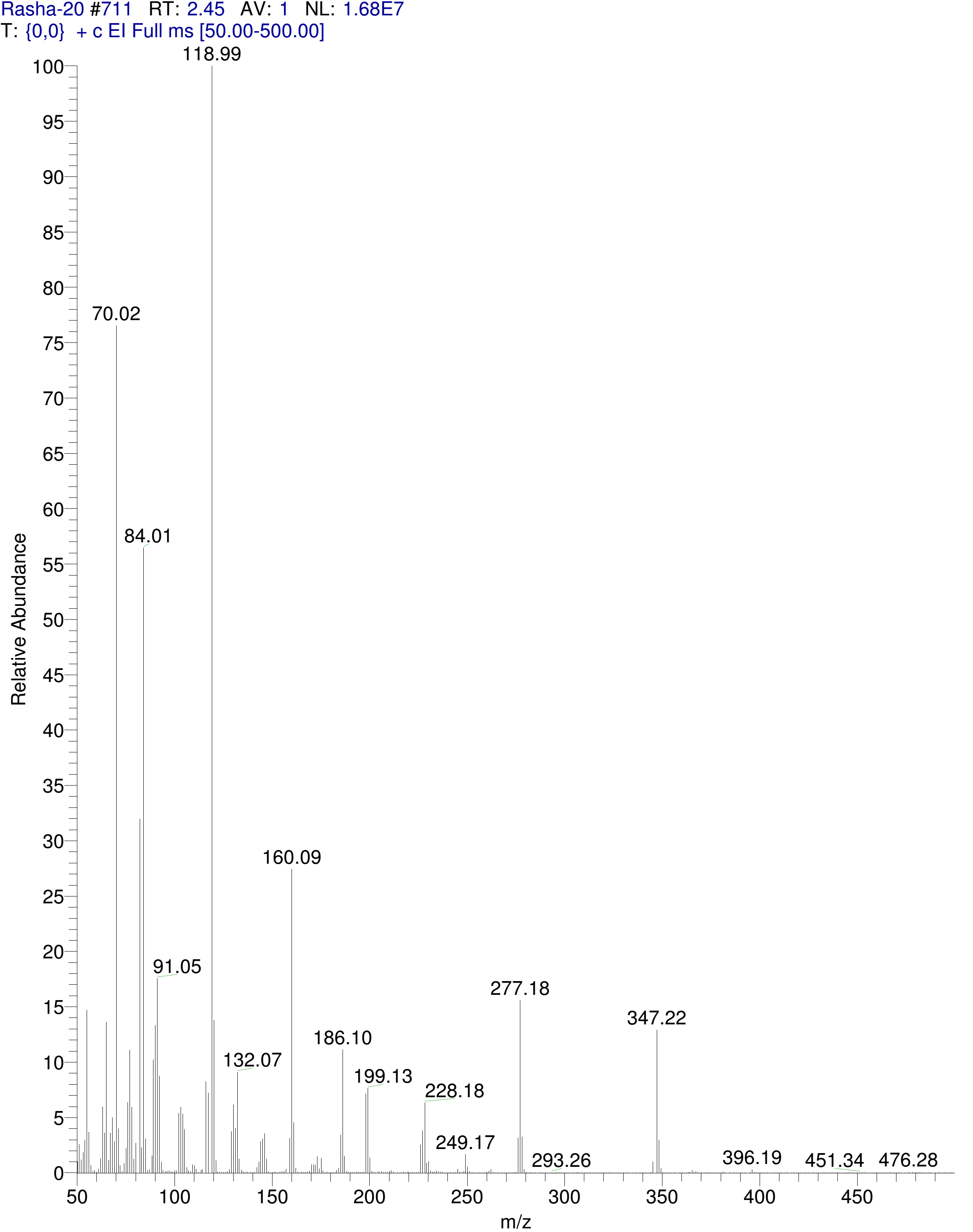


Mass spectrum for compound **17**


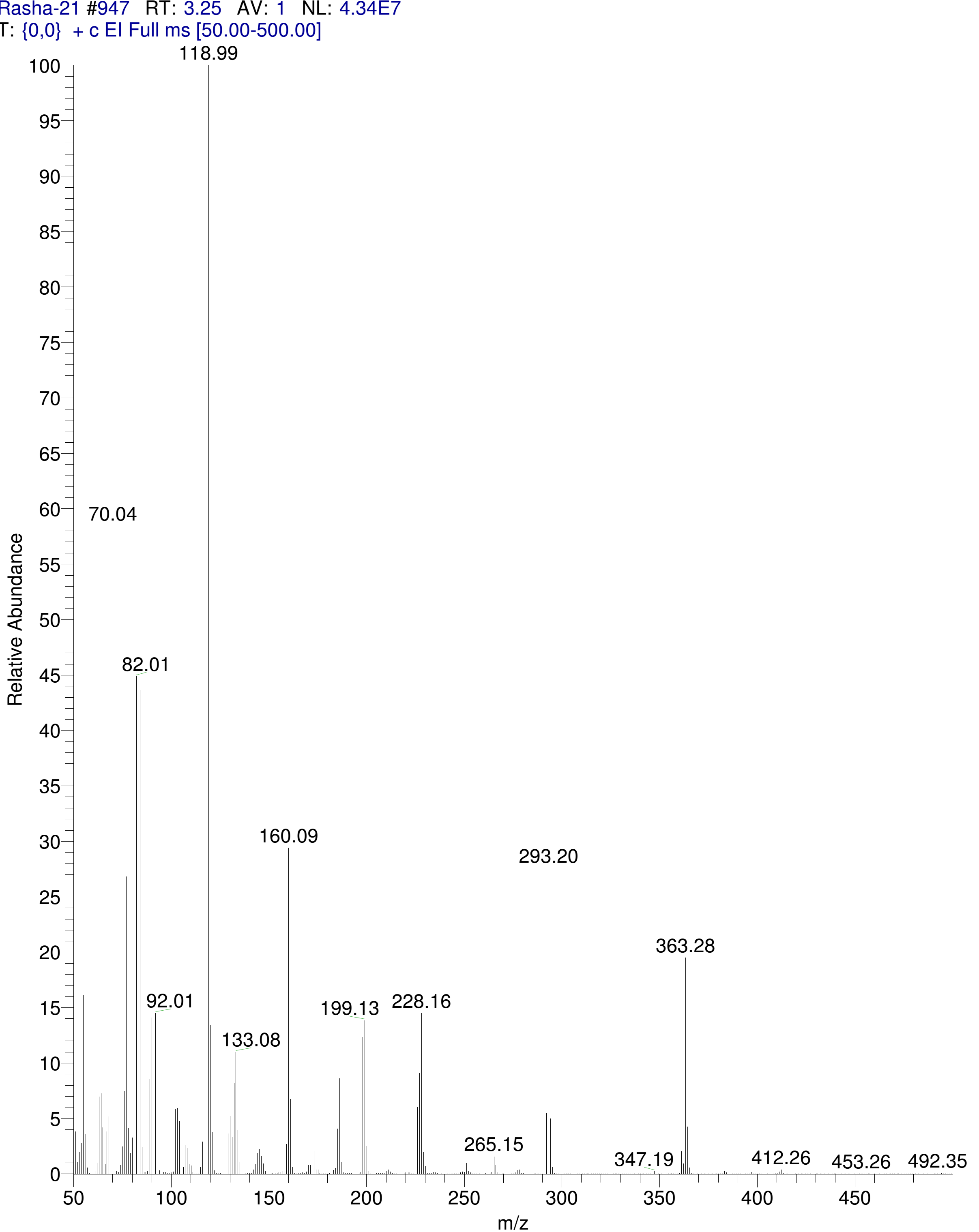


Mass spectrum for compound **18**


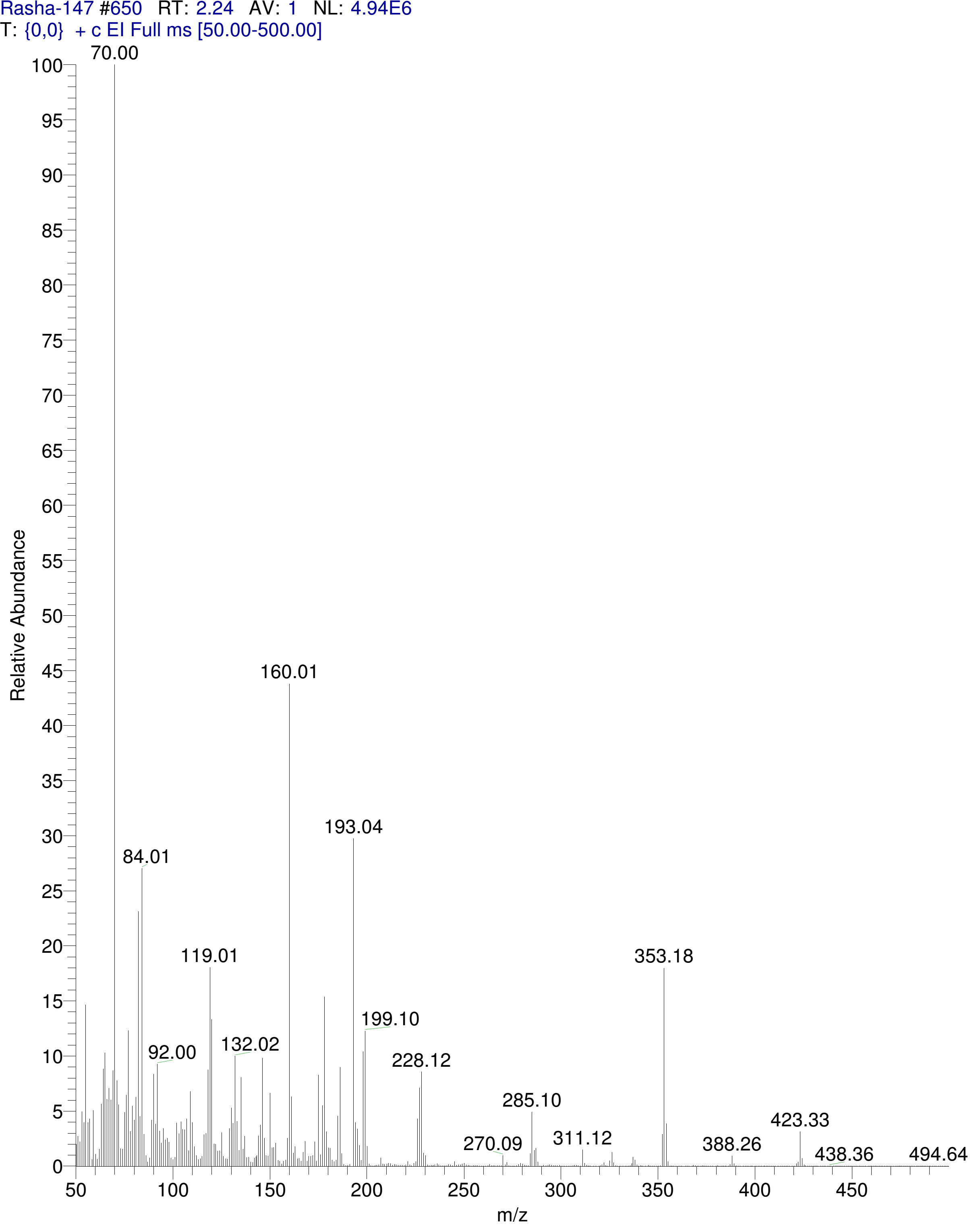


Mass spectrum for compound **19**


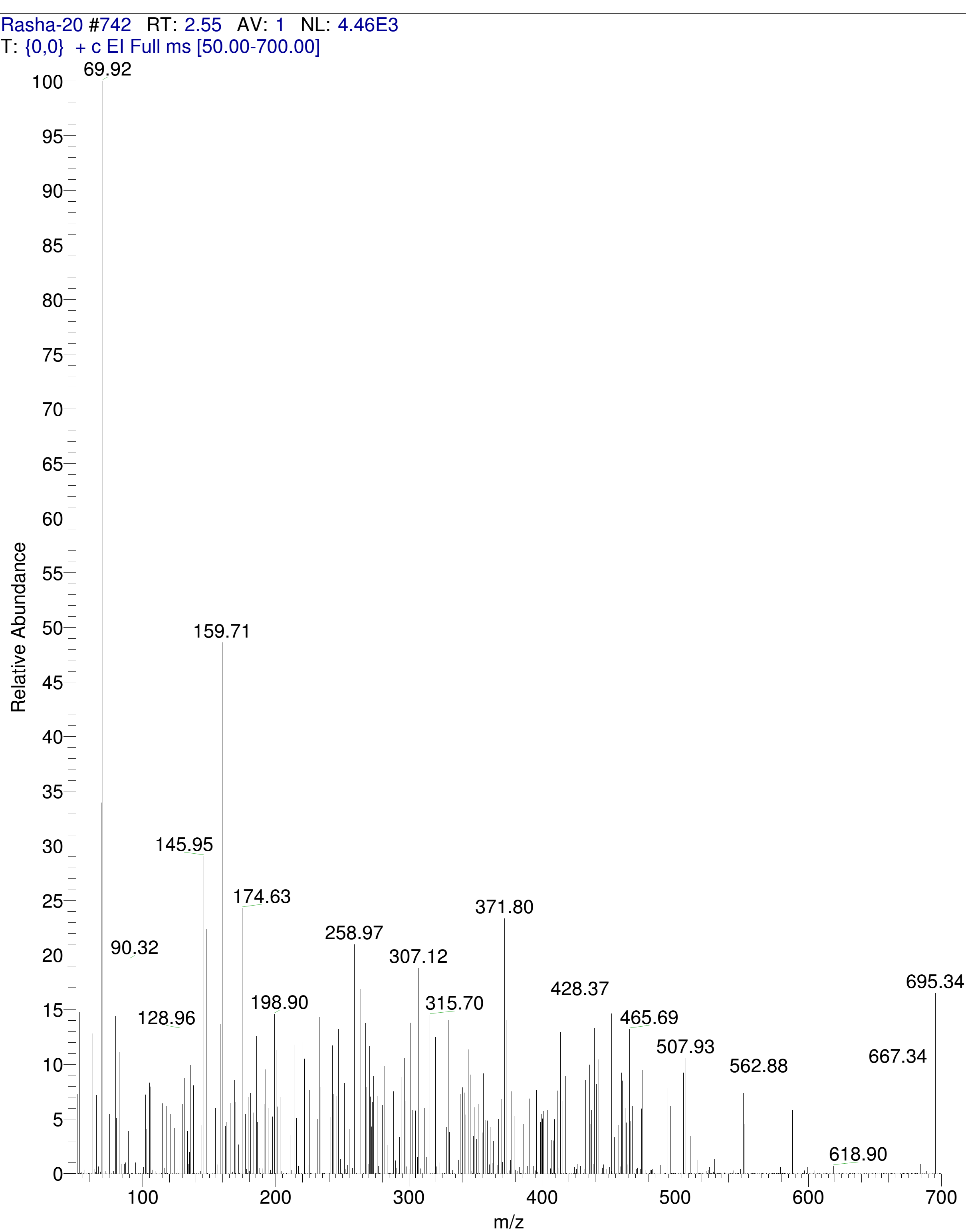


Mass spectrum for compound **20**


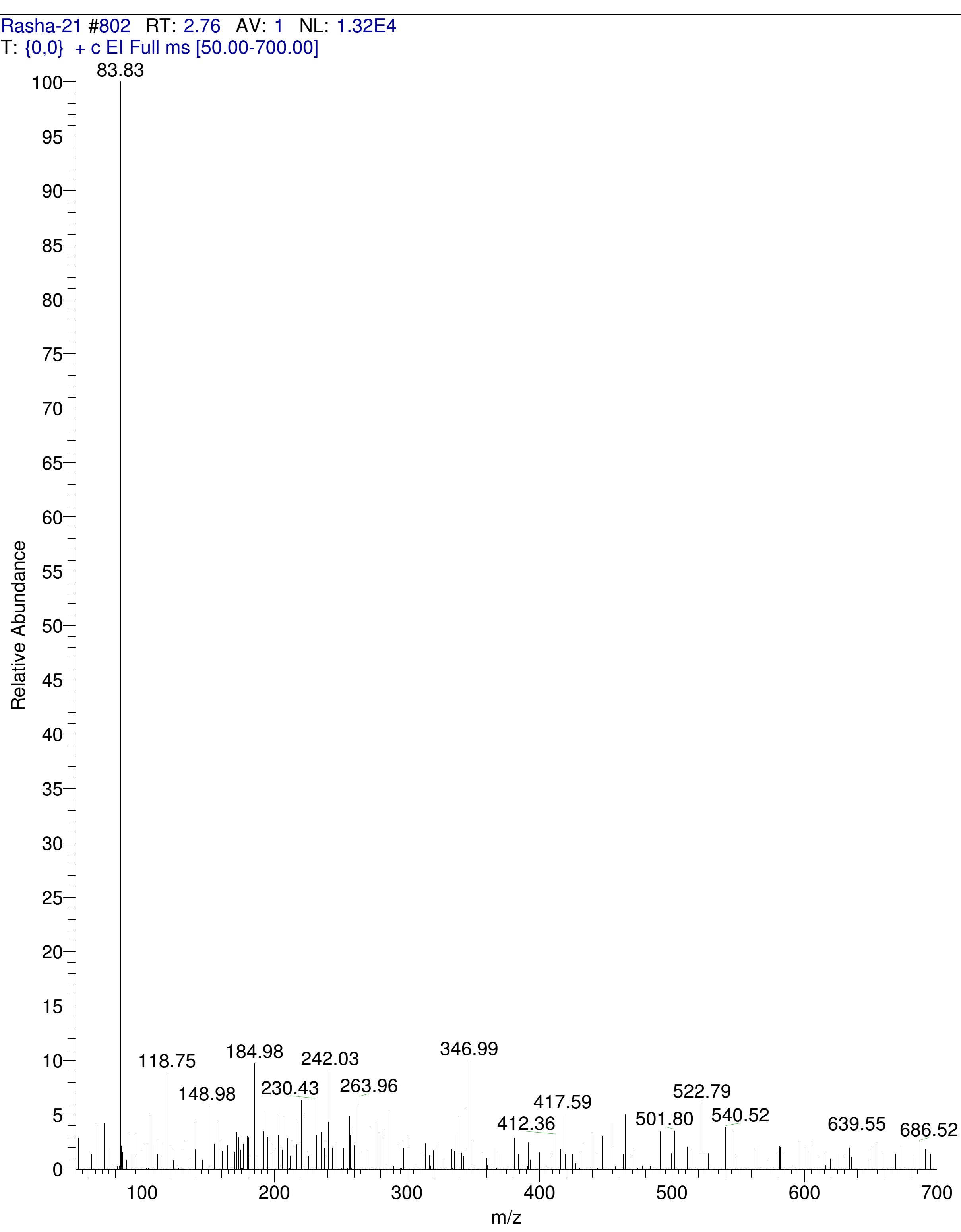


Mass spectrum for compound **21**


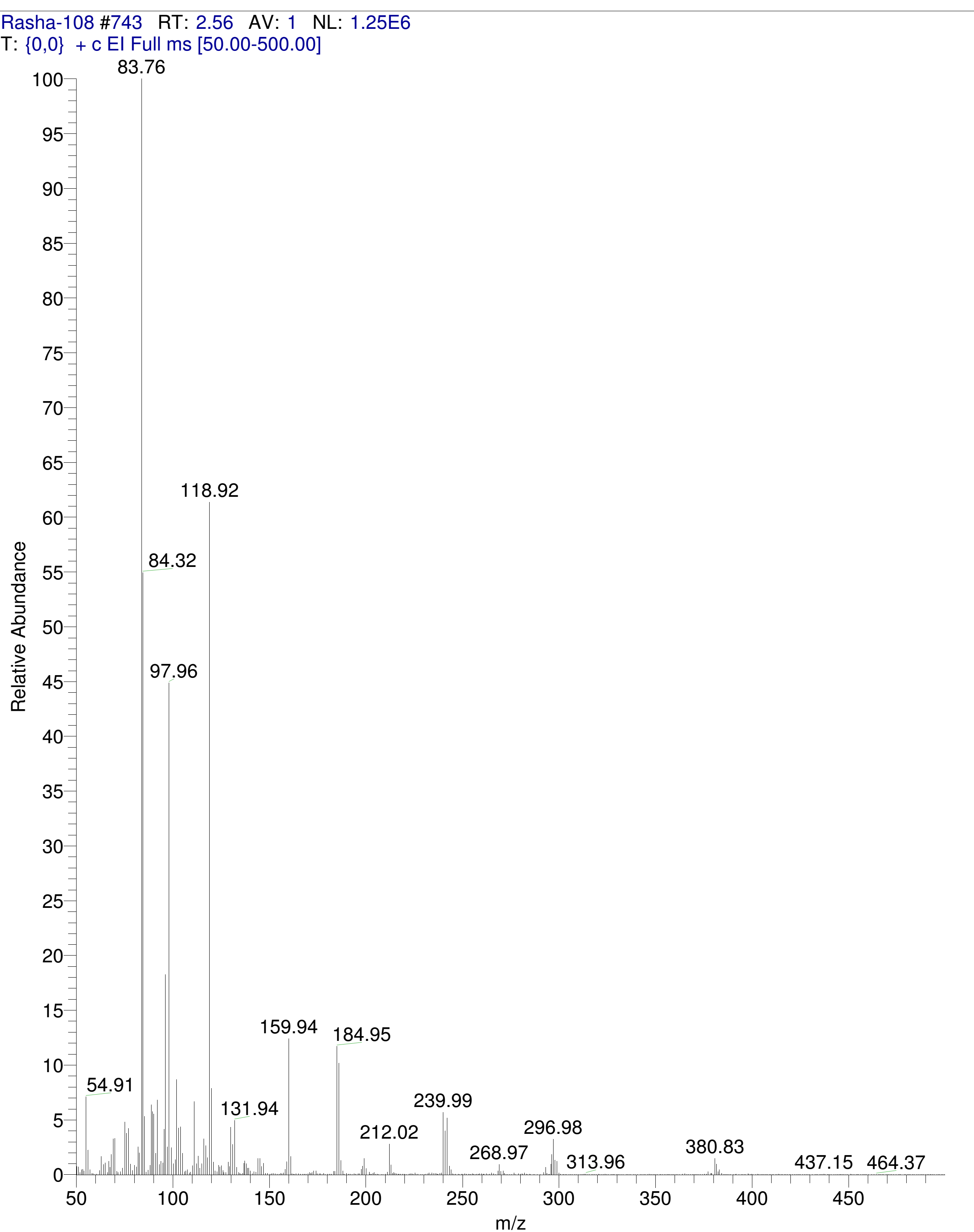


Mass spectrum for compound **22**


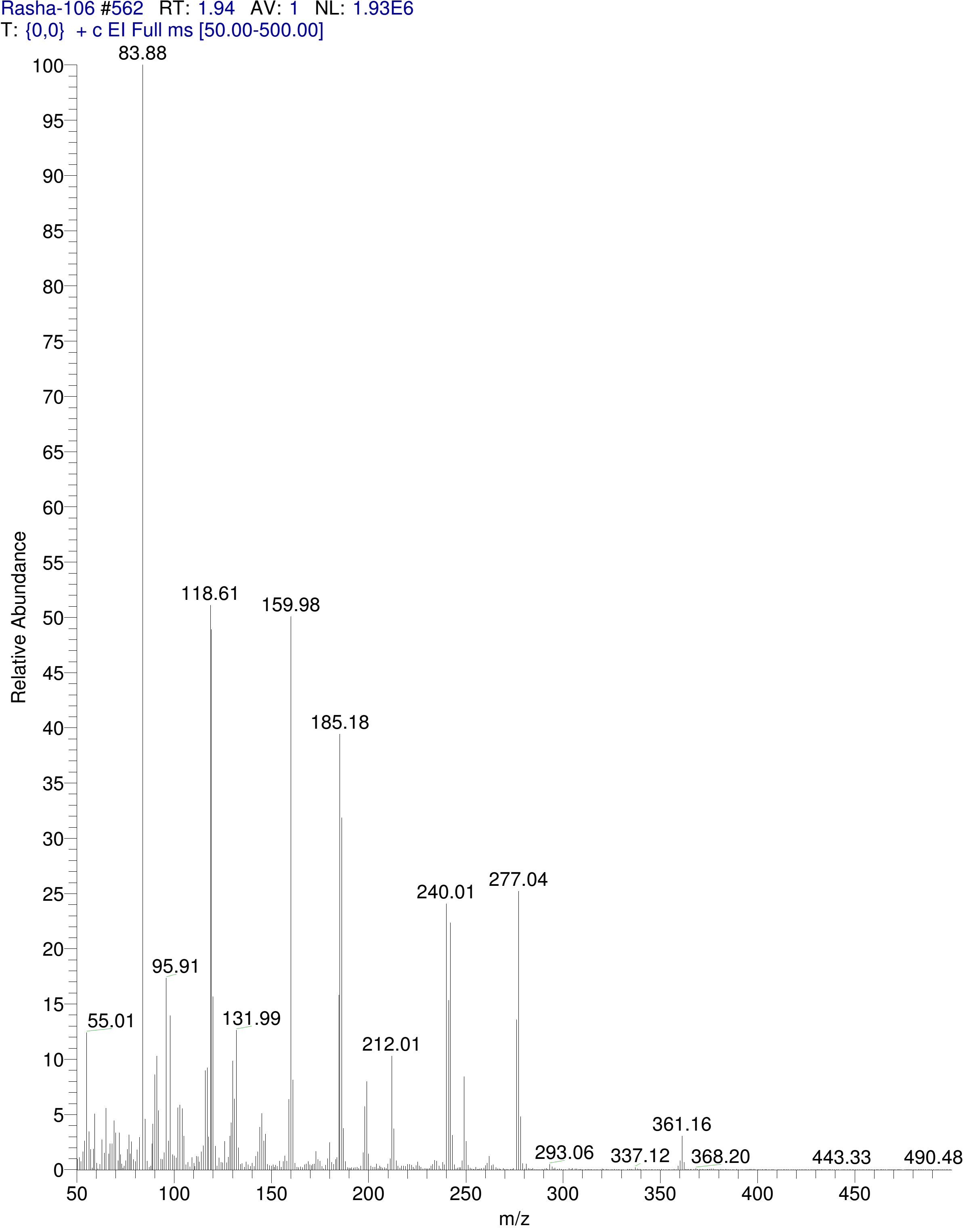


Mass spectrum for compound **23**


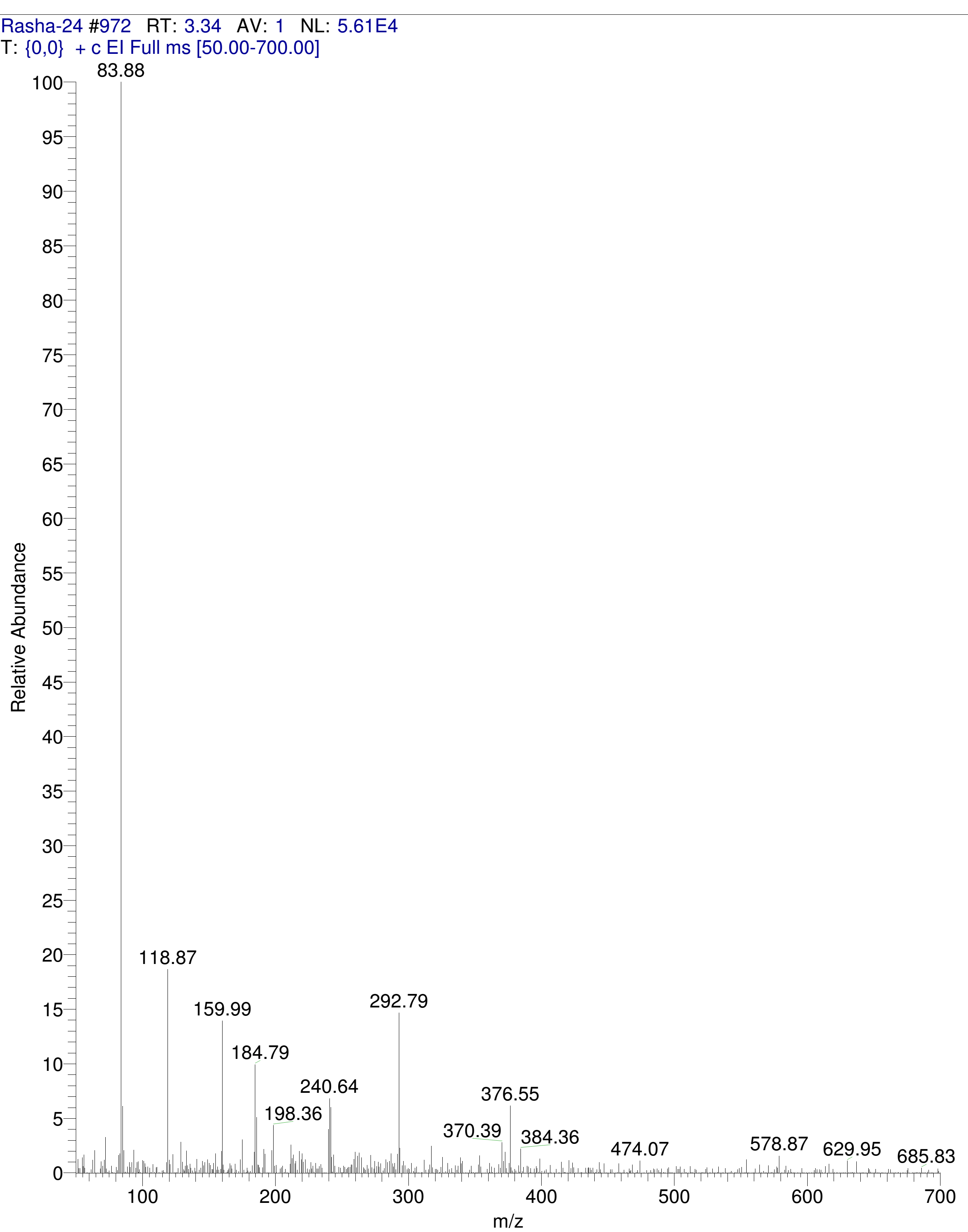


Mass spectrum for compound **24**


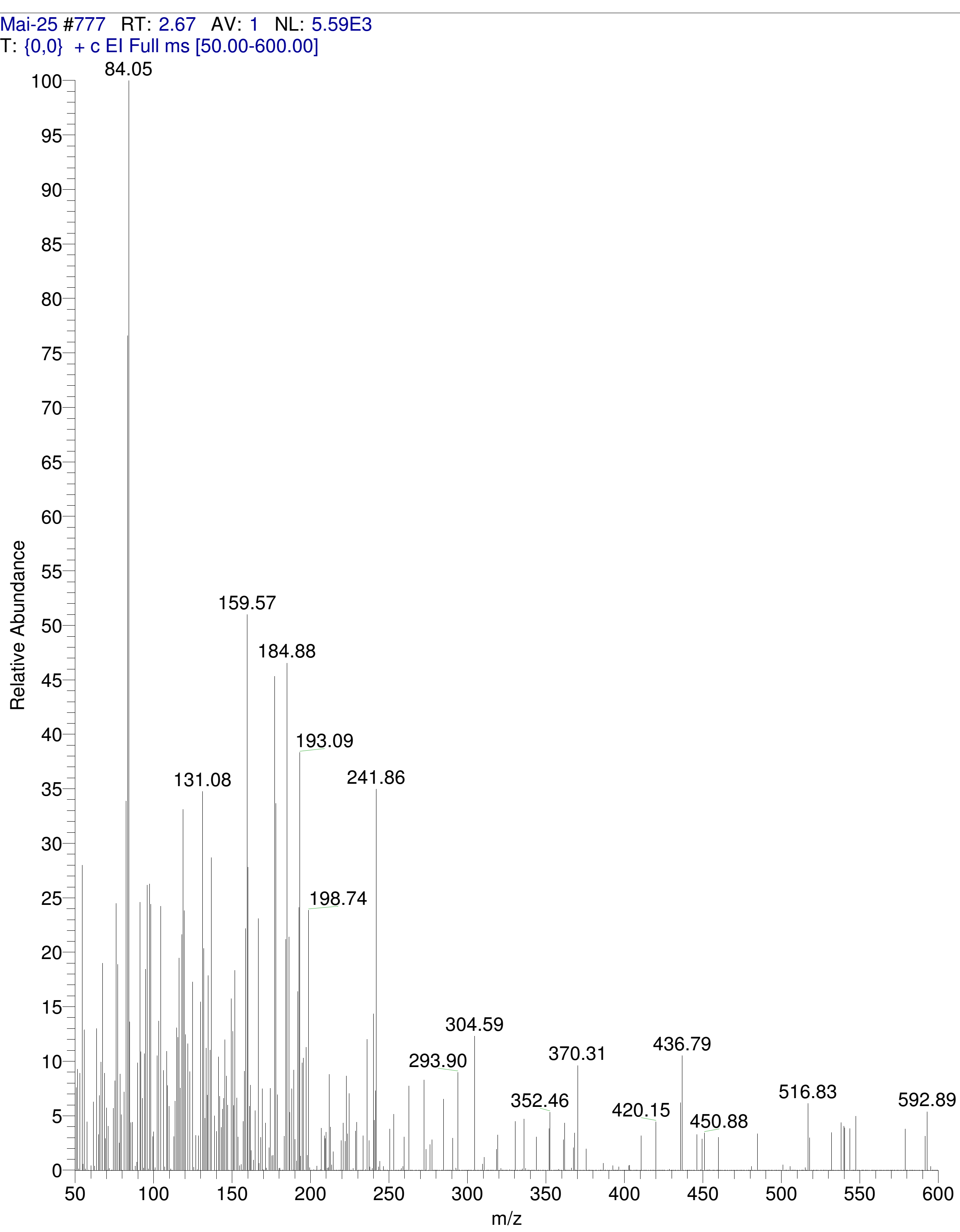


Mass spectrum for compound **25**


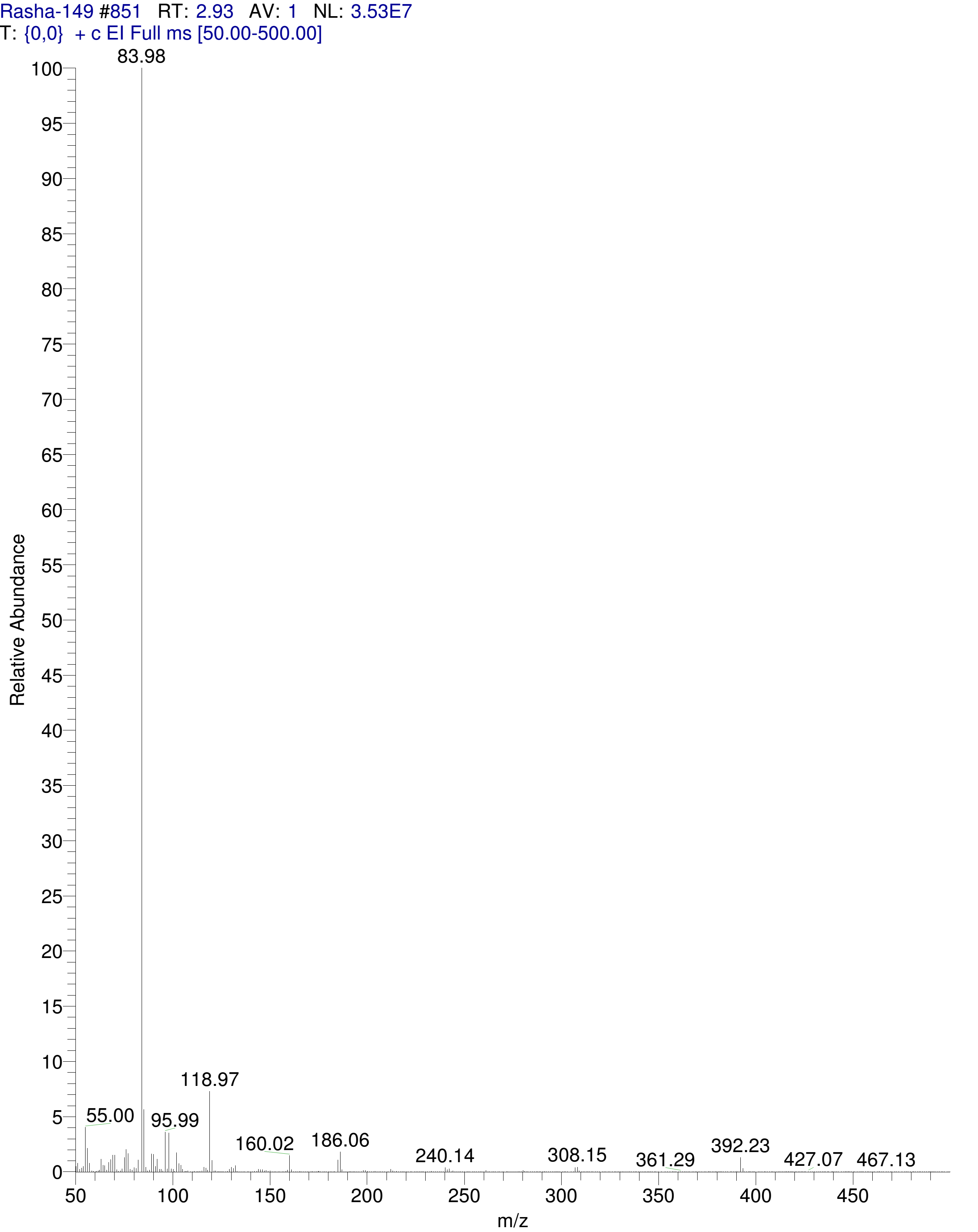


Mass spectrum for compound **26**

**
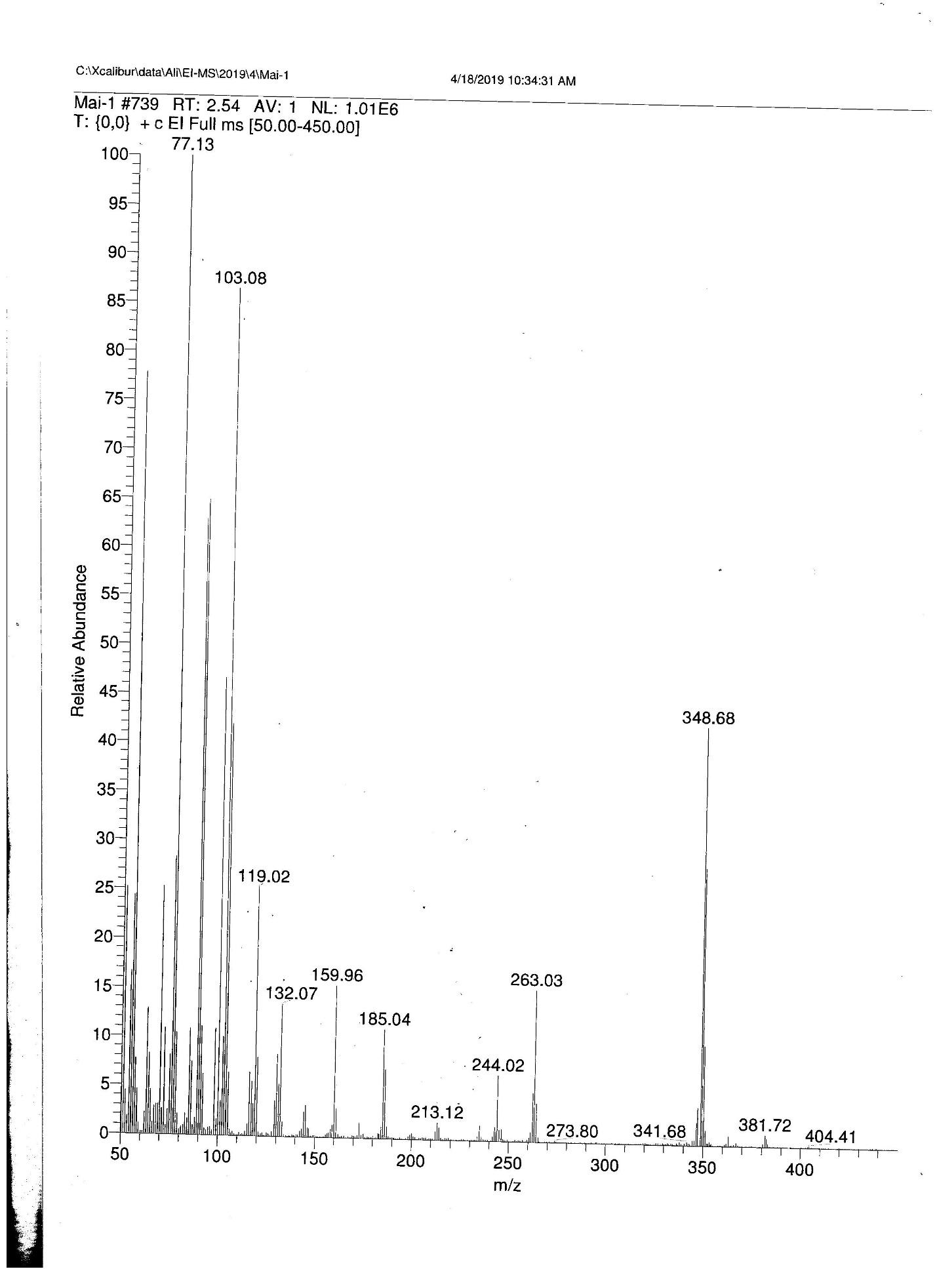
**

Mass spectrum for compound **27**


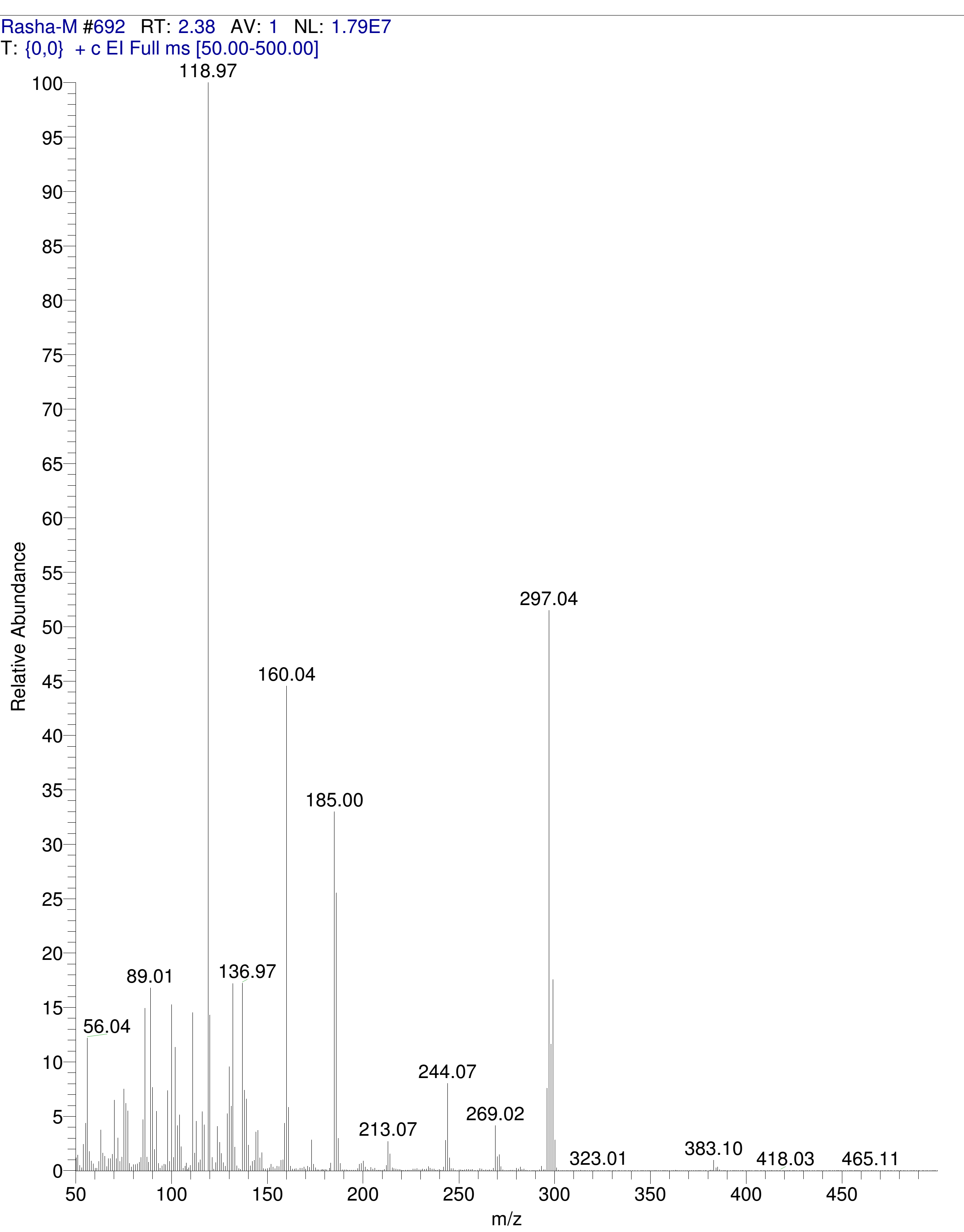


Mass spectrum for compound **28**


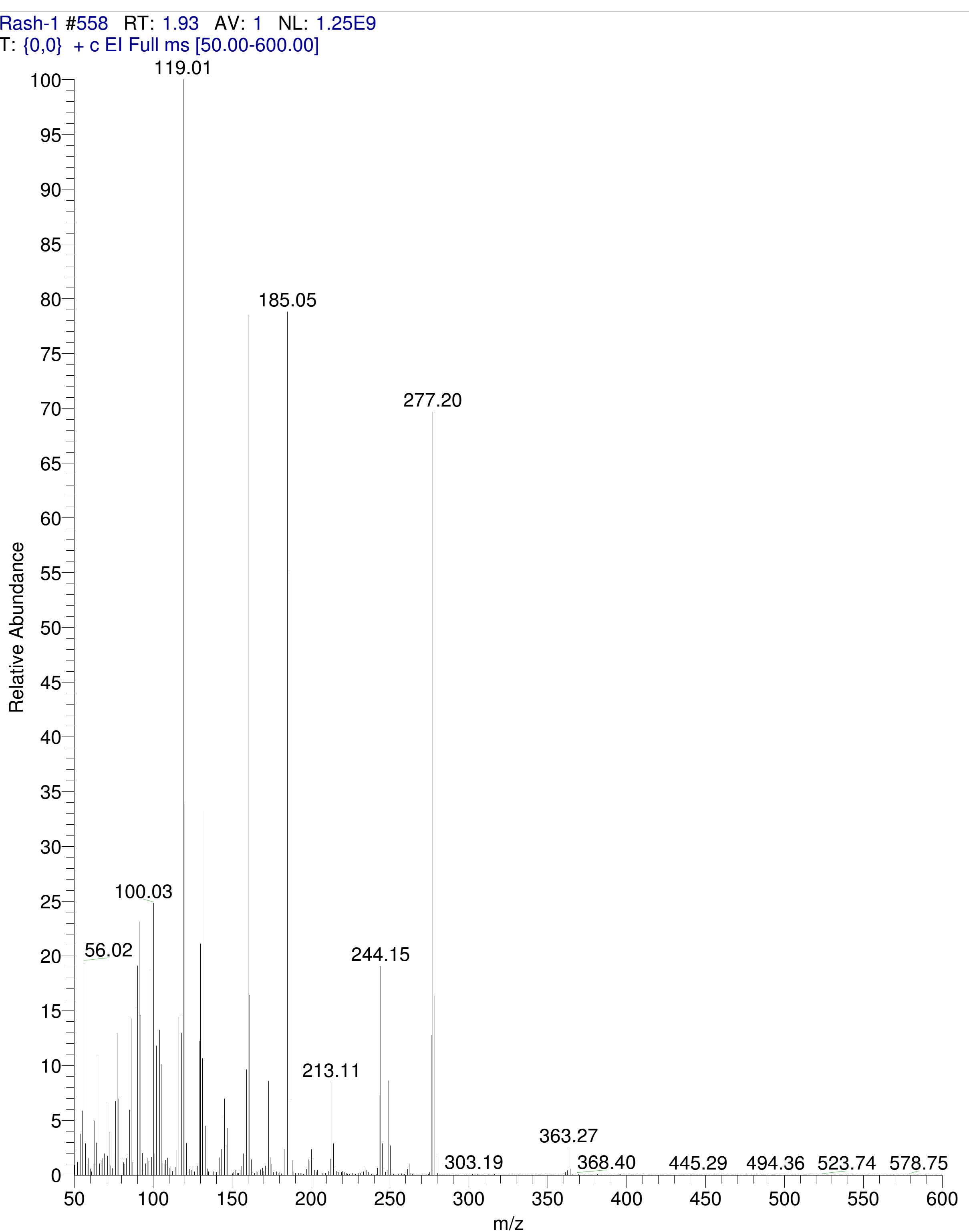


Mass spectrum for compound **29**


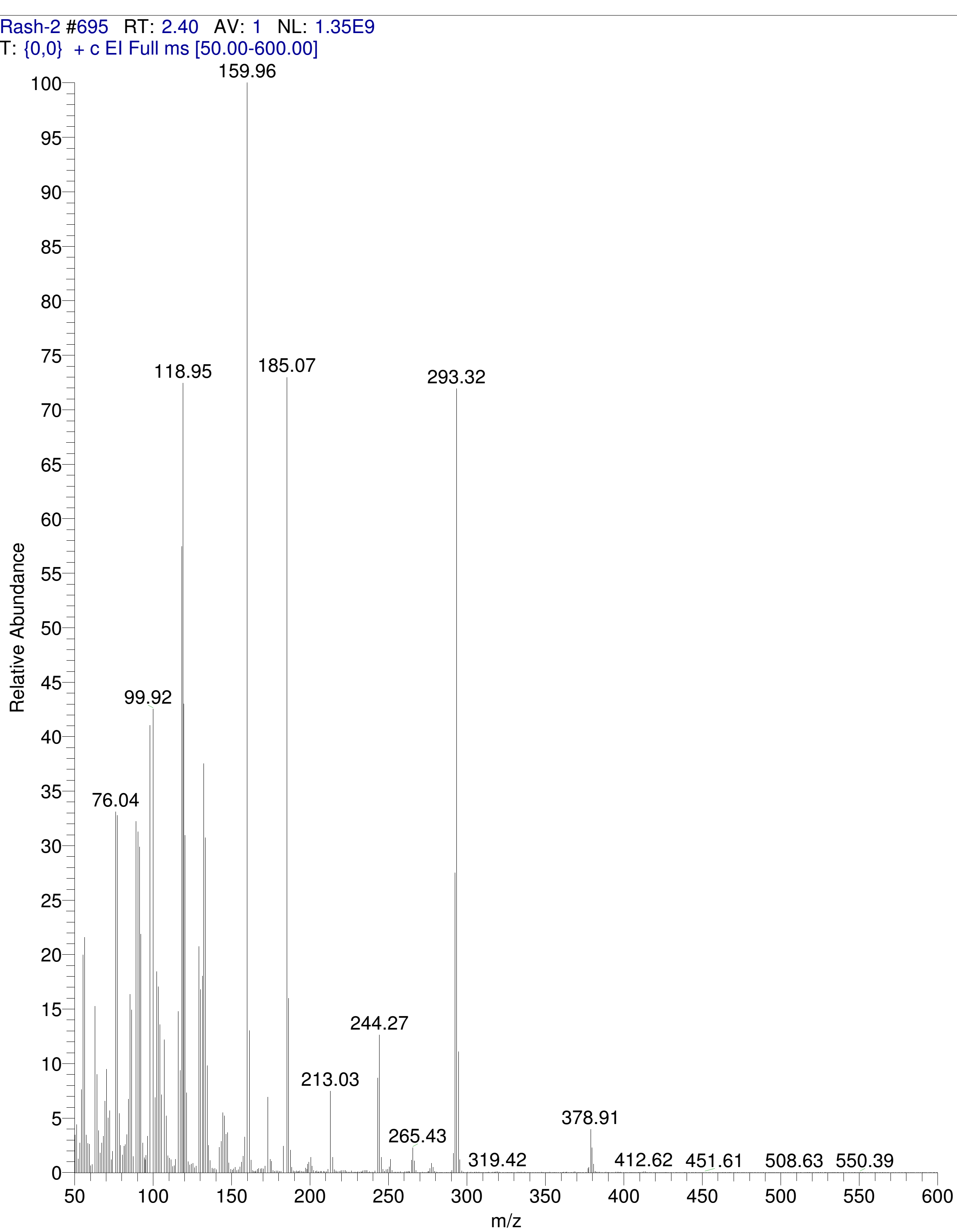


Mass spectrum for compound **30**


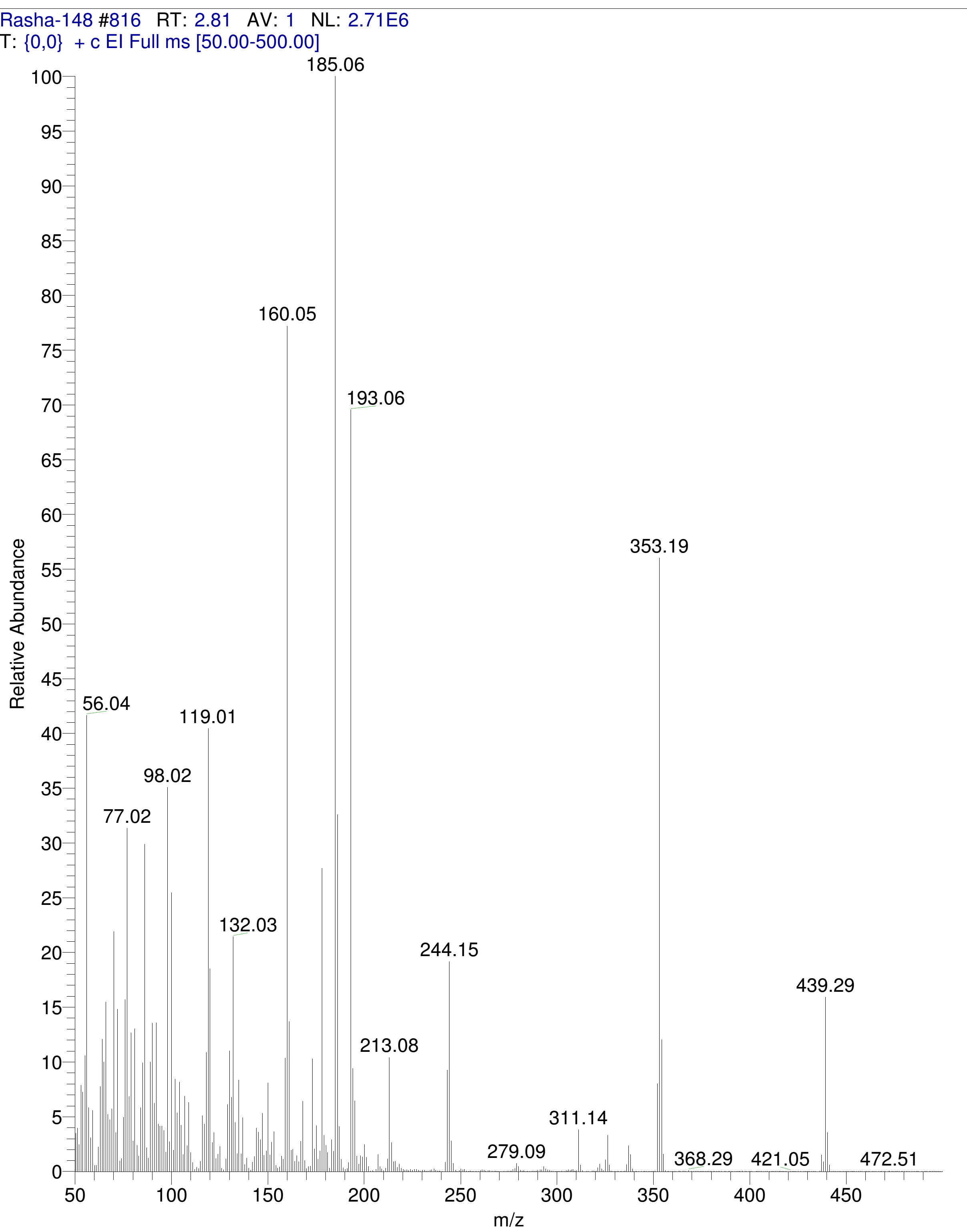


Mass spectrum for compound **31**


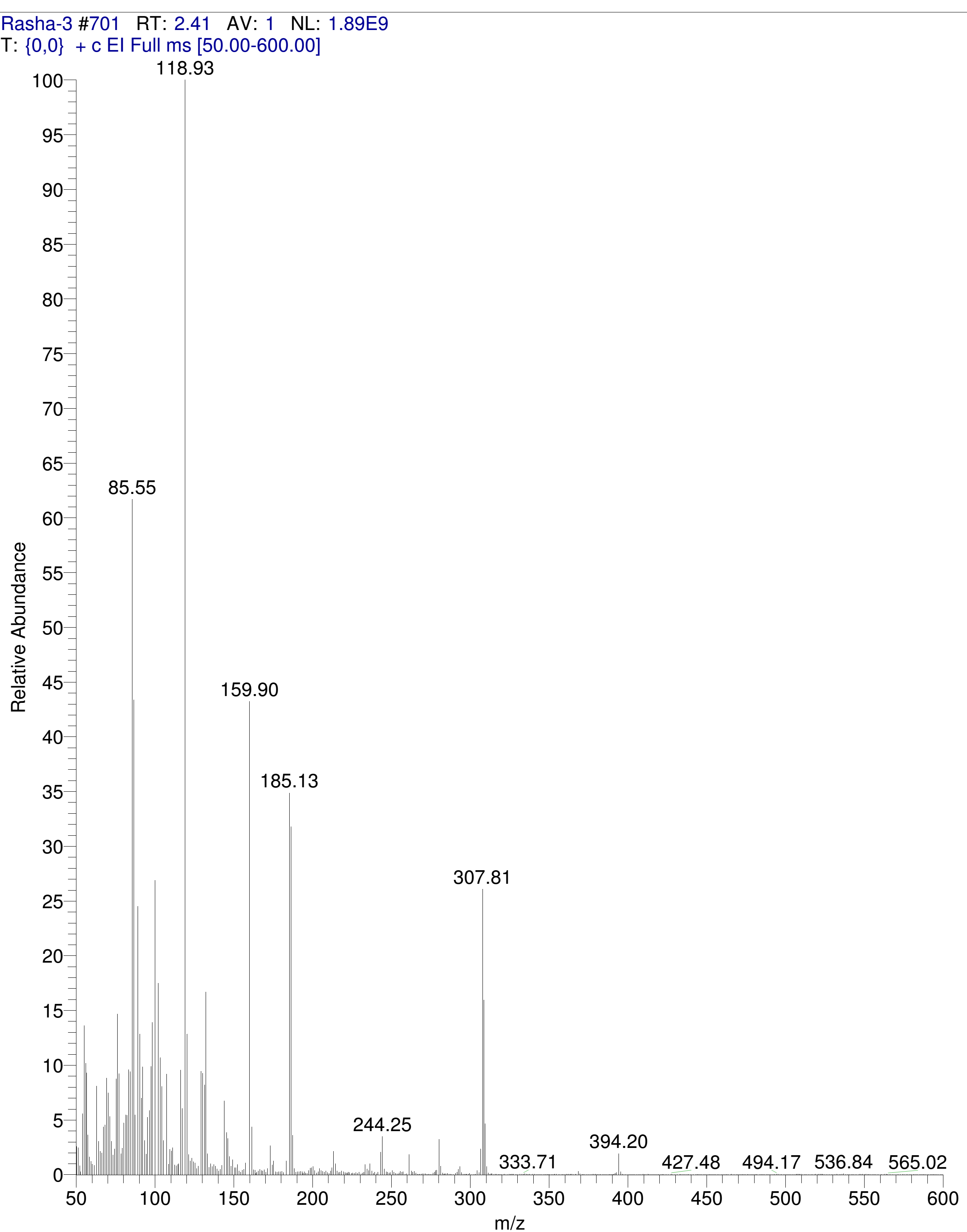


Mass spectrum for compound **32**

**HPLC chromatograms**

| **Compound** | **Chromatogram** |
| --- | --- |
| **9** |  |
| **10** |  |
| **11** |  |
| **12** |  |
| **13** |  |
| **14** |  |
| **15** |  |
| **16** |  |
| **17** |  |
| **18** |  |
| **19** |  |
| **20** |  |
| **21** |  |
| **22** |  |
| **23** |  |
| **24** |  |
| **25** |  |
| **26** |  |
| **27** |  |
| **28** |  |
| **29** |  |
| **30** |  |
| **31** |  |
| **32** |  |
